# Low-dose prednisolone and B vitamins for post-COVID-19 syndrome in Germany: the PreVitaCOV randomised clinical trial

**DOI:** 10.1016/j.eclinm.2026.104264

**Published:** 2026-10-24

**Authors:** Ildikó Gágyor, Andreas Klug, Alexander Schwager, Götz Gelbrich, Stefanie Joos, Cornelia Fießler, Karl Georg Haeusler, Laura Lunden, Catharina Escales, Juliane Döhring, Celine Kutterer, Yvonne Kaußner, Walter Maetzler, Oliver Zolk, Christian Förster, Hanna Kaduszkiewicz

**Affiliations:** a Department of General Practice, University Hospital Würzburg (UKW), Josef-Schneider-Straße 2, Building D7, Würzburg, 97080, Germany; b Institute for Clinical Epidemiology and Biometry, University of Würzburg, Würzburg, 97080, Germany; c Coordination Center for Clinical Trials, University Hospital Würzburg, Würzburg, 97078, Germany; d Institute of General Practice and Interprofessional Care, University Hospital Tübingen, Osianderstraße 5, Tübingen, 72076, Germany; e Department of Neurology, University Hospital Ulm, Oberer Eselsberg 45, Ulm, 89081, Germany; f Institute of General Practice, University of Kiel, Kiel, Germany; g Counseling Center for Employees, University Hospital Würzburg, Robert-Koch-Straße 34, Würzburg, 97080, Germany; h Department of Neurology, University Hospital Schleswig-Holstein and University of Kiel, Kiel, Germany; i Brandenburg Medical School, Faculty of Health Sciences Brandenburg, Institute of Clinical Pharmacology, Immanuel Klinik Rüdersdorf, Seebad 82/83, Rüdersdorf, 15562, Germany

**Keywords:** Post-COVID-19 syndrome, Randomised controlled trial, Prednisolone, B vitamins

## Abstract

**Background:**

Post-COVID-19 syndrome is burdensome and not yet adequately treatable, with persistent symptoms including fatigue, dyspnoea, and cognitive impairment. The Pan American Health Organisation recommends rigorous evaluation of potentially beneficial interventions. We investigated the efficacy and safety of prednisolone and vitamins B1, B6, and B12 in patients with post-COVID-19.

**Methods:**

This randomised, double-blind, placebo-controlled phase 3 trial (2 × 2 factorial design) was conducted in adults with post-COVID-19 at three university sites in Germany. Adult patients with persistent symptoms after a documented SARS-CoV-2 infection ≥12 weeks ago and without acute COVID-19, major comorbidities, pregnancy or breastfeeding, prior intensive care treatment for COVID-19, recent use of trial drugs, or potentially interacting concomitant medications were included. From January 13, 2023 until July 25, 2024 participants were randomly assigned to 28 days of once-daily oral treatment with prednisolone (20 mg on days 1–5, 5 mg on days 6–28), B vitamins (thiamine nitrate [B1], 100 mg, pyridoxine hydrochloride [B6] 50 mg, and cyanocobalamin [B12] 500 μg), prednisolone and B vitamins, or placebo. The primary outcome was change in overall symptom severity from baseline to day 28, assessed in the intention-to-treat population (participants who received at least one dose of study drug) at the first follow-up visit after randomisation with a customised Patient-Reported Outcomes Measurement Information System (PROMIS) total T-score. A change of ≥3 points was considered clinically meaningful. Efficacy was assessed using a pre-specified factorial analysis of the main effects of prednisolone and B vitamins. Safety was assessed by analysing frequency and severity of adverse events in all randomised patients (safety population). This completed trial is registered with ClinicalTrials.gov (NCT05638633).

**Findings:**

Of 2498 patients assessed for eligibility, 323 were randomised and 310 patients had evaluable data for the outcomes. The mean age was 44 (standard deviation (SD) 13) years, 200 participants were women. The mean change in PROMIS T-score was −4·3 (SD 5·1) with prednisolone, −3·2 (SD 5·0) with B vitamins, −4·5 (SD 4·5) with prednisolone plus B vitamins, and −2·9 (SD 4·6) with placebo. The adjusted mean difference between participants treated with prednisolone and those not treated was −1·4 points (95% confidence interval (CI), −2·5 to −0·4; p = 0·008), driven primarily by the change in the fatigue subscore (−6·4 points, p < 0·001). B vitamins showed no statistically significant or clinically meaningful difference compared with no B vitamins (adjusted mean difference (MD), −0·1 points; 95% CI, −1·2 to +1·0; p = 0·85). Adverse events were equally distributed between groups, with no study-drug-related serious adverse events.

**Interpretation:**

This trial showed a statistically significant short-term reduction in symptom severity in the five dimensions of the PROMIS score in patients with post-COVID with low-dose prednisolone. However, the effect did not reach the prespecified threshold for clinical relevance. Further research is needed to confirm the efficacy of prednisolone and to determine the optimal dose and treatment duration while balancing benefits against longer-term adverse effects.

**Funding:**

German Federal Ministry of Research, Technology and Space, Vogel Stiftung Dr. Eckernkamp, Hevert-Arzneimittel GmbH & Co. KG.

**Research in context**

**Evidence before this study**

Post-COVID-19 syndrome, also referred to as long-COVID, has emerged as a major public health challenge, characterised by persistent, heterogeneous symptoms, most prominently fatigue, dyspnoea, and cognitive complaints. Despite the high prevalence and burden of disease, evidence-based pharmacological treatment options remain scarce. We searched PubMed for publications from 1 January 2020 to 15 January 2026, with no language restrictions, to identify clinical trials evaluating therapy with corticosteroids or B vitamins in patients with post-COVID using the search term (“Post-Covid condition” OR PASC OR “post-covid-19 syndrome” OR “long-covid”) AND (prednisolone OR corticosteroids OR “vitamin B”) AND trial. This search yielded nineteen publications; none reported therapeutic randomised controlled trials in post-COVID populations, and no pooled estimates from meta-analyses were available.

However, therapeutic approaches have been proposed as plausible candidates. Corticosteroids have been suggested for formal evaluation based on their anti-inflammatory and immunomodulatory effects, given evidence of immune dysregulation and persistent inflammation in subsets of patients with post-COVID. Similarly, supplementation with vitamins B1, B6, and B12 has been discussed due to their established neurotropic roles and potential relevance for neurological and fatigue-related symptoms. Existing evidence before this study was limited to small, uncontrolled observational reports suggesting symptomatic improvement—particularly in fatigue and dyspnoea—after corticosteroid treatment, while no clinical evidence supported the use of B vitamins for post-COVID symptoms.

**Added value of this study**

Our study is the first randomised, double-blind, placebo-controlled factorial phase 3 trial to evaluate therapy with prednisolone and B vitamins—both individually and in combination—in patients with post-COVID-19 syndrome. The study provides evidence that a 28-day course of prednisolone leads to a statistically significant reduction in overall symptom severity compared with no prednisolone, with the effect primarily driven by improvement in fatigue. However, the observed between-group difference did not reach the prespecified clinically meaningful threshold. Therapy with vitamins B1, B6, and B12 did not demonstrate any efficacy. By addressing a major evidence gap with a rigorous trial design, this study provides important evidence to inform the clinical evidence base for pharmacological management of post-COVID symptoms.

**Implications of all the available evidence**

Our findings suggest that short-term treatment with prednisolone may represent a pragmatic and accessible therapeutic option for patients with post-COVID-19 syndrome, particularly those with prominent fatigue, although the observed effect on overall symptom severity did not reach the prespecified threshold for clinical meaningfulness. Potential benefits must therefore be weighed carefully against known risks of systemic corticosteroids, including metabolic, cardiovascular, and neuropsychiatric adverse effects. Given that the treatment effect was consistently observed for fatigue, future trials may consider a more targeted primary endpoint focused on fatigue alone. Further research should confirm these findings in larger and more diverse populations and determine optimal dosing, treatment duration, long-term safety, and durability of response.

## Introduction

Post-COVID-19 syndrome (post-COVID), also referred to as post-COVID condition or long-COVID, is defined by symptoms emerging during or after a SARS-CoV-2 infection and lasting more than 12 weeks.^1^ It has more recently been characterised by the National Academies of Sciences, Engineering, and Medicine as an infection-associated chronic condition that persists for at least three months and affects one or more organ systems.^2^ Prevalence estimates for post-COVID vary substantially across studies due to the chosen definition of post-COVID, the underlying population, circulating virus variants, and methods of symptom assessment. A global modelling study estimated that about 6% of individuals with symptomatic SARS-CoV-2 infection experienced at least one post-COVID symptom cluster 3 months after infection (fatigue, respiratory, or cognitive).^3^ Common symptoms include fatigue, cognitive dysfunction, and shortness of breath, and generally have an impact on everyday life.^1^ Post-COVID is associated with a lower quality of life^4^ and a reduction in the ability to work, which illustrates the high individual and societal burden of the illness.^5^ To date, no evidence-based treatment specifically targeting post-COVID has been established. Both nirmatrelvir-ritonavir and colchicine demonstrated no significant improvements in treating post-COVID in randomised controlled trials, indicating the need for alternative therapeutic approaches.^6^^,^^7^ Systematic reviews on post-COVID treatments similarly demonstrate a current lack of robust evidence supporting most proposed drug therapies.^8^^,^^9^ This highlights the need for high-quality randomised-controlled drug trials for post-COVID.^10^^,^^11^ Among pharmacological candidates for post-COVID, corticosteroids, e.g. low-dose prednisolone, have been explicitly proposed for formal testing in open-label or randomised trials for their immunosuppressive and anti-inflammatory effects.^12^^,^^13^ The supplementation of vitamins B1, B6, and B12 (hereinafter referred to as B vitamins) has also been discussed as an option due to their neurotropic properties, supporting nervous system function.^14^^,^^15^ Both are well-known drugs with a reliable efficacy and safety profile and they are feasible for administration in primary care.^16^^,^^17^ In fact, small observational studies without control groups showed an improvement in clinical symptoms such as dyspnoea and fatigue in patients with post-COVID after treatment with corticosteroids.^18^, ^19^, ^20^ To the best of our knowledge, there is currently no evidence supporting the efficacy of vitamins B1, B6, and B12 in treating post-COVID symptoms. For both corticosteroids and B vitamins, there are no published randomised controlled trials (RCTs) investigating the effectiveness of these drugs. This is the first randomised placebo-controlled trial to evaluate the efficacy and safety of a short-term prednisolone therapy and supplementation of B vitamins—both alone and in combination—in patients with post-COVID.

## Methods

### Study design and participants

This multicentre, randomised, double-blind, placebo-controlled, parallel-group, phase 3 clinical trial with a 2 × 2 factorial design was conducted at the Departments of General Practice at the Universities of Würzburg, Tübingen, and Kiel in Germany. The study steering committee, comprising two coordinators from each study site (IG, AK, SJ, CFö, HK, and LL until December 2023, thereafter JD), oversaw the conduct and progress of the study through regular meetings. The trial comprised a pilot and a confirmatory phase. The Data and Safety Monitoring Board approved progression to the confirmatory phase after confirming recruitment and retention feasibility for at least 100 participants and reviewing the safety of the study drugs. The pilot phase did not result in any changes to the confirmatory protocol. Ambulatory adult patients with a documented SARS-CoV-2 infection at least 12 weeks before study inclusion and presenting with at least one of the symptoms—fatigue, dyspnoea, cognitive impairment, anxiety or depression—developed during or after infection were considered eligible. Inclusion required either a polymerase chain reaction (PCR) confirmed infection or a positive antibody test. Key exclusion criteria were a history of intensive care treatment for COVID-19, acute COVID-19, pregnancy, breastfeeding, diabetes mellitus, severe comorbid conditions, current or previous treatment with any of the study drugs for seven or more days since COVID-19, as well as any parenteral use of these drugs and concomitant medications that could interact with the study drugs. Following approval of protocol amendment 1 by the lead ethics committee (2 May 2023) and the Federal Institute for Drugs and Medical Devices (BfArM, 5 June 2023), eligibility criteria were amended to accept confirmation of the SARS-CoV-2 infection by the patient's general practitioner in addition to PCR or antibody tests, to allow limited prior oral treatment with vitamins B1, B6, or B12 (stopped ≥4 weeks before inclusion), and to remove arterial hypertension as an exclusion criterion. Following approval of amendment 2 (BfArM 4 October 2023, lead ethics committee 5 October 2023), intake of oral contraceptives and angiotensin-converting enzyme (ACE) inhibitors was additionally permitted. These non-prespecified amendments were introduced to facilitate the inclusion of otherwise eligible patients. A summary of all protocol versions and amendments is provided in eTable S7 of the Supplementary Appendix. Participants were recruited by collaborating general practices or directly through public outreach by announcements in local newspapers and on social media platforms. Interested participants were contacted by the research nurse at each site, who provided information about the study, particularly about inclusion and exclusion criteria. Those probably eligible were then invited to the nearest study site, where, after providing written informed consent, they underwent a full eligibility assessment and baseline data collection.

### Randomisation and masking

For practical reasons, randomisation and dispensing of the trial medication occurred before safety laboratory results were available. If unexpected pathological values were found, according to the trial protocol, patients were excluded before the first dose and classified as screening failures. Patients were individually allocated to four treatment groups in a 1:1:1:1 ratio by stratified block randomisation with varying block sizes, using the study site as the single stratification factor. Randomisation lists were generated by a statistician (not involved in final data analysis) with the software PASS, Version 2022, and stored outside of the Trial Master File with no access for staff involved in enrolment or assignment to interventions. To maintain blinding, active treatments and placebos were identically encapsulated and packaged in sequentially numbered containers. Participants were not informed that B vitamins can cause visible discolouration of urine, which could theoretically serve as an unmasking cue. Participants, treating physicians, outcome assessors, and data analysts remained unaware of treatment allocation throughout the trial. At the final follow-up visit (day 180), participants and trial staff were asked to guess which treatment they believed had been administered in order to assess the success of blinding.

### Procedures

Participants received either prednisolone plus placebo, B vitamins plus placebo, prednisolone plus B vitamins, or placebo plus placebo. The prednisolone regimen consisted of 20 mg once daily for 5 days, followed by 5 mg once daily for 23 days. The 28-day treatment regimen and prednisolone dosing were based on clinical experience with short-term corticosteroid bursts used in inflammatory conditions, and whether a higher dose would have been equally tolerable while more effective remains to be established in future studies. B vitamins contained 100 mg of vitamin B1, 50 mg of vitamin B6, and 500 μg of vitamin B12 per day for 28 days. Participants were instructed to take one capsule containing prednisolone (20 mg for 5 days, followed by 5 mg for 23 days) or matching placebo and one tablet containing B vitamins or matching placebo each morning for 28 days, starting the day after the baseline visit. During the treatment period, patients were contacted by telephone on day 5 to assess symptom severity, new symptoms, and adverse events (AEs), including serious AEs (SAEs). In addition, participants documented symptom severity in a paper-based diary on days 14 and 21, along with information on the use of medication on demand and any changes in concomitant medication. On day 28, participants attended visit 2 for outcome and safety assessments and verification of drug accountability. Follow-up telephone calls were conducted two and six months after enrolment, respectively. All data were entered into an electronic case-report form (SecuTrial®).

### Outcomes

The primary outcome was the change in overall symptom severity from baseline to day 28, assessed using a study-specific composite score derived from the Patient-Reported Outcomes Measurement Information System (PROMIS) item banks and scored according to PROMIS guidelines for custom short forms.^21^^,^^22^ This was calculated for all active treatments (prednisolone, B vitamins and the combination of both) and placebo. The PROMIS total score was compiled to capture common symptoms of post-COVID and included the domains fatigue, dyspnoea, cognitive impairment, anxiety, and depression. Each contributing domain has been independently validated for construct validity, reliability, and responsiveness across a wide range of clinical populations, so no additional psychometric re-evaluation of the composite score was considered necessary for this trial.^23^, ^24^, ^25^ Pre-specified secondary outcomes included changes from baseline to day 28 in the PROMIS subscores; the Measure Yourself Medical Outcome Profile (MYMOP®)^26^; a post-COVID symptom list; the Post-COVID Functional Status (PCFS) Scale^27^; health-related quality of life measured with the EuroQol 5 Dimensions, 5 Levels (EQ-5D-5L) including its visual analogue scale^28^; severity of depressive symptoms measured with the eight-item Patient Health Questionnaire (PHQ-8)^29^; fatigue severity assessed with the Chalder Fatigue Scale^30^; pain intensity measured on a numeric rating scale; computer-based cognitive function testing with the Test of Attentional Performance (TAP; including alertness, divided attention, distractibility, visual scanning, and cognitive flexibility)^31^; physical fitness assessed using the 1-min Sit-to-stand test^32^; and use of on-demand medication. A feasibility and acceptability assessment served as an internal centre-specific process evaluation and was not designed for formal qualitative analysis. Owing to the large volume of data, the TAP outcomes could not be included in the present manuscript and will be reported separately.

Safety outcomes included the frequency and severity of AEs and SAEs, which were monitored continuously, reviewed at each scheduled visit and categorised according to the Medical Dictionary for Regulatory Activities (MedDRA) catalogue.

### Statistical analysis

For the study, a total sample size of 340 participants (85 per arm, accounting for a 15% dropout) was calculated to detect a clinically meaningful change (≥3 points) in the PROMIS total T-score,^33^ assuming no interaction between interventions, at 80% power and a significance level of 0·05. In February 2024, the sample size calculation was revised to assess whether the recruitment period could be shortened. This was possible because a review of retention based on blinded administrative data showed an observed attrition of 4%, lower than the assumed attrition of 15% used for the original sample size calculation. Without reference to outcome data and with all other design assumptions unchanged, the required total sample size was therefore revised to a minimum of 300 participants.

Data from the pilot and the confirmatory phase were analysed together. Baseline characteristics were evaluated descriptively. Pre-specified efficacy analysis (intention-to-treat) for the primary and secondary endpoints was carried out by general linear models (PCFS: ordinal logistic regression) with prednisolone and B vitamins as main factors, an interaction term for testing an interaction effect between prednisolone and B vitamins, and the respective baseline variable as covariate. Three null hypotheses were tested with corresponding F-tests within the models: no effect of vitamin B compound, no effect of prednisolone and no interaction effect between prednisolone and B vitamins. In case of a significant interaction effect, pairwise comparisons of the four treatment groups were planned. Otherwise the main effects are estimated by comparing the two arms with B vitamins versus the other two arms, and the two arms with prednisolone versus the other two arms, respectively.

Changes between day 0 and day 28 in primary and secondary endpoints for each of the four treatment groups separately were analysed by paired t-tests as post-hoc analyses. Numbers needed to treat (NNT) for clinical improvement in the primary and secondary endpoints were calculated together with a 95% confidence interval (post-hoc analysis). Changes in the PROMIS total score and its five domains at the follow-up visits on day 5, day 14, day 21, day 60, and day 180 were analysed post-hoc in the same way as for the primary endpoint on day 28.

Safety outcomes were reported descriptively, summarising the frequency and severity of AEs and SAEs for all randomised participants (safety population). All analyses adhered to International Council for Harmonisation (ICH) E9 guideline, Statistical Principles for Clinical Trials, using IBM SPSS Statistics (Version 29) and R Statistical Software version 4.5.1.

The success of blinding was assessed as pre-specified sensitivity analysis by calculating generalised Bang blinding indices per treatment arm.^34^ In case of 4 treatment arms, the indices can range between [−0·33, 1], with 1 representing complete unblinding, i.e. all participants have correctly guessed their treatment arm, and 0 representing perfect blinding success (either all participants answered with “don't know” or all guesses were completely random). The lower end of the range occurs if no participant guesses their correct treatment arm (“reverse unblinding”).

Detailed information on the study design, procedures, and planned analyses is available in the published study protocol^35^ and in the full trial protocol and statistical analysis plan in the Supplementary Material. A summary of all SAP versions is provided in eTable S8 of the Supplementary Appendix.

### Patient and public involvement

We established a collaboration with a citizens' forum, a group for patient and public involvement, at the Department of General Practice, University Hospital Würzburg, Germany. The study design, procedures, and all patient-facing materials were presented to the group on two occasions during the planning phase. Feedback from participants led to revisions of the study procedures, the information leaflet, and the patient diary to improve clarity and understandability. Patients were not involved in the statistical analysis or writing of the manuscript. Results will be disseminated to participants and the members of the citizens' forum upon publication.

### Ethics

This study was conducted in accordance with the Declaration of Helsinki and was approved by the Ethics Committee of the Medical Faculty of the University of Würzburg as the lead ethics committee (6 October 2022, reference no. 110/22_ff-am), the Ethics Committee of the Medical Faculty of the University of Tübingen (reference no. 450/2022AMG2), and the Ethics Committee of the Faculty of Medicine of Kiel University (reference no. B 265/22/RE). The study was authorised by the Federal Institute for Drugs and Medical Devices (BfArM, 14 October 2022, reference no. 61-3910-4045523). The trial was registered prospectively with ClinicalTrials.gov (NCT05638633) before the first participant was enrolled on January 13, 2023. All participants provided written informed consent before enrolment.

### Role of the funding source

The trial was funded by the German Federal Ministry of Research, Technology and Space (grant numbers for Würzburg, Tübingen and Kiel: 01EP2109A, 01EP2109B, and 01EP2109C) and the Vogel Stiftung Dr. Eckernkamp. Hevert-Arzneimittel GmbH & Co. KG supplied the B vitamins free of charge (Vitamin B Komplex forte Hevert). The funders and Hevert-Arzneimittel were not involved in the trial design, conduct, analysis, interpretation, writing the report, or in the decision to submit the paper for publication.

## Results

From 12 January 2023 to 25 July 2024, 2498 patients were assessed for eligibility, 326 gave informed consent and 323 underwent randomisation. 81 participants were assigned to the prednisolone group, 80 to the B vitamins group, 81 to the prednisolone plus B vitamins group and 81 to the placebo group (full analysis and safety analysis set). The main reason for ineligibility was recent use of the trial medications, with B vitamins being the most frequent cause of exclusion. Of those randomised, 315 took at least one dose of trial medication (intention-to-treat (ITT) population, Fig. 1). A total of 310 patients had evaluable data for the outcomes (Fig. 1).

**Fig. 1:**
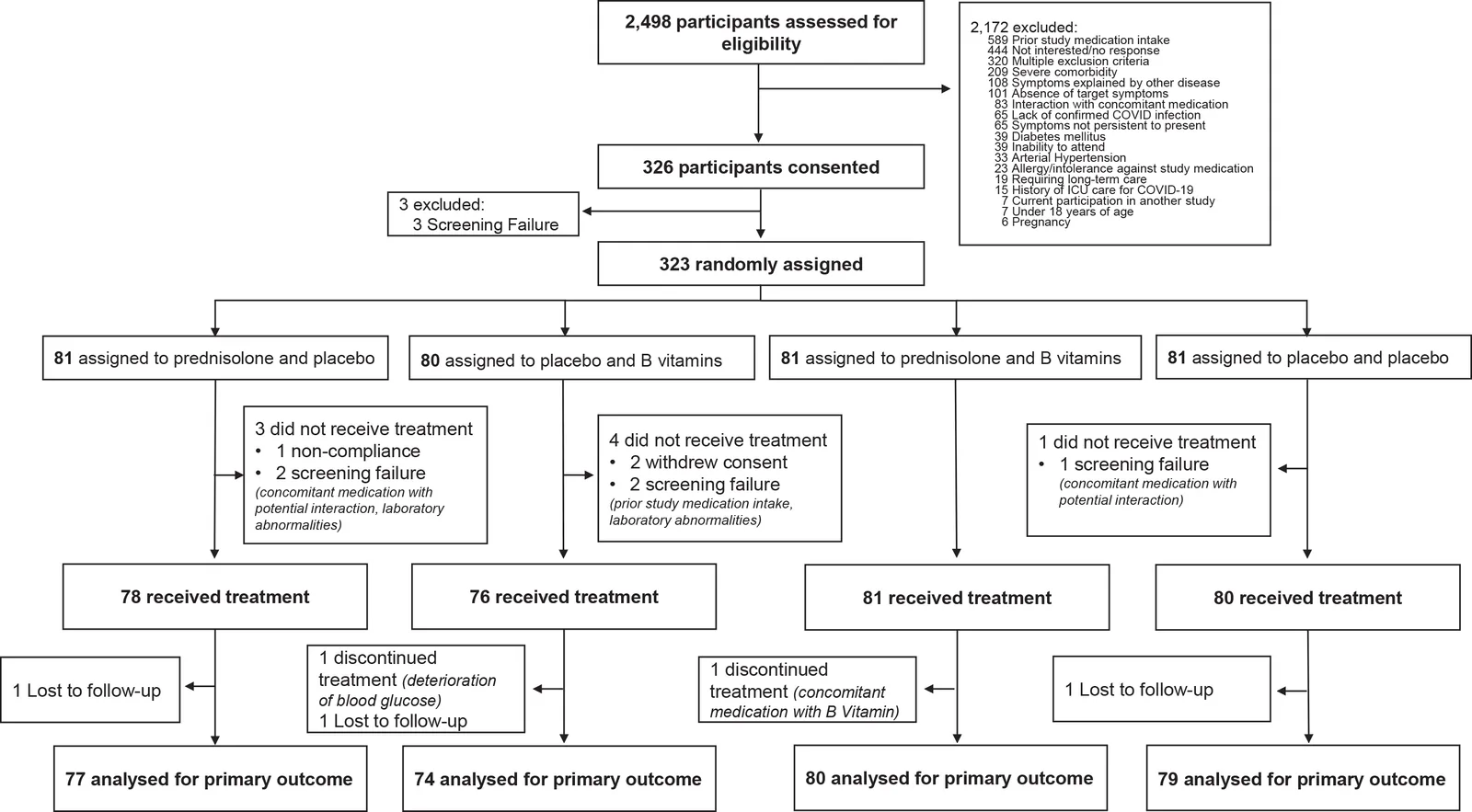
**Consolidated Standards of Reporting Trials (CONSORT) flow diagram of study participants**.

Baseline characteristics were similar among the four groups. The mean age of the ITT population was 44 (standard deviation [SD], 13) years, 200 (63%) were women, and 238 (76%) were in a partnership. Overall, 248 were employed and 131 (42%) worked full-time. Most infections occurred during the Omicron period (eFigure S2). Post-COVID symptoms had been present for a median duration of 79 weeks at enrolment and 307 (97%) participants were vaccinated against SARS-CoV-2 at least once, and 78% at least twice. Patients also reported having tried a mean of 2·8 (SD 1·9) different treatments for post-COVID before joining the trial (Table 1 and eTable S5).

**Table 1:** Baseline characteristics of participants by treatment group.

	Overall n = 315	Prednisolone n = 78	B vitamins n = 76	Prednisolone + B vitamins n = 81	Placebo n = 80
Age, mean (standard deviation (SD)), years	44 (13)	41 (13)	46 (12)	45 (12)	42 (13)
Sex					
Female	200 (63%)	47 (60%)	51 (67%)	51 (63%)	51 (64%)
Male	115 (37%)	31 (40%)	25 (33%)	30 (37%)	29 (36%)
Living with partner	238 (76%)	55 (71%)	57 (75%)	60 (74%)	66 (83%)
German as native language	310 (98%)	77 (99%)	76 (100%)	78 (96%)	79 (99%)
General higher education^a^	147 (47%)	35 (45%)	33 (43%)	38 (47%)	41 (51%)
Employed	248 (79%)	61 (78%)	65 (86%)	62 (77%)	60 (75%)
Of these: currently on sick leave	104 (42%)	25 (41%)	33 (51%)	24 (39%)	22 (37%)
Weeks on sick leave due to post-COVID, median (interquartile range [IQR])	24 (8, 46)	25 (6, 47)	24 (8, 42)	15 (6, 40)	32 (11, 52)
Duration of post-COVID symptoms, median (IQR), weeks	79 (51, 105)	82 (51, 102)	77 (51, 113)	72 (49, 111)	78 (55, 103)
No. of applied treatments for post-COVID, mean (SD)	2·81 (1·93)	2·78 (2·03)	2·79 (1·90)	2·77 (1·89)	2·90 (1·95)
Received at least 1 dose of COVID-19 vaccine	307 (97%)	77 (99%)	75 (99%)	76 (94%)	79 (99%)
Received ≥2 doses of vaccine before SARS-CoV-2 infection	246 (78%)	62 (79%)	58 (76%)	61 (75%)	65 (81%)
Smoking	33 (10%)	10 (13%)	9 (12%)	10 (12%)	4 (5·0%)
No. of comorbidities					
None	53 (17%)	11 (14%)	10 (13%)	16 (20%)	16 (20%)
One	66 (21%)	18 (23%)	14 (18%)	19 (23%)	15 (19%)
Two or more	196 (62%)	49 (63%)	52 (68%)	46 (57%)	49 (61%)
Comorbidities					
Body mass index ≥30	62 (20%)	14 (18%)	18 (24%)	18 (22%)	12 (15%)
Chronic respiratory diseases	88 (28%)	20 (26%)	19 (25%)	21 (26%)	28 (35%)
Cardiovascular diseases	88 (28%)	23 (29%)	20 (26%)	19 (23%)	26 (33%)
Psychiatric disorder	64 (20%)	22 (28%)	14 (18%)	12 (15%)	16 (20%)

a General or participant-specific higher education entrance qualification, equivalent to A-levels.

The symptom profile of the cohort at baseline is summarised in eTable S6. The mean change of symptom severity from baseline to day 28 in the PROMIS T-score was −4·3 (SD ± 5·1), p < 0·001 in the prednisolone group, −3·2 (±5·0), p < 0·001 in the B vitamins group, −4·5 (±4·5), p < 0·001 in the prednisolone plus B vitamins group, and −2·9 (±4·6), p < 0·001 in the placebo group. An interaction effect between both trial drugs was not detected (p = 0·93, Table 2).

**Table 2:** Primary and secondary outcomes and effect estimates of prednisolone and B vitamins (intention-to-treat population).

	Raw data in the four treatment arms: mean (standard deviation (SD))				Analysis of covariance		
	Prednisolone	B vitamins	Prednisolone + B vitamins	Placebo	Effect of prednisolone:	Effect of B vitamins:	Interaction
					mean [95% confidence interval (CI)]	mean [95% CI]	p-value
PROMIS^a^	(n = 77)	(n = 74)	(n = 80)	(n = 79)			
Day 0	36·3 (5·6)	37·6 (5·7)	36·9 (5·7)	36·5 (4·9)			
Day 28	32·0 (5·8)	34·4 (6·9)	32·4 (7·2)	33·6 (6·6)			
Change	–4·3 (5·1)	–3·2 (5·0)	–4·5 (4·5)	–2·9 (4·6)	–1·4 [–2·5 to –0·4]	–0·1 [–1·2 to +1·0]	
P-value	<0·001	<0·001	<0·001	<0·001	0·008	0·85	0·93
					(primary analyses)		
MYMOP	(n = 77)	(n = 74)	(n = 79)	(n = 79)			
Day 0	4·2 (0·7)	4·1 (0·8)	4·2 (0·8)	4·1 (0·7)			
Day 28	3·4 (1·2)	3·6 (1·1)	3·4 (1·3)	3·6 (1·0)			
Change	–0·8 (1·0)	–0·5 (1·0)	–0·8 (1·1)	–0·5 (0·9)	–0·3 [–0·5 to –0·1]	+0·0 [–0·2 to +0·3]	
P-value	<0·001	<0·001	<0·001	<0·001	0·004	0·69	0·98
EQ-5D-5L	(n = 77)	(n = 73)	(n = 80)	(n = 79)			
Day 0	0·72 (0·20)	0·68 (0·23)	0·69 (0·24)	0·69 (0·23)			
Day 28	0·82 (0·13)	0·71 (0·23)	0·77 (0·22)	0·72 (0·23)			
Change	+0·09 (0·20)	+0·03 (0·19)	+0·08 (0·18)	+0·03 (0·21)	+0·07 [+0·03 to +0·10]	–0·02 [–0·06 to +0·02]	
P-value	<0·001	0·10	<0·001	0·01	<0·001	0·35	0·57
PHQ-8	(n = 77)	(n = 74)	(n = 79)	(n = 79)			
Day 0	10·7 (4·1)	11·4 (4·5)	11·5 (4·4)	10·7 (4·2)			
Day 28	8·8 (3·9)	10·1 (4·9)	9·1 (4·1)	9·9 (4·9)			
Change	–1·8 (3·3)	–1·3 (3·2)	–2·4 (3·3)	–0·8 (2·9)	–1·1 [–1·8 to –0·4]	–0·4 [–1·1 to +0·3]	
P-value	<0·001	<0·001	<0·001	0·02	0·002	0·25	0·96
Fatigue	(n = 77)	(n = 74)	(n = 80)	(n = 79)			
Day 0	24·4 (5·0)	25·8 (5·8)	25·6 (5·3)	24·8 (4·6)			
Day 28	19·6 (7·3)	23·5 (7·2)	21·7 (7·2)	22·3 (6·2)			
Change	–4·8 (6·4)	–2·2 (5·1)	–3·9 (5·4)	–2·5 (5·0)	–2·0 [–3·2 to –0·8]	+0·8 [–0·5 to +2·0]	
P-value	<0·001	<0·001	<0·001	<0·001	0·001	0·22	0·61
Pain NRS	(n = 77)	(n = 73)	(n = 80)	(n = 79)			
Day 0	3·0 (2·4)	2·8 (2·3)	2·9 (2·4)	2·8 (2·2)			
Day 28	2·5 (2·4)	2·8 (2·4)	2·6 (2·6)	3·1 (2·4)			
Change	–0·5 (1·7)	+0·0 (1·9)	–0·3 (2·4)	+0·3 (2·1)	–0·5 [–0·9 to –0·1]	–0·1 [–0·5 to +0·4]	
P-value	0·01	0·88	0·26	0·16	0·02	0·76	0·29
Sit-to-Stand	(n = 77)	(n = 71)	(n = 73)	(n = 74)			
Day 0	26·0 (8·9)	22·8 (7·7)	25·5 (9·8)	24·8 (9·0)			
Day 28	29·1 (10·7)	24·2 (8·4)	28·1 (11·2)	25·5 (10·2)			
Change	+3·1 (6·6)	+1·4 (5·1)	+2·6 (4·8)	+0·7 (5·5)	+1·8 [+0·6 to +3·1]	+0·1 [–1·2 to +1·3]	
P-value	<0·001	0·02	<0·001	0·29	0·005	0·93	0·38

	Raw data in the four treatment arms: mean (standard deviation (SD))				Ordinal logistic regression		
	Prednisolone	B vitamins	Prednisolone + B vitamins	Placebo	Effect of prednisolone:	Effect of B vitamins:	Interaction
					odds ratio (OR) [95% CI]	OR [95% CI]	p-value
PCFS	(n = 77)	(n = 74)	(n = 80)	(n = 79)			
Day 0	2·5 (0·7)	2·5 (0·7)	2·6 (0·7)	2·7 (0·6)			
Day 28	2·3 (0·7)	2·4 (0·8)	2·2 (0·9)	2·5 (0·7)			
Change	–0·2 (0·6)	–0·2 (0·7)	–0·4 (0·7)	–0·2 (0·6)	0·53 [0·33–0·84]	0·70 [0·44–1·12]	
P-value	0·01	0·04	<0·001	0·01	0·007	0·14	0·22

a The primary outcome was change in overall symptom severity from baseline to day 28, assessed by the Patient-Reported Outcomes Measurement Information System (PROMIS) five-component overall score; Secondary outcomes were changes at the respective scores: Measure Yourself Medical Outcome Profile (MYMOP®): burden of the two subjectively most important symptoms; EQ-5D-5L: limitations in daily activities (EuroQoL, 5 dimensions, 5 levels); Patient Health Questionnaire-8 (PHQ-8): sum score of 8-item depression questionnaire; Fatigue: Chalder Fatigue Scale; Pain NRS: 10-point numeric rating scale of pain; Sit-to-Stand: number of stand-ups from sitting in 1 min; PCFS: 5-point, categorical post-COVID functional status scale (0–4); Changes from day 0 to day 28 are presented as means and standard deviations. p-values in the left side of the table are from paired t-tests. Effect estimates are the estimates of the mean effect of the respective drug on the outcome. p-values in the right side of the table are from ANCOVA.

The unadjusted mean difference of symptom severity between patients treated with B vitamins and patients without was −0·2 (95% confidence interval (CI) −1·3 to +0·8), p = 0·68. Adjusted for the baseline value, the mean difference was −0·1 (95% CI –1·2 to +1·0), p = 0·85 between baseline and day 28.

At day 28, the unadjusted mean difference in the PROMIS T-score between participants treated with prednisolone and those not treated was −1·4 (95% confidence interval [CI] −2·4 to −0·3), p = 0·012. The mean difference adjusted for the baseline value was −1·4 (95% CI, −2·5 to −0·4), p = 0·008.

The number needed to treat (NNT) with prednisolone for a clinically meaningful change in the PROMIS T-score was 7·5 (95% CI 4·0–55·1, eTable S1). During follow-up, the differences between the groups measured with the PROMIS decreased in all groups and were not significant at days 60 and 180. However, an overall effect of the intervention on PROMIS scores was observed across all four groups, including at 60 and 180 days (Fig. 2). The observed PROMIS effect appeared to be driven primarily by the change in fatigue subscore, a prespecified secondary outcome (−6·4 points, p < 0·001) (eFigure S1). At day 28, all secondary outcomes were statistically significantly improved in the prednisolone group, including patient-reported symptoms as measured by MYMOP, EQ-5D-5L, PHQ-8, Chalder Fatigue Scale, numeric rating scale for pain, Sit-to-stand test, and PCFS (Table 2).

**Fig. 2:**
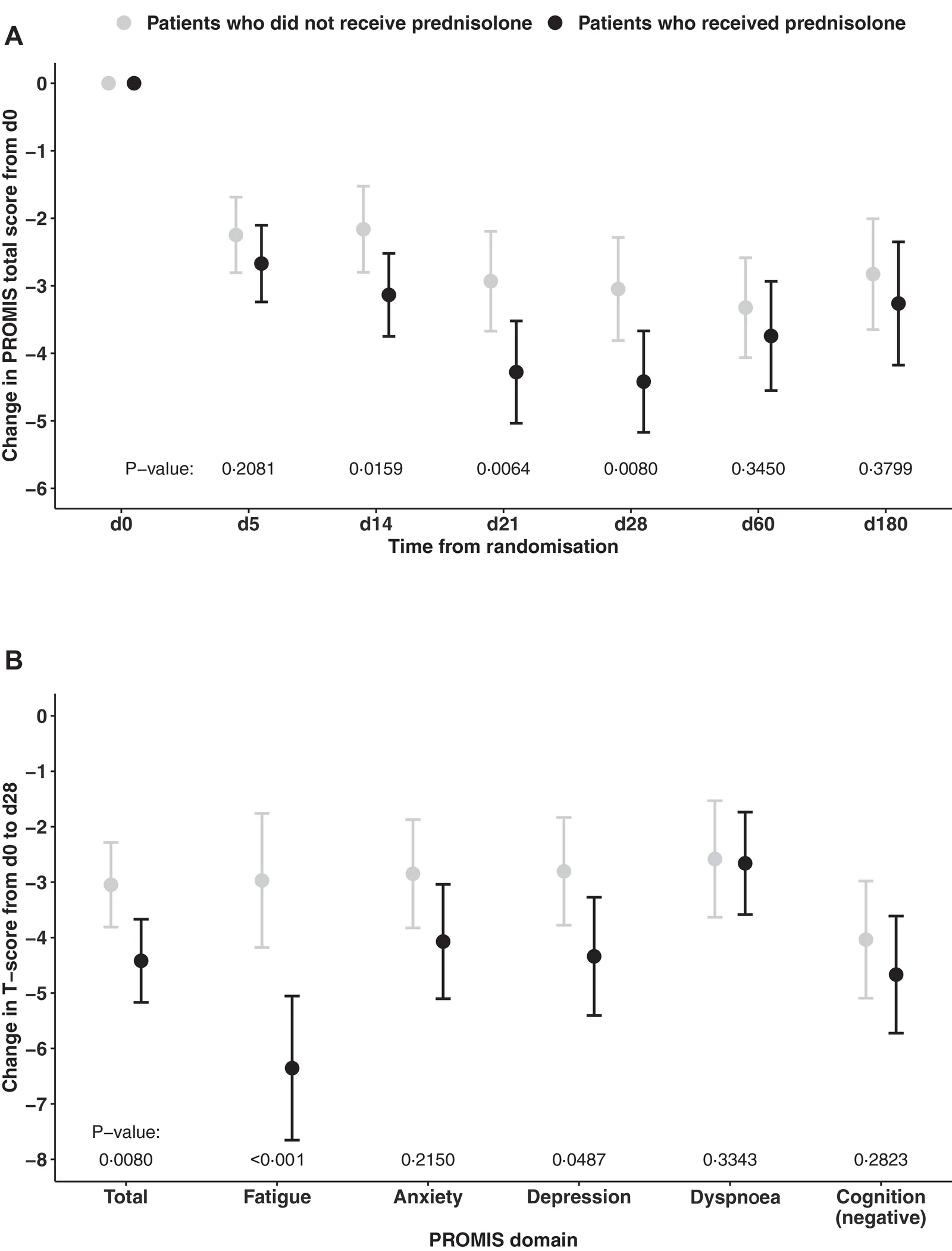
**Changes in Patient-Reported Outcomes Measurement Information System (PROMIS) total score and in T-scores of the PROMIS domains.** Data are means and their 95% confidence interval (CI) in the groups of patients who did or did not receive prednisolone (regardless of B vitamins administration). p-values are from ANCOVA and refer to the null hypotheses of no between-group difference (adjusted for the respective baseline values). Panel A: Changes of PROMIS total score from baseline (day 0) to each of the scheduled study visits during and after treatment (up to day 180). The treatment effect is recognisable after two weeks, reaches its full extent after four weeks (at the end of treatment), and becomes slightly smaller thereafter—regardless of group assignment. Statistically significant differences between patients receiving prednisolone and those not are observed only on days 14, 21 and 28, i.e. during treatment. Panel B: Changes from day 0 to day 28 in PROMIS total score and its five domains. For greater comprehensibility, values of the cognition subscale are reversed, therefore low values indicate lower levels of burden in each of the subdomains shown. Domain sum scores were translated to T-scores using large population reference data, and therefore are comparable both to each other and to the overall average score (PROMIS total score).

The most frequently reported symptom in MYMOP was fatigue (58% of participants), followed by concentration difficulties (27%), pain (21%), and cognitive impairment (20%). Improvement was predominantly observed in fatigue, where more patients treated with prednisolone reported a decrease in severity by ≥1 point than those not treated (31% vs. 23%, eTable S2). Co-medication remained largely unchanged during the intervention period (eTable S3).

Adverse events were equally distributed between groups (Table 3). Seven serious adverse events (SAE) were reported by day 60 while trial drugs were pharmacologically active: three in the prednisolone group, two in the B vitamins group, and one in each of the other groups. Twelve further SAEs were reported between days 61 and 180. All SAEs were classified as ‘unrelated’ or ‘unlikely to be related’ to the study medication and all but one SAE of breast cancer were resolved at the end of the follow-up period (Table 3). No fatalities occurred during the conduct of the trial. In particular, no new safety concerns related to the administered medication emerged.

**Table 3:** Adverse events and serious adverse events from baseline to day 60 (safety population).

	Overall n = 323	Prednisolone n = 81	B vitamins n = 80	Prednisolone + B vitamins n = 81	Placebo n = 81
Adverse Events (AE)					
Total number of AEs	820	219	176	237	188
Participants with one or more AEs	269 (83%)	67 (83%)	62 (78%)	70 (86%)	70 (86%)
Maximum severity					
Mild	162 (50·2%)	39 (48·1%)	39 (48·8%)	49 (60·5%)	35 (43·2%)
Moderate	95 (29·4%)	26 (32·1%)	18 (22·5%)	19 (23·5%)	32 (39·5%)
Severe/life-threatening	12 (3·7%)	2 (2·5%)	5 (6·2%)	2 (2·5%)	3 (3·7%)
Participants with one or more study-drug related^a^ AEs	138 (42·7%)	37 (45·7%)	29 (36·2%)	40 (49·4%)	32 (39·5%)
Selected AEs consistent with the known safety profile of prednisolone					
Sleep disorder	9 (2·8%)	3 (3·7%)	3 (3·7%)	3 (3·7%)	0
Hypertension	7 (2·2%)	0	5 (6·2%)	1 (1·2%)	1 (1·2%)
Hyperglycaemia^b^	1 (0·3%)	0	0	1 (1·2%)	0
Infection susceptibility	1 (0·3%)	0	0	1 (1·2%)	0
Mood swings	1 (0·3%)	0	1 (1·2%)	0	0
Participants with AEs resulting in withdrawal or interruption of study drug	8 (2·5%)	1 (1·2%)	2 (2·5%)	3 (3·7%)	2 (2·5%)
Serious Adverse Events (SAE)					
Total number of SAEs	7	3	2	1	1
Participants with one or more SAEs	7 (2·2%)	3 (3·7%)	2 (2·5%)	1 (1·2%)	1 (1·2%)
Maximum severity					
Moderate	1 (0·3%)	1 (1·2%)	0	0	0
Severe/life-threatening	6 (1·9%)	2 (2·5%)	2 (2·5%)	1 (1·2%)	1 (1·2%)
Participants with one or more study-drug related^a^ SAEs	0	0	0	0	0
Participants with SAEs resulting in withdrawal or interruption of study drug	0	0	0	0	0

a Study-drug related: possibly, probably or related to study medication.

b Mean non-fasting blood glucose was 106 mg/dl in the prednisolone group (maximum 174 mg/dl) and 106 mg/dl in the non-prednisolone group (maximum 173 mg/dl).

On day 180, 26% of the study participants correctly guessed their assignment to the treatment group, while 24% had no idea. The generalised Bang blinding indices calculated for each treatment arm were 0·22 for prednisolone, −0·03 for B vitamins, −0·07 for prednisolone plus B vitamins, and 0·25 for placebo. The study team had no idea about the group assignment in 42% of all participants, in 20% the team's assumption was correct. The generalised Bang blinding indices for the study team's assumption were 0·06 for prednisolone, 0·06 for B vitamins, 0·08 for prednisolone plus B vitamins, and 0·12 for placebo (eTable S4).

## Discussion

This randomised, double-blind, placebo-controlled phase 3 factorial trial showed that a 28-day course of prednisolone led to a statistically significant reduction in post-COVID symptom severity compared with no prednisolone as shown in the five dimensions of the PROMIS score. However, the between-group difference did not reach the predefined threshold of ≥3 points in the PROMIS T-score considered clinically meaningful. This distinction is important, as statistical significance alone does not establish clinical relevance.

Post-hoc analyses indicate that the effect on the PROMIS T-score was driven primarily by improvements in the fatigue subscore, the most dominant symptom in the trial population. However, fatigue itself was not the primary outcome and the clinical importance of these fatigue-specific changes warrants cautious interpretation. On an individual level, the NNT was 7·5 for one additional patient to achieve a clinically meaningful improvement or avoid relevant deterioration on the PROMIS T-score. The wide confidence interval around the NNT (4·0–55·1) reflects the statistical uncertainty in the underlying absolute risk difference and should be taken into account when interpreting the clinical relevance of this finding.

Presumably due to its prevalence in patients with post-COVID, Reis et al.^36^ chose fatigue as the primary endpoint in a recent adaptive platform RCT. The authors showed a moderate improvement in patients with post-COVID after 60 days of fluvoxamine treatment.

Participants in all groups showed an improvement in symptom severity from baseline to day 28, with the greatest changes in the PROMIS T-score observed in the prednisolone group. Treatment with vitamins B1, B6, and B12, as well as placebo, resulted in smaller, but still statistically significant improvements. The statistically significant placebo effect might be one of the reasons why the predefined between-group difference of ≥3 points in the PROMIS T-score was not reached.

The improvement in fatigue may relate not only to general anti-inflammatory effects but also to hypothalamic-pituitary-adrenal axis dysfunction, a mechanism increasingly implicated in post-COVID condition and previously described in myalgic encephalomyelitis/chronic fatigue syndrome.^37^^,^^38^

Until this study, evidence for corticosteroids in post-COVID was confined to patients with organising pneumonia or persistent inflammatory lung disease rather than the broader spectrum of post-COVID symptoms. Current evidence likewise does not support corticosteroid use in post-viral chronic fatigue syndromes due to safety concerns and the lack of data on long-term effects in previous trials.^39^^,^^40^ Systematic reviews therefore caution against routine corticosteroid treatment for the wider range of post-COVID symptoms because of unproven benefit and potential harm, recommending use only for specific pulmonary indications.^41^

Our findings suggest that improvement in the B vitamins group may be more likely due to a placebo effect than to the vitamins themselves, possibly validated by participating in the trial and feeling taken seriously.

Placebo responses are pronounced in conditions with subjective symptoms such as fatigue, pain, and cognitive impairment, all common in post-COVID. A systematic review of post-COVID interventions likewise found that placebo-controlled trials frequently report symptom improvement in both arms.^8^ In post-COVID trials, these placebo effects typically persist only for the duration of the intervention, without sustained or progressive improvement thereafter, a pattern consistent with other chronic conditions such as myalgic encephalomyelitis/chronic fatigue syndrome, fibromyalgia, and chronic pain syndromes.^42^^,^^43^ In our study, the effect measured by the PROMIS T-score was still evident in all groups one and five months after the intervention ended. This underscores the importance of maintaining ongoing, respectful contact with those affected. In a recent RCT, however, both the nirmatrelvir–ritonavir and placebo–ritonavir groups showed only minor changes in PROMIS T-scores compared with baseline, with no significant between-group difference, indicating no substantial placebo effect.^6^ For selected patients, prednisolone might offer an inexpensive, symptomatic treatment option, but clinicians should balance short-term benefits against potential long-term harms such as hyperglycaemia, hypertension, mood changes and the risk of steroid-induced diseases, which may complicate long-term management and outweigh initial improvements.

The substantial improvement observed in the placebo group may reflect several overlapping mechanisms. Post-COVID symptoms are known to fluctuate naturally over time, and given that patients were enrolled after a median symptom duration of 79 weeks, some degree of spontaneous improvement or regression to the mean is plausible, particularly as participants may have entered the trial during a period of relatively higher symptom burden. A genuine placebo effect and non-specific effects of trial participation, including increased attention, symptom monitoring, and supportive contact with the study team, may also have contributed. In addition, some participants appeared to regard their participation as an opportunity to contribute both to research and to greater recognition of post-COVID as a serious condition. This sense of purpose and societal contribution may have positively influenced their well-being, too, although it was not systematically assessed. Against this background, the incremental benefit of prednisolone beyond placebo was modest and did not reach the predefined threshold for clinical meaningfulness.

This double-blind, randomised controlled trial with a factorial design employed a variety of treatment regimens. It was conducted in a real-world outpatient setting, and showed a low dropout rate of 4% among randomised participants. A limitation is the choice of outcome instruments: in the absence of an established core outcome set for post-COVID trials, we relied on standard measures for endpoint assessment. Symptom recording was largely subjective because objective measures and measures of mechanistic correlates (e.g. viral persistence, inflammatory mediation, or pharmacokinetics) are lacking. Generalisability may be limited to symptoms defined by the inclusion criteria, the safety-related exclusion criteria, particularly diabetes mellitus and severe comorbidities, and the exclusion of patients requiring nursing care or who were bedridden. Another limitation is that Long COVID phenotypes such as orthostatic intolerance or postural orthostatic tachycardia syndrome were neither used as inclusion criteria nor explicitly assessed. Data from the Test for Attentional Performance, although prespecified as a secondary outcome, are not reported in the present manuscript and will be reported separately. The qualitative feasibility and acceptability assessment is also not presented, as it was considered an internal process evaluation rather than a formal study outcome. While the amendments to the eligibility criteria during the recruitment phase may have increased heterogeneity in terms of comorbidities and concomitant medication use, the broader eligibility criteria may also have increased the study's external validity.

PreVitaCOV is, to our best knowledge, the first trial showing a short-term statistically significant effect of prednisolone on post-COVID symptoms, of uncertain clinical magnitude. Further studies are needed to confirm these findings in larger, more diverse populations and to clarify optimal dosing and treatment duration, since the benefit disappeared after stopping the medication. Investigations using biomarker profiling might help delineate responders from non-responders and clarify the biological mechanisms underlying treatment response. Long-term follow-up, which is ongoing, will be essential to assess the durability of effect and safety beyond the 28-day course.

In conclusion, prednisolone showed a statistically significant, but modest improvement in fatigue that did not meet prespecified criteria for clinical meaningfulness. Decisions regarding its use as a potential therapeutic option in patients with post-COVID condition should balance the known side effect profile against this possible symptomatic benefit, pending further research to confirm this finding, identify likely responders, and clarify optimal dosing and duration.

## Contributors

IG, HK, SJ, and JD acquired funding for the trial. IG, HK, SJ, CFö, WM, KGH, YK, and OZ contributed to the conceptualisation and design of the study. AK was responsible for project administration. HK, AK, CFö, AS, CE, JD, LL, and CK were responsible for the investigation and patient inclusion. GG and CFi accessed and verified the underlying data and contributed to data curation, formal analysis, and methodology. IG, HK, AK, and AS wrote the first draft of the manuscript. All other authors critically revised the manuscript for important intellectual content. All authors approved the final version of the manuscript. IG acted as sponsor of this investigator-initiated trial and is the guarantor. All authors had full access to all of the data (including statistical reports and tables) in the study and can take responsibility for the integrity of the data and the accuracy of the data analysis. The corresponding author attests that all listed authors meet authorship criteria and that no others meeting the criteria have been omitted.

## Data sharing statement

The data and source code that support the findings of this study will be made available to qualified researchers on reasonable request. Proposals should be directed to IG (Gagyor_I@ukw.de). Data will be available after publication, following approval by the study steering committee. The study protocol and statistical analysis plan are available in the Supplementary Material.

## Declaration of interests

KGH reports consulting fees from AstraZeneca, Bayer, and Pfizer, and payment or honoraria for lectures, presentations, speakers bureaus, manuscript writing or educational events from AstraZeneca, BMS, Daiichi Sankyo, Boehringer Ingelheim, Pfizer, Novartis, Alexion, Boston Scientific, the University of Bern, the University of Leipzig, and the University of Jena. GG reports payment for expert testimony as a member of the Ethics Committee of the Medical Faculty Würzburg for monthly sessions and participation on a Data Safety Monitoring Board or Advisory Board Member of Steering Committee in FIND-AF-II trial, a trial on re-stroke prevention (payment of travel expenses). LL reports support for attending a conference (DEGAM, German conference). All other authors declare no competing interests.

## Appendix A Supplementary data

Supplementary Figures and Tables

Supplementary Study Protocol

Supplementary SAP
