# Supplementary material for: Low-dose prednisolone and B vitamins for post-COVID-19 syndrome in Germany: the PreVitaCOV randomised clinical trial
Source: eClinicalMedicine. 2026 Oct 24;101:104264. doi: 10.1016/j.eclinm.2026.104264 (PMC13720163; doi:10.1016/j.eclinm.2026.104264)
Supplement: Supplementary file 1 [file mmc2.pdf]

TRIAL PROTOCOL

PreVitaCOV

Prednisolone and vitamin B1, B6, and B12 in patients with Post-COVID-19-Syndrome (PC19S) – a randomized controlled trial in primary care

Short name: PreVitaCOV

Objectives: to assess the effectiveness, safety and feasibility of treating patients with PC19S in primary care with prednisolone and/or vitamin B1, B6, and B12 in a fixed combination.

Design: double blind, randomized controlled trial, factorial design, four parallel groups, placebo-controlled, phase IIIb

Version: 5.0, 28.08.2023

Authors: C. Förster, Y. Kaußner, C. Tengelmann, U. Malzahn, L. Lunden, C. Eidenschink, K. Erdmann, E. Feil, S. Sellmann, G. Borgulya, K. Häusler, S. Joos, H. Kaduszkiewicz, K. Hügen, K. Hartung, O. Zolk, P.U. Heuschmann, I. Gágyor

Sponsor:
University Hospital Würzburg, Germany; Josef-Schneider-Str. 2/D7, 97080 Würzburg

PCI:
Ildikó Gágyor, Prof. Dr. med.; University Hospital Würzburg, Dept. of General Practice; UKW; Josef-Schneider-Str. 2/D7, 97080 Würzburg; +49 (0)931-201-47802; Gagyor_I@ukw.de

PI:
Stefanie Joos, Prof. Dr. med.; University Hospital Tübingen, Institute of General Practice and Interprofessional Care; Osianderstr. 5, 72076 Tübingen; +49 (0)7071 29 85213; Stefanie.joos@med.uni-tuebingen.de

Walter Maetzler, Prof. Dr. med.; University of Kiel, Institute of Neurology; Arnold-Heller-Str. 3, 24105 Kiel; +49 (0)431 500 23981; w.maetzler@neurologie.uni-kiel.de

Collaborating Sites:
Prof. Dr. med. Peter Heuschmann, Klemens Hügen, Dr. U. Malzahn; University Hospital Würzburg, Clinical Trial Center, ; Josef-Schneider Str. 2, 97080 Würzburg; zks_studien@ukw.de

Catharina Escales, Dr. med., University of Kiel, Institute of General Practice, Michaelisstr. 5, 24105 Kiel, +49 (0) 431 500 30101, +49 (0) 431 500 30104, escales@allgemeinmedizin.uni-kiel.de

Karl Georg Häusler, Prof. Dr. med., University Hospital Würzburg, Dept. of Neurology, Josef-Schneider-Str. 2/B1, 97080 Würzburg, +49 (0)931 201 23755, +49 (0)931 201 23946, haeusler_k@ukw.de,

Oliver Zolk, Prof. Dr. Med.; Medizinische Hochschule Brandenburg CAMPUS GmbH; Fehrbelliner Straße 38, 16816 Neuruppin;
+49 (0) 33638 83-991; Oliver.Zolk@mhb-fontane.de

Ethics Committee:
Ethik-Kommission der Medizinischen Fakultät; University Hospital Würzburg; Josef-Schneider-Str. 4/C12, 97080 Würzburg

Protocol code of the Sponsor: F001AM0222_1

EudraCT number: 2022-001041-20

All information in this trial protocol is to be kept strictly confidential. It is intended for the information of the participating investigators, other participating persons, the ethics committees and the authorities only.

This trial protocol may not be passed on to third parties without the permission of the Coordinating Investigator, Prof. Dr. med. Ildikó Gágyor.

Contents

1 Abbreviations ... 8

2 Participating persons/ institutes ... 10

3 Synopsis ... 13

4 Synopse ... 18

5 Visit plan ... 24

6 Introduction ... 25

6.1 Background of the clinical trial ... 25
6.2 Rationale for the trial ... 27
6.3 Assessment of risks and benefits ... 29
6.4 Patient involvement ... 32

7 Trial objectives ... 34

7.1 Primary, secondary and safety endpoints ... 34

8 Description of the trial ... 35

8.1 Overall trial design ... 35
8.2 Flow Chart ... 35
8.3 Primary outcome measures ... 35
8.4 Secondary outcome measures ... 36
8.5 Validation of the outcomes for the elderly ... 37
8.6 Sample size ... 38
8.7 Strategy for transition from pilot study to confirmatory study ... 39
8.8 Timetable ... 40

9 Trial population ... 41

9.1 Inclusion criteria ... 41
9.2 Exclusion criteria ... 41
9.3 Patient recruitment ... 42

10 Trial medication ... 44

10.1 Trial drug 1: Prednisolone ... 44
10.2 Trial drug 2: Vitamin B compound ... 44
10.3 Placebo for Prednisolone ... 44
10.4 Placebo for Vitamin B compound ... 45
10.5 Packaging and labeling ... 45

10.6 Storage, release and return and documentation of the trial product (drug accountability) 46
10.7 Randomization 46
10.8 Treatment schedule 47
10.9 Administration instructions 47
10.10 Emergency procedures 47
10.11 Concomitant medication 48
10.12 Compliance 48
10.13 Blinding and sealed opaque envelopes 49
10.13.1 Blinding 49
10.13.2 Early unblinding 49
10.13.3 Regular unblinding 50

11 Trial procedures 51

11.1 Prescreening 51
11.2 Visit 1 (baseline, day 0) 51
11.3 Phone call 1 (day 5+3) 52
11.4 Patient diary (day 1 to 28) 53
11.5 Visit 2 (day 28+3) 53
11.6 Phone call 2 (day 60+/-5) 55
11.7 Phone call 3 (day 180+/-7) 55
11.8 Assessment of safety 55
11.9 Biosamples (Laboratory tests) 56
11.10 Sub study *Patients' perspectives and needs concerning care of Post-COVID-19* 58
11.10.1 Operationalization of interviews 58
11.10.2 Selection of the sample 58
11.10.3 Benefits and risks 58
11.10.4 Analysis 58
11.11 Continued treatment of patients after the end of the trial 59
11.12 Pregnancy 59
11.12.1 Documentation of pregnancies 60
11.12.2 Documentation and follow-up of pregnancy complications and miscarriage 61
11.12.3 Documentation storage 61
11.13 Patient withdrawals (Drop-outs) 61
11.13.1 Discontinuation of (single) subjects 61
11.14 Early termination of the trial 62

12 Adverse events, adverse reactions 63

12.1 Definitions 63
12.2 Documentation of AEs 64
12.3 Documentation of SAEs 64

12.4 Reporting of AEs and ARs 65
12.4.1 Notification duties of the Investigator (§ 13 (1) – (6) GCP-V pursuant to § 42 AMG) ...65
12.4.2 Notification duties of the Sponsor (§ 13 (1) – (6) GCP-V pursuant to § 42 AMG)....65

13 Documentation....67
13.1 Electronic case report form (eCRF) 67
13.2 Investigator site file 67
13.3 Data archiving 68
13.3.1 Archiving duties of the Sponsor 68
13.3.2 Archiving duties of the investigator 68

14 Quality Control and Quality Assurance 69
14.1 Monitoring 69
14.2 Auditing 70

15 Data entry and data management 71
15.1 Data Collection and Data Correction 71
15.1.1 Electronic Data Capture System....71
15.2 Source Documentation....71
15.3 Database and Data Management....71
15.4 Confidentiality of Study Data and Data Protection 72
15.5 Archiving 73

16 Statistics....74
16.1 Sample size planning 74
16.2 Placebo as a comparator 75
16.3 Randomization 76
16.4 Statistical methods 76
16.4.1 Outcome measures....76
16.4.2 Definition of analysis populations 77
16.4.3 Data analysis 77
16.3.4 Blinding assessment and interpreting data from questionnaires in the case of imperfect blinding 79

17 Reporting....82
17.1 Trial report....82
17.2 Publications....82

18 Ethical, legal and administrative aspects....83
18.1 Responsibilities of the Sponsor and investigator 83
18.2 Ethics committee opinion and notification of the authorities....83
18.3 Patient information and informed consent 83
18.4 Patient insurance 84

18.5 Data protection and secrecy 84

19 Protocol amendments 86

20 References 87

21 Signatures 90

1 Abbreviations

**ANOVA**	Analysis of variance
**AE**	Adverse event
**AIDS**	Acquired Immune deficiency Syndrome
**AMG**	Arzneimittelgesetz (German Medicines Act)
**BayFoNet**	Das Bayerische Forschungsnetz in der Allgemeinmedizin (Bavarian Research Network in General Medicine)
BfArM	Bundesinstitut für Arzneimittel und Medizinprodukte **(**Federal Institute for Drugs and Medical Devices)
**COPD**	Chronic Obstructive Pulmonary Disease
**COVID**	Coronavirus Disease
**CRO**	Contract Research Organisation
**CTCW**	Clinical Trial Center Würzburg
**DEGAM**	Deutsche Gesellschaft für Allgemeinmedizin und Familienmedizin
	(German College of General Practitioners and Family Physicians)
**DESAM-ForNet**	Initiative Deutscher Forschungspraxennetze
	(The Initiative of German Research Practice Network)
**DSGVO**	Datenschutzgrundverordnung (German federal Data Protection Law)
**DSMB**	Data Safety Monitoring Board
**eCRF**	Electronic case report form
**EQ-5D**	European Quality of Life 5 Dimensions
**EQ-VAS**	European Quality of Life visual analogue scale
**FoPraNet-BW**	Forschungsnetzwerk in Baden-Württemberg (Research Network in Baden-Württemberg)
**FOSA**	Fach- und Organspezifischen Arbeitsgruppen (Subject- and organ-specific working groups)
**Gamma GT**	Gamma-Glutamyltransferase
**GCP**	Good Clinical Practice
**GCP-V**	GCP-Verordnung (GCP-Regulation)
**GM-CSF**	Recombinant Human Granulocyte Macrophage Colony-Stimulating-Factor
**GOT**	Glutamat-Oxalacetat-Transaminase
**GP**	General Practitioner
**GPT**	Glutamat-Pyruvat-Transaminase
**HMSS**	Health Measuring Scoring Service
**ICH**	International Council for Harmonization
**ID**	Identity document
**IMP**	Investigational Medicinal Product
**IMPD**	Investigational medicinal product dossier

**IIT**	Investigator-initiated trial
**ITT**	Intention to treat
**MoCA**	Montreal Cognitive Assessment
**MYMOB**	Measure Yourself Medical Outcome Profile
**NSAID**	Nonsteroidal Anti-inflammatory Drugs
**NAPKON**	Nationales Pandemie Kohorten Netz (German National Pandemic Cohort Network)
**NRS**	Numeric Rating Scale
**NUM**	Netzwerk Universitätsmedizin (Network University Medicine)
**NUM NUKLEUS**	NUM Klinische Epidemiologie und Studienplattform (Network University Medicine Clinical Epidemiology and Study Platform)
**ECU**	Epidemiology Cor Unit
**NYHA**	New York Heart Association
**PC19S**	Post-COVID-19-Syndrome
**PCFS**	Post-COVID-19 Functional Status Scale
**PCI**	Principal coordinating investigator
**PCR**	Polymerase chain reaction
**PHQ8**	Eight-item Patient Health Questionnaire depression scale
**PI**	Principal Investigator
**PP**	Per protocol
**PROMIS**	Patient Reported Outcomes Measurement Information System
**RCT**	Randomized controlled trial
**SAE**	Serious adverse event
**SAR**	Serious adverse reaction
**SARS**	Severe acute respiratory syndrome
**SD**	Standard deviation
**SM**	Standard deviation of means
**SmPC**	Summary of product characteristics
**SOP**	Standard operating procedure
**SUSAR**	Suspected unexpected serious adverse reaction
**TAP**	Testbatterie zur Aufmerksamkeitsprüfung (Attention test battery)
**TNF-α**	Tumor necrosis factor Alpha
**TSC**	Trial Steering Committee
**TSH**	Thyroid-stimulating-hormone
**UADR**	Unexpected adverse drug reaction

2 Participating persons/ institutes

**Sponsor:**	University Hospital Würzburg, Germany;<br>Josef-Schneider-Str. 2/D7, 97080 Würzburg
**Delegate of Sponsor and coordinating Investigator:**	Ildikó Gágyor, Prof. Dr. med.<br>University Hospital Würzburg, Germany, Dept. of General Practice; UKW; Josef-Schneider-Str. 2/D7, 97080 Würzburg; (0049)931-201-47802; Gagyor_I@ukw.de<br>Tel.: +49 (0) 931-201-47802<br>Fax: +49 (0) 931 201-647806<br>E-mail: gagyor_i@ukw.de
**Study Centers:**	Ildikó Gágyor, Prof. Dr. med.<br>University Hospital Würzburg, Dept. of General Practice; Josef-Schneider-Str. 2/D7, 97080 Würzburg; Tel.: +49 (0) 931-201-47802<br>Fax: +49 (0) 931 201-647806<br>E-mail: allgemeinmedizin@ukw.de<br><br>Stefanie Joos, Prof. Dr. med.<br>University Hospital Tübingen, Institute for General Practice and Interprofessional Care<br>Osianderstrasse 5, 72076 Tübingen<br>Tel.: +49 (0) 7071 29-85213<br>Fax: +49 (0) 7071 29-5896<br>E-mail: sekretariat.allgemeinmedizin@med.uni-tuebingen.de<br><br>Study Center Kiel:<br>Hanna Kaduszkiewicz, Prof. Dr. med.<br>University of Kiel, Institute of General Practice<br>Michaelisstr. 5, 24105 Kiel<br>Tel.: +49 (0) 431 500 30101<br>Fax.: +49 (0) 431 500 30104<br>E-Mail: office@allgemeinmedizin.uni-kiel.de<br><br>PI of the clinical trial study center Kiel:<br>Walter Maetzler, Prof. Dr. med.<br>University of Kiel, Institute of Neurology;Arnold-Heller-Str. 3, 24105 Kiel;<br>Tel.: +49 (0)431 500 23981;<br>Fax: +49 (0)431 500 23848<br>w.maetzler@neurologie.uni-kiel.de

Structure and Governance:

The Trial Steering Committee (TSC) will consist of the Coordinator and PI of the trial site Würzburg (IG) and the PIs of the other trial sites (SJ, WM). It will meet via video conference every four weeks during the study to ensure that study procedures are harmonised at each center, recruitment targets achieved, deadlines set and the agreements recorded. The independent Data Safety and Monitoring Board (DSMB) will ensure safety and data quality by considering adherence to protocol and monitoring reports including amount and seriousness of findings. It will advise whether to continue, modify or stop the trial, reviewing Adverse Events (AEs), Serious Adverse Events (SAEs) and Suspected Unexpected Serious Adverse Reaction SUSARs. The committee comprises independent board members including a statistician, a general practitioner (GP), a neurologist and a clinical pharmacologist. The board is planned to meet after 50% patient inclusion. The DSMB will report to the Sponsor and coordinating investigator as appropriate. A representative of the TSC and a study staff member will meet with the patient advisory board on a monthly basis.

**Patient advisory board:**	Patient and public involvement panel of the Department of General Practice, University Hospital Würzburg, Josef-Schneider-Str. 2/D7, 97080 Würzburg, Germany
**Study sites:**	The study centers in Würzburg, Tübingen and Kiel run by the respective Departments of General Medicine.
**Data Monitoring and Safety Board:**	Univ. Prof. Dr. Eva Grill, MPH<br>Institute for Medical Information Processing, Biometry and Epidemiology, Ludwig-Maximilians-Universität München<br>Marchioninistr. 15<br>81377 München<br>Tel.: +49 (0)89 440077377<br>E-Mail: eva.grill@med.uni-muenchen.de
	Birgitt Wiese, Dipl.-Math.<br>Institute for General Practice, AG Medizinische Statistik & IT-Infrastruktur, Medical School Hannover<br>Carl-Neuberg-Str. 1<br>30625 Hannover<br>Tel.: +49 (0)511 532 4414<br>E-Mail: Wiese.Birgitt@mh-hannover.de
	Univ.-Prof. Dr. Jörg Janne Vehreschild<br>Internal Medicine I (AG Kohorten in der Infektiologieforschung), University Medical Center Köln (AöR)<br>Kerpener Str. 62<br>50937 Köln<br>Tel.: +49 (0)221 478 86973<br>E-Mail: janne.vehreschild@uni-koeln.de
	Dr. med. Annika Viniol<br>Department for General Practice, Preventive and Rehabilitative Medicine, Philipps-Universität Marburg<br>Karl-von-Frisch-Straße 4<br>35032 Marburg<br>Tel.: +49 (0)6421 28 65130<br>E-Mail: annika.viniol@staff.uni-marburg.de
**Trial project management, Data management, Monitoring:**	University Hospital Würzburg, Center for Clinical Trials<br>Josef-Schneider-Straße 2, 97080 Würzburg<br>Tel.: +49 (0)931 201 3934<br>E-mail: zks_studien@ukw.de
**Production of the trial drug:**	University Hospital Hamburg-Eppendorf, Clinic Pharmacy<br>Martinistraße 52, Gebäude Nord 19, 20246 Hamburg<br>Tel.: +49 (0)40 5132629<br><br>Hevert-Arzneimittel GmbH & Co. KG<br>In der Weiherwiese 1, D-55569 Nussbaum;<br>Tel.: +49 (0)6751 910-0
**Laboratory:**	Department of diagnostic laboratory medicine, University Hospital Tübingen<br>Calwerstraße 3, 72076 Tübingen<br>Tel.: +49 (0)7071 29-80122

	E-Mail: Labor.Department@med.uni-tuebingen.de<br><br>Central Laboratory, University Hospital Würzburg<br>Department of Internal Medicine, Haus A4<br>Oberdürrbacher Straße 6, 97080 Würzburg<br>Tel.: +49 (0)931 201 45011<br>Fax: +49 (0)831 201 645011<br>E-Mail: zl-office@ukw.de<br><br>Institute of Clinical Chemistry, University Hospital Schleswig-Holstein, Kiel<br>Arnold-Heller-Straße 3, Haus U30, 24105 Kiel<br>Tel.: +49 (0)431 500 16201<br>E-Mail: Petra.Schiesewitz@uksh.de<br><br>Medical School Brandenburg CAMPUS GmbH<br>Fehrbelliner Straße 38, 16816 Neuruppin<br>Contact person: Prof. Dr. Oliver Zolk<br>Tel.: + 49 (0)33638 83-991<br>E-mail: Oliver.Zolk@mhb-fontane.de
**Trial statistician:**	Dr. Uwe Malzahn<br>University Hospital Würzburg, Center for Clinical Trials<br>Josef-Schneider-Straße 2, 97080 Würzburg<br>Tel.: +49 (0)931 201-4730<br>E-Mail: malzahn_u@ukw.de
**Collaborators:**	Medical School Brandenburg CAMPUS GmbH<br>Fehrbelliner Straße 38, 16816 Neuruppin<br>Contact person: Prof. Dr. Oliver Zolk<br>Tel.: + 49 (0)33638 83-991<br>E-mail: Oliver.Zolk@mhb-fontane.de
**Pharmacy 1:**	University Hospital Hamburg-Eppendorf, Clinic pharmacy<br>Martinistraße 52, Gebäude Nord 19, 20246 Hamburg<br>Tel.: +49 (0)40 5132629
**Pharmacy 2:**	Hevert-Arzneimittel GmbH & Co. KG<br>In der Weiherwiese 1<br>55569 Nussbaum

3 Synopsis

**Sponsor**	University Hospital Würzburg, Germany, Josef-Schneider-Str. 2/D7, 97080 Würzburg
**Title**	Prednisolone and vitamin B1, B6, and B12 in patients with Post-COVID-19-Syndrome (PC19S) – a randomized controlled trial in primary care
**Short title**	PreVitaCOV
**Indication**	PC19S
**Study design**	Double blind, randomised controlled trial, factorial design, four parallel groups, placebo-controlled, phase IIIb
**Objectives**	To assess the effectiveness, safety and feasibility of treating patients with PC19S in primary care with prednisolone and/or vitamin B1, B6, and B12 in a fixed combination.
**Summary**	PC19S affects a considerable portion of patients after an infection with SARS-CoV-2 with a broad range of disabling symptoms. Neurotropic vitamins such as vitamins B1, B6, and B12, and drugs with anti-inflammatory properties such as corticosteroids were suggested to alleviate symptoms. The trial is designed as a two-step approach that will<br>1) prove the feasibility of recruitment and retention of patients with PC19S in a primary care setting (pilot study)<br>2) investigate the effectiveness and safety of the treatment drugs alone and in combination (confirmatory study).<br>The pilot study will be transformed into a confirmatory study if feasibility is given, defined as a retention rate of 85% after enrollment of 100 patients or according to the recommendation of the DSMB.<br>In addition, blood samples will be analysed for routine parameters and vitamin B12 derivatives as well as cytokines.
**Outcome measures**	**<u>Primary endpoint</u>**<br>**Pilot study:** feasibility of screening and recruitment in primary care (retention rate of at least 85% at day 28)<br><br>**Confirmatory study:** change of symptom severity as assessed by a specifically tailored Patient Reported Outcomes Measurement Information System (PROMIS) total score referring to five symptom domains known to be typical for PC19S (fatigue, dyspnoea, cognition, anxiety, depression) from baseline to day 28<br><br>**<u>Secondary endpoints</u>**<br>**Pilot Study:**<br>1. severity, quality and change of PC19 symptoms:<br>1a. tailored PROMIS total score and sub scores in the domains included in the total score<br>1b. MYMOP<br>1c. PC19S functional status (PCFS - scale)<br>1d. PC19 symptom list<br>2. health related quality of life (EQ-5D-5L and visual analogue scale)<br>3. depression (PHQ 8)<br>4. fatigue (Chalder Scale)<br>5. numeric rating scale for pain (NRS)

	6. cognitive function: alertness, divided attention, distractibility, flexibility and visual scanning (TAP)<br>7. physical exercise (1-minute Sit-to-Stand-Test)<br>8. use of on-demand medication (patient diary)<br>9. feasibility and acceptance (survey, qualitative interviews with a sub-group sample)<br>10. physical examination (auscultation of lung and heart, orientating neurological examination, check for edema, lymph node status)<br><br>**Confirmatory study:**<br>1. severity, quality and change of PC19 symptoms:<br>1a. PROMIS subscores in the domains included in the total score<br>1b. MYMOP<br>1c. PC19S functional status (PCFS - Scale)<br>1d. PC19 symptom list<br>2. health related quality of life (EQ-5D-5L and visual analogue scale)<br>3. depression (PHQ 8)<br>4. fatigue (Chalder Scale)<br>5. numeric rating scale for pain<br>6. cognitive function: alertness, divided attention, distractibility, flexibility and visual scanning (TAP)<br>7. physical exercise (1-minute Sit-to-Stand-Test)<br>8. use of on-demand medication (patient diary)<br>9. feasibility and acceptance (survey)<br>10. physical examination (auscultation of chest and heart, orientating neurological examination, check for oedema, lymph node status)<br><br>**Safety endpoints:**<br>- number of patients with worsening symptoms 2 months after inclusion (phone call 2)<br>- number of AE and SAE by system organ class day 0 until month 2 (phone call 2)<br>- proportion of patients with at least 1 AE until day 60 (phone call 2)
**Number of patients**	To estimate the retention rate with sufficient accuracy, n=100 patients are needed. After trial completion of the 100 patients, the decision will be made whether to proceed from the Pilot Study to the confirmatory study comparing the effectiveness of treatments.<br><br>**Pilot Study:**<br>• To be assessed for eligibility (n = 200)<br>• To be allocated to trial (n = 100)<br>• To be analysed (n = 100).<br><br>**Confirmatory study (including patients of Pilot Study):**<br>• To be assessed for eligibility (n=700)<br>• To be allocated to trial (n=340) assuming 15% of patients who drop-out before day 28 (visit)<br>• To be analysed n=4x72=288 expected completers, per protocol [PP])
**Duration**	**Pilot Study:**<br><br>Overall duration: 18 months:<br>- recruitment period (months 1-8): 8 months<br>- treatment and data collection (months 1-14 months): 14 months<br>- data clearance and analysis (months 15-18):3 months<br><br>**Confirmatory study (including Pilot Study):**<br><br>Overall duration: 24 months:

	- recruitment period (months 1-14): 14 months<br>- treatment and data collection (months 1-20): 20 months<br>- data clearance and analysis (months 21-24): 3 months
**Timetable**	**Pilot Study:**<br>Time frame: 11/2022 – 04/2024<br>Trial:<br>Recruitment period: 8 months<br><br>Trail duration per patient:<br>Treatment duration: 28 days<br>Trial duration including follow-up: 6 months<br>Planned start (first patient in): 01.11.2022<br>Planned end (last patient out): 31.12.2023<br><br>**Confirmatory study (including Pilot Study):**<br>Time frame: 11/2022 – 10/2024<br>Trial:<br>Recruitment period: 14 months<br><br>Trail duration per patient:<br>Treatment duration: 28 days<br>Trial duration including follow-up: 6 months<br>Planned start (first patient in): 01.11.2022<br>Planned end (last patient out): 30.06.2024
**Inclusion criteria**	Inclusion criteria:<br>1. adult patients (at least 18 years old)<br>2. history of SARS-CoV-2 infection at least 12 weeks ago (if patients had more than one infection with SARS-CoV-2 the 12 weeks refer to the infection causing the post-COVID-19 symptoms); the infection must be documented by either a positive PCR or antibody-Test or be confirmed by the patient's GP<br>3. symptoms concerning at least one of the following domains: fatigue, dyspnea, cognition, anxiety, depression<br>4. Above mentioned symptom(s) that developed during or after the SARS-CoV-2 infection, that persist until study inclusion, and that are associated with COVID-19 as assessed by the patients' GP or the local investigator
**Exclusion criteria**	Exclusion criteria:<br>• acute Coronavirus disease (COVID-19) at baseline visit (rapid SARS-CoV-2 antigen test)<br>• patients who were treated in the intensive care unit because of COVID-19<br>• pregnancy/ breastfeeding<br>• diabetes mellitus<br>• PC19S symptoms that can be explained by an alternative diagnosis (e.g., chronic fatigue syndrome, depression, active or preceding cancer therapy, severe anemia, sleep apnea syndrome) as assessed by the patients' GP or the investigator<br>• History of severe medical conditions such as<br>○ concomitant acute infectious disease<br>○ gastrointestinal ulcer<br>○ liver disease/ liver cirrhosis<br>○ malabsorption or condition after bariatric surgery<br>○ chronic airway disease [e.g., chronic obstructive pulmonary disease

	(COPD), asthma)<br>o chronic heart failure [New York Heart Association (NYHA) III and IV]<br>o neurological disorders<br>o untreated hypothyroidism<br>o significantly impaired glucuronidation (e.g., Gilbert-Meulengracht, ROTOR, or Crigler-Najjar syndrome)<br>o immunodeficiency or a chronically weakened immune system [e.g., acquired immunodeficiency syndrome (AIDS), HIV, lymphoma, chemo-radio- therapy, immunosuppressive pathology]<br>o mental disorders<br>o active cancer<br>o any other severe medical conditions that preclude participation as determined by responsible physiciancurrent use of<br><br>• Current concomitant medication with:<br>o immunosuppressive drugs<br>o non-steroidal antiinflammatory drugs (NSAID), ASS, Indometacin<br>o Fluoroquinolones<br>o anticoagulation: phenprocoumon or other cumarin derivates, direct oral anticoagulants<br>o any other drug that could exhibit clinically relevant interactions with the study medication (as described in Fachinformation Prednisolon STADA®, Predni H Tablinen® Zentiva or Fachinformation Vitamin B komplex Hevert). The decision on the clinical relevance of the interactions is at the discretion of the clinical investigator.<br>• systemic treatment with prednisolone for at least 7 days or any parenteral application since the end of the acute phase of COVID-19; treatment with vitamins B1, B6, or B12 in doses equivalent to the dose of the study medication for at least 7 days or any parenteral application since the end of the acute phase of COVID-19; vitamin supplements containing vitamin B1, B6, or B12 should have been ceased at least 4 weeks prior to the inclusion of the study.<br>• known allergies and contraindications to the intervention drugs<br>• need of care and/or peer dependency<br>• nursing home residents<br>• inability to understand the scope of the study, to follow study procedures and to give informed consent or to attend the study sites<br>• participation in another interventional trial at the same time or within the past 3 months before enrollment<br>• female patients considering to get pregnant during the first month of the trial and within 1 week after the last dose of study drug(s)
**Clinical trial procedure**	• Prescreening upon scheduling of appointment (verification of in-/exclusion criteria, checking of eligibility for interviews)<br>• Day 0: enrollment: information, informed consent, data protection sheet, randomization to trial treatment, verification of in-/exclusion criteria, medical history, concomitant medication and psychoactive substances, demographic and basic data regarding COVID-19 infection, mental fitness (MoCA) and physical activity, TAP, vital signs and biosamples, collection of baseline data for outcome measures (Visit 1)<br>• Days 1-28: drug intake, patient diary (to be filled out by the patient)<br>• Day 5 (+ 3 days): outcome measures, concomitant medication, (S)AEs. (Phone call 1)<br>• Days 1-7 measure blood pressure once a day<br>• Day 28 (+ 3 days): outcome measures, laboratory and vital signs, concomitant medication, (S)AEs, drug accountability (Visit 2)<br>• Day 32 (+3 days): second pregnancy test<br>• Day 60 (+/-5 days): outcome measures, (S)AEs. (Phone call 2, follow-up survey)

	• Day 180 (+/- 7 days): outcome measures, survey/interview on feasibility and acceptance (Phone call 3, close-out), (S)AEs.
**Trial drugs**	• Trial drug 1: Prednisolone 20 mg for five days followed by 5 mg for 23 days<br>• Trial drug 2: Vitamin B1 (100 mg), B6 (50 mg), and B12 (500 µg) for 28 days<br>• Trial drug 3: Trial drugs 1 and 2<br>• Trial drug 4 (comparator): placebo
**Statistical analysis**	• **Primary endpoint of Pilot Study: retention rate at day 28**<br>Proportion of included patients who completed the 4-week intervention, point and interval estimations (95% CI, confidence interval) combining all 4 treatment groups.<br>• **Primary endpoint of Confirmatory study: symptom severity**<br>Change in the tailored PROMIS total score from baseline to day 28 to be analyzed in a factorial analysis of variance (ANOVA). The two binary factors are treatment with vitamin B compounds (no/yes) and treatment with prednisolone (no/yes).<br>For all secondary endpoints total sum scores and if applicable subscores will be calculated for the different visits together with the corresponding change scores from baseline to day 28. Where appropriate, convenient transformations will be done.<br>• **Safety:** number of patients with worsening symptoms 2 months after inclusion (phone call 2), frequencies of (S)AEs, proportion of patients with at least 1 AE day 0-60 (phone call 2) to be reported for each group.
**Funding**	The clinical trial is funded by the Federal Ministry of Education and Research (FKZ 01EP2109A (Würzburg), FKZ 01EP2109B (Tübingen), FKZ 01EP2109C (Kiel).

4 Synopse

**Sponsor**	Universitätsklinikum Würzburg, Deutschland, Josef-Schneider-Str. 2/D7, 97080 Würzburg
**Titel**	Prednisolon und Vitamin B1, B6 und B12 für Patient/innen mit Post-COVID-19-Syndrom (PC19S) – eine randomisierte kontrollierte Studie in der hausärztlichen Versorgung.
**Kurzbezeichnung**	PreVitaCOV
**Indikation**	PC19S
**Studiendesign**	Doppelblinde, randomisierte kontrollierte Studie, faktorielles Design, vier parallele Gruppen, Placebo-kontrolliert, Phase IIIb
**Ziele der klinischen Prüfung**	Untersuchung der Wirksamkeit, Sicherheit und Machbarkeit der Behandlung von Patienten /Patientinnen mit PC19S in der hausärztlichen Versorgung mit Prednisolon und/oder Vitamin B1, B6 und B12 in einer festen Zusammensetzung.
**Zusammenfassung**	PC19S betrifft einen beträchtlichen Teil der Patientinnen/Patienten nach einer Infektion mit SARS-CoV-2 und führt zu einem breiten Spektrum beeinträchtigender Symptome. Neurotrope Vitamine wie die Vitamine B1, B6 und B12 sowie Medikamente mit entzündungshemmenden Eigenschaften wie Kortikosteroide wurden vorgeschlagen, um die Symptome zu lindern. Die Studie besteht aus den folgenden zwei Phasen:<br>1) Nachweis der Machbarkeit der Rekrutierung und des Studienverbleibs von Patienten und Patientinnen mit PC19S in der Hausärztlichen Versorgung (Pilotstudie).<br>2) Untersuchung der Wirksamkeit und Sicherheit der Medikamente allein und ihrer Kombination (konfirmatorische Studie).<br>Die Pilotstudie wird in eine konfirmatorische Studie umgewandelt, wenn die Machbarkeit bestätigt werden kann. Diese ist definiert als ein Studienverbleib von mindestens 85 % nach dem Einschluss von 100 Patient/innen.<br>Außerdem werden Blutproben entnommen und daraus Routineparameter, Vitamin B12-Derivate und Zytokine bestimmt.
**Zielgrößen**	**Primärer Endpunkt**<br>**Pilotstudie:** Machbarkeit des Screenings und der Rekrutierung in der hausärztlichen Versorgung (Studienverbleibquote von mindestens 85 % am Tag 28 oder nach Einschätzung des DSMB)<br>**Konfirmatorische Studie:** Veränderung der Symptomschwere, gemessen mit einem Fragebogen zu patientenorientierten Outcomes: PROMIS-Gesamtscore, spezifisch zugeschnitten auf fünf Symptombereiche, die typisch für PC19S sind (Müdigkeit, Dyspnoe, Kognition, Angst, Depression).<br>**Sekundäre Endpunkte**<br>**Pilotstudie**<br>1. Schweregrad, Art und Veränderung der PC19-Symptome erfasst durch:<br>1a. PROMIS Gesamt – und Subscores für die Bereiche, die im Gesamtscore enthalten sind<br>1b. MYMOP<br>1c. PC19S-Funktionsstatus (PCFS-Skala)

	1d. PC19-Symptomliste<br>2. Gesundheitsbezogene Lebensqualität (EQ-5D-5L und visuelle Analogskala)<br>3. Depression (PHQ 8)<br>4. Müdigkeit/ Fatigue (Chalder-Skala)<br>5. Numerische Ratingskala für Schmerz<br>6. Kognitive Funktion: Alertness, geteilte Aufmerksamkeit, Ablenkbarkeit, Flexibilität und visuelles Scanning (Testbatterie zur Aufmerksamkeitsprüfung, TAP)<br>7. Körperliche Ausdauer (1-Minuten-Sit-to-Stand-Test)<br>8. Verbrauch an Bedarfsmedikation (Patiententagebuch)<br>9. Durchführbarkeit und Akzeptanz (Fragebogen, qualitative Interviews mit einer Untergruppe der Studienteilnehmer/innen)<br>10. Körperliche Untersuchung (Auskultation von Herz und Lunge, orientierende neurologische Untersuchung, Untersuchung auf Ödeme und Lymphknoten)<br><br>**Konfirmatorische Studie**<br>1. Schweregrad, Art und Veränderung der PC19-Symptome erfasst durch:<br>1a. PROMIS-Subscores für die Bereiche, die im Gesamtscore enthalten sind<br>1b. MYMOP<br>1c. PC19S-Funktionsstatus (PCFS-Skala)<br>1d. PC19-Symptomliste<br>2. Gesundheitsbezogene Lebensqualität (EQ-5D-5L und visuelle Analogskala)<br>3. Depression (PHQ 8)<br>4. Müdigkeit/ Fatigue (Chalder-Skala)<br>5. Numerische Ratingskala für Schmerz<br>6. Kognitive Funktion: Alertness, geteilte Aufmerksamkeit, Ablenkbarkeit, Flexibilität und visuelles Scanning (Testbatterie zur Aufmerksamkeitsprüfung, TAP)<br>7. Körperliche Ausdauer (1-Minuten-Sit-to-Stand-Test)<br>8. Verbrauch an Bedarfsmedikation (Patiententagebuch)<br>9. Durchführbarkeit und Akzeptanz (Befragung), Machbarkeit (Studienverbleibquote an Tag 28 aller eingeschlossenen Patient/Innen)<br>10. Körperliche Untersuchung (Auskultation von Herz und Lunge, orientierende neurologische Untersuchung, Untersuchung auf Ödeme und Lymphknoten)<br><br>**<u>Sicherheitsendpunkte</u>**<br>- Anzahl der Patientinnen/Patienten mit sich verschlechternden Symptomen 2 Monate nach Einschluss (2. Telefonat)<br>- Anzahl der unerwünschten Ereignisse (AE) und unerwünschten schwerwiegenden Ereignisse (SAE) nach Organsystem von Tag 0 bis Tag 60 (2. Telefonat)<br>- Anteil der Patient/innen mit mindestens 1 AE bis Tag 60 (2. Telefonat)
**Anzahl der Teilnehmenden**	Um die Verbleibquote in der Studie mit ausreichender Genauigkeit zu schätzen, werden n=100 Patientinnen/Patienten benötigt. Nach Abschluss der Studie mit 100 Patienten/Patientinnen wird entschieden, ob die Pilotstudie in eine konfirmatorische Studie überführt wird, in der die Wirksamkeit der Behandlungen verglichen wird.<br>**Pilotstudie:**<br>- Prüfung der Ein- und Ausschlusskriterien (n = 200)<br>- Einschluss in die Studie (n = 100)<br>- Datenanalyse (n = 100).

	**Konfirmatorische Studie (beinhaltet die PatientInnen der Pilotstudie):**<br>- Prüfung der Ein- und Ausschlusskriterien (n=700)<br>- Einschluss in die Studie (n=340), bei einer angenommenen Drop-out Quote von 15% vor Tag 28 (2. Visite)<br>- Datenanalyse von n=4x72=288 erwartete Patienten, die die Studie gemäß Protokoll abschließen
**Dauer**	**Pilotstudie:**<br>18 Monate: Rekrutierungszeitraum (Monate 1-8); Behandlung und Datenerhebung (Monate 1-14 Monate); Datenbereinigung und -analyse (Monate 15-18).<br><br>**Konfirmatorische Studie (die Dauer der Pilotstudie ist hier miteingeschlossen):**<br>24 Monate: Rekrutierungszeitraum (Monate 1-14); Behandlung und Datenerhebung (Monate 1-20); Datenbereinigung und -analyse (Monate 21-24).
**Zeitplan**	**Pilotstudie:**<br><u>Zeitraum:</u> 11/2022 – 04/2024<br><u>Trial:</u><br>Rekrutierungszeitraum: 8 Monate<br><u>Trialdauer pro Patient:</u><br>Behandlungsdauer: 28 Tage<br>Studiendauer inklusive Follow-up: 6 Monate<br>Geplanter Start (erster Patienteneinschluss): 01.11.2022<br>Geplantes Ende (letzter Patientenausschluss): 31.12.2023<br><br>**Konfirmatorische Studie (einschließlich Pilotstudie):**<br><u>Zeitraum:</u> 11/2022 – 10/2024<br><u>Trial:</u><br>Rekrutierungszeitraum: 14 Monate<br><br><u>Trialdauer pro Patient:</u><br>Behandlungsdauer: 28 Tage<br>Studiendauer inklusive Follow-up: 6 Monate<br>Geplanter Start (erster Patienteneinschluss): 01.11.2022<br>Geplantes Ende (letzter Patientenabschluss): 30.06.2024
**Einschlusskriterien**	**Einschlusskriterien:**<br>1. Erwachsene ab 18 Jahren<br>2. SARS-CoV-2-Infektion in der Vorgeschichte vor mindestens 12 Wochen; die Infektion muss entweder durch einen positiven PCR- oder Antikörper-Test dokumentiert sein oder durch die Hausärztin/den Hausarzt bestätigt werden; wenn Patienten mehr als eine Infektion mit SARS-CoV-2 hatten, beziehen sich die 12 Wochen auf die Infektion, die die Post-COVID-19-Symptome auslöste<br>3. Symptome, die mindestens einen der folgenden Bereiche betreffen: Müdigkeit, Dyspnoe, Kognition, Angstzustände, Depression<br>4. Oben genannte Symptome, die sich während oder nach der SARS-Cov-2 Infektion entwickelten, die zum Zeitpunkt des Studieneinschlusses fortbestehen und die mit der COVID-19-Infektion in Zusammenhang gebracht werden (nach klinischer Einschätzung der Hausärztin/des Hausarztes oder der Prüfärztin/des Prüfarztes).

Ausschlusskriterien	Ausschlusskriterien:
	1. akute COVID-19-Infektion bei der Baselineuntersuchung (positiver SARS-CoV-2 Antigen-Schnell-Test)<br>2. Patienten/Patientinnen, die aufgrund von COVID-19 auf der Intensivstation behandelt wurden<br>3. Schwangerschaft/ Stillen<br>4. Diabetes mellitus<br>5. PC19S-Symptome, die vom Hausarzt/von der Hausärztin oder vom Studienarzt/der Studienärztin durch eine andere Diagnose erklärt werden können (z.B. chronisches Müdigkeitssyndrom, Depression, aktive oder vorausgegangene Krebstherapie, schwere Anämie, Schlafapnoe-Syndrom),<br>6. Schwerwiegende medizinische Erkrankungen in der Vorgeschichte, z.B.:<br>• begleitende akute Infektionskrankheit<br>• gastrointestinales Ulcus<br>• Lebererkrankung/Leberzirrhose<br>• Malabsorption oder Zustand nach bariatrischer Operation<br>• chronische Atemwegserkrankungen (z. B. Asthma, COPD)<br>• chronische Herzinsuffizienz (NYHA III und IV)<br>• neurologische Störungen<br>• signifikante Einschränkung der Glucuronidierungsfähigkeit (z.B. Morbus Gilbert-Meulengracht, ROTOR- oder Crigler-Najjar-Syndrom<br>• unbehandelte Hypothyreose<br>• Immunschwäche oder ein chronisch geschwächtes Immunsystem (z. B. AIDS, HIV, Lymphome, Radio-Chemotherapie, immunsuppressive Pathologie)<br>• psychische Störungen<br>• aktive Krebserkrankung<br>• andere schwerwiegende medizinische Gründe, die eine Teilnahme ausschließen (nach Maßgabe der/des zuständigen Prüfärztin/Prüfarztes)<br><br>7. Derzeitige Einnahme von:<br>• Immunsuppressiven Medikamenten<br>• Nichtsteroidalen Antiphlogistika (NSAID), ASS, Indometacin<br>• Fluorchinolonen<br>• Antikoagulation: Phenprocoumon oder andere Cumarinderivate, direkte orale Antikoagulantien<br>• Anderen Arzneimitteln, die klinisch relevante Wechselwirkungen mit der Studienmedikation aufweisen könnten, wie in der Fachinformation Prednisolon STADA® , Predni H Tablinen® Zentiva oder Fachinformation Vitamin B Komplex Hevert beschrieben. Die Entscheidung über die klinische Relevanz der Wechselwirkungen liegt im Ermessen des Prüfarztes/der Prüfärztin.<br><br>8. Systemische Behandlung mit Prednisolon für mindestens sieben Tage seit dem Ende der akuten Phase von COVID-19; Behandlung mit Vitamin B1, B6 oder B12 in einer Dosis, die der Prüfmedikation entspricht, für mindestens 7 Tage oder irgendeine parenterale Verab-

	reichung seit dem Ende der akuten Phase von COVID-19; Nahrungsergänzungsmittel, die Vitamin B1, B6 oder B12 enthalten, sollten mindestens vier Wochen vor Studieneinschluss abgesetzt werden.<br>9. Bekannte Allergien oder Kontraindikationen gegen die Interventionsmedikamente<br>10. Pflegebedürftigkeit und/oder Peer-Abhängigkeit<br>11. Bewohner/Bewohnerinnen eines Pflegeheims<br>12. Unfähigkeit, den Umfang der Studie zu verstehen, den Studienverfahren zu folgen, eine informierte Zustimmung zu geben oder den Besuch im Studienzentrum wahrnehmen zu können<br>13. Zeitgleiche Teilnahme an einer anderen interventionellen Studie oder Teilnahme innerhalb der letzten drei Monate<br>14. Frauen mit Schwangerschaftswunsch während und 1 Woche nach Ende der Medikationsphase
**Ablauf der klinschen Prüfung**	- Prescreening im Rahmen der Terminvereinbarung (Prüfen der Ein-/Ausschlusskriterien, Prüfen der Bereitschaft zur Teilnahme an Interviews)<br>- Tag 0: Aufnahme: Information, Einverständniserklärung, Datenschutzerklärung, Überprüfung der Ein-/Ausschlusskriterien, Randomisierung zur Studienbehandlung Anamnese, Begleitmedikation und psychoaktive Substanzen, Demografie und Basisdaten bezüglich COVID-19, mentale Fitness (MoCA) und körperliche Aktivität, TAP, Vitalparameter und Labor, Erhebung der Baselinedaten zu den Ergebnismessungen (Visite 1)<br>- Tage 1-28: Medikamenteneinnahme, Patiententagebuch (vom Patienten auszufüllen)<br>- Tag 5 (+ 3 Tage): Ergebnismessungen; Begleitmedikation, (S)AEs (Telefonat 1)<br>- Tag 1 - 7 Blutdruckmessungen<br>- Tag 28 (+ 3 Tage): Ergebnismessungen, Labor und Vitalparameter, Begleitmedikation, (S)AEs, Kontrolle der verbliebenen Studienmedikamente (Visite 2)<br>-<br>- Tag 32 (+3 Tage): zweiter Schwangerschaftstest für gebärfähige Frauen<br>- Tag 60 (+/- 5 Tage): Ergebnismessungen, (S)AEs (Telefonat 2, Follow-up-Erhebung)<br>- Tag 180 (+/- 7 Tage): Ergebnismessungen, Machbarkeit und Akzeptanz (Telefonat 3, Abschluss), (S)AEs.
**Prüfpräparat**	**Prüfpräparat 1**: Prednisolon 20 mg für fünf Tage gefolgt von 5 mg für 23 Tage<br>**Prüfpräparat 2**: Vitamin B1 (100 mg), Vitamin B6 (50 mg), Vitamin B12 (500 µg) für 28 Tage<br>**Prüfpräparat 3**: Prüfpräparat 1 und 2<br>**Prüfpräparat 4** (Vergleichspräparat): Placebo
**Statistische Analyse**	• **Primärer Endpunkt der Pilotstudie**: **Patientenverbleib an Tag 28:**<br><br>Anteil der eingeschlossenen Patienten, die die vierwöchige Intervention abgeschlossen haben, Punkt- und Intervallschätzung (95% CI, Konfidenzintervall), die alle 4 Behandlungsgruppen zusammenfassen.

	• **Primärer Endpunkt der konfirmatorischen Studie**: **Symptomschwere:**<br>Änderungen des PROMIS-Gesamtscores von der Baseline bis zum Tag 28, der in einer faktoriellen Varianzanalyse (ANOVA) getestet werden soll. Die beiden binären Faktoren sind die Behandlung mit Vitamin B komplex (nein/ja) und die Behandlung mit Prednisolon (nein/ja).<br>Für alle sekundären Endpunkte werden Gesamtsummenwerte und ggf. Subscores für die verschiedenen Besuche zusammen mit den entsprechenden Änderungswerten der Scores von der Baseline bis zum Tag 28 berechnet. Sofern angebracht, werden Datenumwandlungen durchgeführt.<br>• **Sicherheit:**<br>Anzahl der Patienten mit sich verschlechternden Symptomen 2 Monate nach der Aufnahme (Telefonat 2), Häufigkeit von (S)AEs, Anteil der Patienten je Gruppe mit mindestens 1 AE Tag 0-60 (Telefonanruf 2).
**Finanzierung**	Die klinische Studie wird finanziert durch das Bundesministerium für Bildung und Forschung (BMBF) FKZ 01EP2109A (Würzburg), FKZ 01EP2109B (Tübingen), FKZ 01EP2109C (Kiel).

5 Visit plan

**Patient contact**	**Base-line**												**Follow-up**
**Day**	0	1-4	5*	1-7**	6-13	14	15-20	21	22-27	28*	32***	60****	180*****
**Visit number**	1									2			
**Phone call**			1									2	3
**Trial drug intake**		X	X		X	X	X	X	X	X			
information, verification of in-/exclusion criteria, informed consent	X												
Demographic and baselinedata, Medical history, MoCA	X												
Biosamples, vital signs	X			X						X			
2nd pregnancy test											X		
**Outcomes**													
tailored PROMIS total score and subscores	X		X			X		X		X		X	X
MYMOP	X		X							X		X	X
PC19 Symptom list	X		X							X		X	X
PCFS - Scale	X		X							X		X	X
EQ-5D-5L and visual analogue scale	X		X							X		X	X
PHQ- 8	X		X							X		X	X
Chalder Fatigue Scale	X		X							X		X	X
Numeric rating scale for pain	X		X							X		X	X
Tests Alertness, divided attention, distractibility, flexibility and visual scanning (TAP)	X									X			
1min-Sit-to-Stand-Test, physical examination	X									X			
**Patient diary** (on-demand medication		X	X		X	X	X	X	X	X			
Feasibility & acceptance questionnaire													X
Feasibility & Acceptance Interviews (subgroup of patients)	X												
**Safety outcomes**													
Adverse events			X							X		X	X
Serious adverse events							as necessary						
Drug accountability			X							X			

* + 3 days; **only blood measurement,***+ 2 days; ****+/- 5 days; ***** +/-7 days.

6 Introduction

6.1 Background of the clinical trial

Scientific Background, preliminary Data and existing Infrastructure

Post-COVID-19 syndrome (PC19S) is a new pathologic entity affecting a considerable portion of patients (dependent on the population studied and the defined duration) after an infection with SARS-CoV-2.[1-5] A population-based survey from Germany that included 1,459 patients reports a prevalence of 49% for persisting symptoms after COVID-19.[3] PC19S is characterised by a broad range of persistent disabling symptoms and signs.[6] The most frequent symptoms include fatigue, post-exertional malaise, cognitive dysfunction, impaired attention, dry cough, shortness of breath, headache, muscle ache, chest tightness, and sore throat.[7] Most patients seek help in ambulatory care; and 76% exclusively consult their General Practitioner.[8] The German S1-Guidelines summarize the current recommendations and options in PC19S care. So far, there is no evidence on specific medical treatment options for these patients. The lack of swiftly initiated trials that explicitly take place in primary care in Germany and generate valid results reflects the lack of infrastructure and preparation.

The exact pathophysiological mechanism of PC19S is unknown. Several mechanisms have been proposed as pathophysiological explanations, including long-term tissue damage and chronic auto-inflammation.[9] Preliminary findings suggest that cytokine signature differs between PC19S and convalescent COVID-19 patients without persistent symptoms.[10] Thus, an Italian study with 551 patients using a multiplex immunoassay found an abnormal hyperinflammatory cytokine profile in patients with persisting symptoms compared to a control.[10] Furthermore, the varied symptoms of PC19S might be induced by an increased demand for methyl-groups in the organism caused by SARS-CoV2 while impairing their supply due to the viral-induced cytokine storm and oxidative stress.[11] Accordingly, patients with PC19S may benefit from methyl-group support e.g. by therapy with vitamin B12. Other authors suggest vitamins B1, B6, and B12 due to their neurotropic effects considering that most patients suffer from fatigue or other neurologic symptoms.[12] For PreVita-COV, we chose vitamin B1, B6, and B12 due to the above-mentioned possible effects.

Drugs with anti-inflammatory properties such as corticosteroids, among others, were also suggested to alleviate symptoms.[9] This approach is based on the idea of chronic inflammation, as has already been postulated for various autoimmune diseases[13] and the PC19S[14]. An observational study including 30 patients showed that treatment with corticosteroids at high initial dose and a rapid wean over a few weeks was well tolerated and associated with improvement of post-COVID-19 pulmonary symptoms [15]. In another study, but with significant limitations in study design, 24 patients diagnosed with Long-COVID were treated with corticosteroids in tapering doses for 8 to 10 weeks. The study reports improvement in fatigue and breathlessness, among other, but did not include a control group.[16] A Spanish study treated eight patients with 30mg prednisolone for a total of four days. While the focus was on normalization of immunologic laboratory findings, also the clinical parameters are reported to have improved.[17] Therefore, we chose corticosteroids as a further therapeutic approach for PC19S.

Both treatments are well known, easy to monitor in general practice, and have only few side effects and require little effort overall. Due to their different mechanisms of action, we expect additive effects when combining them using a factorial design. PreVitaCOV will bring together different research disciplines including methodological expertise and demonstrate distinguished access to patients

through local GP-networks. This is highly relevant for primary care practice and will strengthen sustainable scientific practice, especially the performance of therapeutic RCTs in ambulatory care. The members of the consortium are experienced in RCTs in general practice[18], in epidemiological and cohort studies and are conducting several studies on COVID 19 and PC19S. They have established practice-based research networks such as the Bavarian Research Network in General Practice[19] and the Research Practice Network Baden-Württemberg[20] that will support this study. SJ and IG are spokespersons of the Fach- und Organspezifischen Arbeitsgruppen (FOSA)[21] General Practice of the German National Pandemic Cohort Network (NAPKON)[22], PH is member steering board and coordinates the ECU within NAPKON. The German College of General Practitioners and Family Physicians (DEGAM)[23] in the Expert Advisory Panel on Pandemic Respiratory Infections of the Robert Koch Institute is represented by HK as she is the spokesperson of the Infectiology Working Group of the DEGAM and co-author of the DEGAM-Practice Guideline on SARS-COV-2 and COVID-19[24]. WM is a clinical geriatrician and the Head of Neurogeriatrics at the Neurology Department of Kiel University. His special focus is on safety, benefits and risks of the elderly study participants.

Description of and justification for the route of administration, dosage, dosage regimen, and treatment period(s).

Based on the above-mentioned rationale and the urgent need for effective, evidence-based treatment options in PC19S, this trial will investigate two drugs that have been approved for years and that are successfully used in many clinical conditions:

<u>Prednisolone</u>

Like all corticosteroids, Prednisolone is a potent anti-inflammatory drug used in a variety of auto inflammatory or rheumatoid conditions. As stated above, chronic (auto-) inflammation is discussed to be a potential aetiology of PC19C.[7, 14]

The most effective regimen is a pulse therapy with high initial doses like it is used in many (auto-) inflammatory conditions. A first observational study showed that treatment with corticosteroids at a high initial dosage and a rapid wean over a few weeks was well tolerated and associated with improvement of post-COVID-19 pulmonary symptoms.[15] In order to balance therapy efficiency and potential side effects, we will therefore administer Prednisolone in a dosage of 20mg during the first five days, a dosage that is considered to be a medium dosage.[25] Given its rapid anti-inflammatory effect, changes in clinical manifestations of (auto-) immune diseases can usually be observed within a few days. Therefore, we will ask participants for their symptoms on day five.

The regimen will continue with a dose of 5mg after day five in order to maintain the anti-inflammatory effect after the initial induction. A second rationale is the potential effect on patients with low blood levels of cortisol since low cortisol concentrations were recently reported in patients with PC19S.[26] After long-term use of corticosteroids, the medication should not be stopped or reduced abruptly in order to avoid an adrenal insufficiency. Therefore, some regimes with long-term use require a tapered reduction before cessation of the medication. In the present study, however, we will use Prednisolone for only five days in a dosage with potential suppression of the adrenal gland. A tapered regime is therefore not required.[27]

The daily dose of [28] Prednisolone will be taken in the morning, thus reflecting the physiological circadian rhythm of cortisol secretion. The medication will be administered orally because of its rapid

absorption and almost complete bioavailability.[29] The maximum concentration in the blood is observed after one to two hours.

For further details concerning the composition of the drug please see chapter 10 “Trial Medication” and the Investigational Medicinal Product Dossier (IMPD) of the pharmacy.

Vitamin B1, B6, and B12

According to the above-mentioned rationale, we choose vitamins B1 (thiamine), B6 (pyridoxine), and B12 (cyanocobalamin) because of their potential to alleviate viral-induced inflammation, cytokine storm, and oxidative stress [11] as well as because of their positive neurotropic effects [12].

The dosage used in this trial will be same as used for supplementation of the respective vitamin deficiencies[30]: thiamine 100mg, pyridoxine 50mg, and Cyanocobalamin 500 µg. While the European Food Safety Authority (EFSA) defined no upper limits for Vitamin B1 and B12, the upper limit for Vitamin B6 was set to 25mg per day.[28] This is because of possible neurotoxic side effects. However, these were only observed after long term use of vitamin supplementation. Therefore, a short-term use over 4 weeks is considered to be safe with a dosage of 50mg. In addition, the National Institute of Health sets the upper limit for pyridoxine to 100mg per day.[31]

The medication will be taken once daily in the morning. It will be administered orally because of its good bioavailability. To limit the risk of malabsorption we will exclude participants with gastric ulcer, history of bariatric surgery, or known malabsorption.

For further details concerning the composition of the drug please see chapter 10 “Trial Medication” and the Investigational Medicinal Product Dossier (IMPD) of the pharmacy.

6.2 Rationale for the trial

Scientific Objectives and Relevance

The study is divided into two parts:

The pilot study aims to demonstrate feasibility of a pragmatic randomised controlled trial (RCT) with vitamin B1, B6, and B12 and prednisolone in primary care patients with PC19S. Specifically, it will inform on feasibility of recruitment and retention of patients in primary care, and on data collection (including bio samples).

If feasibility is confirmed, the trial will be transformed into a confirmatory RCT to prove the effectiveness of the treatment approaches.

By involving general practices and health care professionals in university hospitals, the study team will establish proceedings for future RCTs and provide a use case for existing infrastructures such as practice-based research networks and the NAPKON[22].

Expected Results

- Meeting recruitment targets, retention of 85% of patients,
- (Preliminary*) data on effectiveness of the treatment approaches for patients with PC19S which are simple to handle and of low risk.

Dissemination and Exploitation

The results of this trial will be fed directly into the Bavarian Practice Based Research Network (www.BayFoNet.de), the Research Practice Network Baden-Württemberg (www.forschungspraxen-netz-bw.de) and throughout Germany via the Initiative of German Research Practice Networks (www.desam-fornet.de) through presentations, publication in newsletters.[19, 20, 32] Data will be provided in an open data repository and to existing infrastructures (NAPKON, www.napkon.de). A cooperation with NAPKON will further inform about recruitment findings. Due to personal activities of the applicants IG, SJ and HK on several levels of the DEGAM, incorporation of the findings into the S1 treatment recommendation of the DEGAM on the SARS-CoV-2 virus and into the Journal of General Practice after peer review is given.

Future impact with regard to improvement of patient care

Most patients with suspected PC19S seek help from their GPs. Therefore, it is necessary to investigate which treatment options are effective and feasible and that can be easily guided by GPs and implemented in primary care.

Ethical justification

This trial is ethically justifiable because the applied drugs have been approved for years and no significant side effects are expected for the targeted dosages and time period.

Clinical relevance

In view of the fact that there are currently no effective and empirically well-tested treatment methods for PC19S, the clinical relevance is to be classified as very high in view of the high frequency of the symptom and the fact that the pandemic has still not subsided yet.

Estimation of transferability into practice

The trial is conducted with a sample of patients recruited in primary care. This will facilitate transferability of the findings to patients with PC19S. The administration of the trial drugs is oral and can be taken by patients at home. No specific room and equipment for drug storage is required. We expect that most patients are familiar with the trial drugs and will adhere to the dosing schedule.

Results of the trial will be published in national and international journals. In case of positive results, these will be introduced into guidelines and GP training/ quality circles, in order to implement them into medical care.

* If only Phase 1 will be conducted.

6.3 Assessment of risks and benefits

Benefits

There are no known benefits of the trial so far. Based on the rationale in chapter 6.1., however, potential benefits for participants are the chance that the severity of symptoms of PC19S will be reduced by the trial medication.

Since this hold true for participants in the drug group only, participants in the placebo group will also benefit from the expected effect that the trial itself will positively influence symptoms. It is generally accepted that being enrolled in a clinical study may already have an impact on the disease and symptom severity. Some authors refer to this observation as "context effect".[33] It might partially be explained by the attention and counselling study participants receive during the study. We expect that this context effect is particularly strong in patients with PC19S because many patients say they do not find appropriate help in the health care system at the moment (own data, under revision at Annals of Family Medicine). Therefore, the mere fact of being taken seriously could have an impact on symptoms. A recent study involving patients with PC19S and olfactory impairment showed that 42% of participants in the placebo group improved.[34]

Risks

The medication used in this trial is known for years and is safely used in many clinical conditions. However, a potential risk for trial participants are the known side effects of the trial medication, notably susceptibility to infections or worsening of diabetes mellitus, although, these are manageable given the low dosage and short timespan used in this study. Patients will be monitored closely for possible side effects and participants with conditions that could worsen by the medication are excluded from the study. Any AEs and SAEs will be documented and discussed regularly with the members of the DSMB. No trial-related invasive procedures are planned other than a blood sample taken twice (approx. 25ml). Participants will need to attend the study site twice during the trial and are asked to fill in questionnaires, to perform some cognitive and physical tests.

Summary

The possibility of benefiting from an effective treatment for PC19S symptoms is contrasted by the side effects that can occur from the medications. As long as there are no evidence-based treatments for PC19S so far, and given the urgent need for effective treatments for these patients, we believe the side effects are manageable, provided the medication is administered for only four weeks and at low doses, and the in- and exclusion criteria protect patients at risk.

The amount of time participants spend on the study and visiting the study sites are offset by the positive effects expected for participants in both the drug and placebo groups because of the counselling on PC19S by specialists.

Thus, a) the individual patient benefits in terms of drug effectiveness, safety and costs, and b) benefits from increased knowledge about the treatment of a condition that is characterized by a high clinical relevance and prevalence. Therefore, we consider that the benefits justify the risks for participants in this study.

Risks and benefits for the elderly study participants

The gastric mucosa in older age is characterised by poorer blood supply and a decline in its protective function. Therefore, the elderly have a higher risk of GI ulcers and bleeding. We did not find scientific evidence for the development of steroid ulcers in clinical practice. Corticosteroids alone become ulcerogenic if treatment lasts longer than one month and the total administered dose exceeds 1000 mg.[35]

The use of non-steroidal anti-inflammatory drugs (NSAIDs) in combination with corticosteroids can increase the risk of ulcers. To reduce the risk of ulcers the use of NSAIDs is an exclusion criterion – for all study participants. Also, patients with a history of gastrointestinal ulcer will be excluded.

Corticosteroids also have a diabetogenic effect. Therefore, unstable or untreated diabetes mellitus is an exclusion criterion and non-fasting glucose is part of laboratory testing at baseline and at day 28. Iatrogenic osteoporosis from prolonged therapy with glucocorticoids is possible, too. However, the treatment duration of 28 days limits the risk of secondary diabetes and osteoporosis. With regard to osteoporosis prophylaxis with vitamin D and calcium is not needed when the treatment duration is limited to 28 days.

Patients will be informed about the possible side effects before the start of the study and will be motivated to contact the study centre or their GP in case they develop symptoms.

Since we do not know whether and to what extent the participants with Post-COVID-19 benefit from the medication, it is important to include older patients in the study - also in order to be able to measure any age-dependent benefits. With increasing age, it becomes more and more difficult to compensate for functional losses. Therefore, it is particularly important to consider therapeutic options for Post-COVID-19 in older people.

Risks due to metabolization of Prednisolone

Prednisolone is metabolized mainly in the liver to about 70% by glucuronidation. There are numerous UDP-glucuronosyltransferases, only a few of which are important for the metabolism of prednisolone. Prednisolone is metabolized to prednisone, and both are glucuronidated primarily by the UDP-glucuronosyltransferase UGT3B7 (see for example[36]) and with a much lower glucuronidation activity by 1A3 and 2B17. Prednisolone does not itself lead to clinically relevant induction or inhibition of UGTs.

Strong inhibitors of UGT enzymes found in databases include efavirenz (strong inhibition of UGT 3B7, UGT1A4, and UGT2B7) and pazopanib (strong inhibition of UGT1A1). However, interactions between efavirenz and prednisolone are overridden by CYP-dependent interactions. Co-administration with efavirenz may decrease the plasma concentrations of prednisolone, which is a substrate of the CYP3A4 isoenzyme. The mechanism is accelerated clearance due to induction of CYP3A4 activity by efavirenz. The potent UGT1A1 inhibitor pazopanib does not lead to a UGT-dependent interaction with prednisolone (which is not a substrate of UGT1A1).

Clinically important drug interactions are already considered in the protocol. Co-medication with drugs that can interact with prednisolone according to the Summary of product characteristics is defined as an exclusion criterion in the protocol. In addition, efavirenz is used in HIV-patients and pazopanib in patients with metastatic renal cell carcinoma or soft tissue sarcoma – both groups of patients will not be included into the trial

Risk of gastrointestinal ulceration due to Prednisolone

Systemic corticosteroids are not ulcerogenic [37] per se and do not increase the risk of gastrointestinal bleeding[38]. A risk for ulcer complications exists primarily in hospitalized patients[37]. However, this is not the clientele of this study. In combination therapies, increased risk of bleeding complications was shown with the combination of permanent NSARs with corticosteroids, whereas the combination of a Coxib with corticosteroids did not show an increased rate of ulcer bleeding [39]. The use of ASS also increases the risk of gastrointestinal ulcus disease. Taking an NSAR or ASS is therefore an exclusion criterion. A primary prophylactic administration of proton pump inhibitors (PPI) is only recommended[40] for patients with long-term use of NSARs and additional risk factors[41].

In PreVitaCOV, patients on permanent NSAR (or anticoagulation) are excluded from the study. (Protocol, Section 9.2). For further safety of our patients, we have established a safety procedure detailed in section 10.11 of this protocol.

Risk of impaired glucose tolerance and blood pressure due to Prednisolone

Increased blood sugar and blood pressure occurs mainly after long-term use of higher doses of corticosteroids and is reversible[42]. In patients treated with prednisolone, increase of blood glucose can occur up to 2% in primary care patients[43] and is associated with dose and duration of treatment, older age and body mass index a.o. [44] In our study, only a moderate dose will be given for the short duration of 5 days and the low dose prednisolone will be administered for 23 days, resulting in a very low risk for manifestation or exacerbation of an impaired glucose tolerance or diabetes mellitus. In clinical practice, elevated values do not lead to a cessation of the treatment, because these changes are reversible. Baseline data of blood pressure/glucose and a control at the end of the intervention would allow for the detection elevated values for both. According to clinical practice, patients with pathological values are then referred to their general practitioner for further diagnostic and treatment if required.

If impaired glucose tolerance is suspected during the baseline visit (blood glucose ≥ 126 mg/dl fasting or ≥ 200 mg/dl), the patient is referred to his/her general practitioner for evaluation. Representation will be possible after clarification.

In the treatment of several diseases, cortisone pulse therapy is a common treatment method. E.g., the guidelines for the treatment facial paresis does not include recommendations for monitoring blood pressure or blood glucose in healthy individuals.[45] We will test blood pressure and blood sugar at the start and at the beginning of the treatment as recommended for clinical trials.[46]

Furthermore, we will provide each patient with a CE-certified blood pressure monitor on the day of baseline.

The patient should check their blood pressure daily for the first seven days of taking the study medication. The measurement should be performed according to current recommendations for self-measurement. This will be explained to the patient on the day of the baseline visit and can be found on the leaflet provided regarding medication intake and further investigations.

The values should be noted in the patient diary. The aim of the measure is to prevent/detect hypertensive derailment at an early stage.

- In case of multiple readings > 140/90 mmHg in patients without pre-existing arterial hypertension, patients should attend a check-up at their general practitioner's office in the following two to three weeks.
- In case of multiple readings > 160/90 mmHg, patients should attend a near-term check-up at their primary care physician's office in the following one to two days, if the values do not correspond to their normal level.
- If values ≥ 180/90 mmHg occur and persist even after repeated measurement under optimized conditions (see leaflet on medication and subsequent examinations), immediate medical consultation is requested.

Discontinuation of study medication is to be considered as a case-by-case decision based on the medical assessment of the responsible physician.

On the day of the second visit to the study centre, patients are required to return the blood pressure monitor. The study nurses perform the evaluation and data transfer from the patient diary. In the eCRF, it is only noted whether a regular blood pressure check was carried out as ordered and whether any abnormalities occurred.

6.4 Patient involvement

A patient advisory board consisting of six patients with PC19S or who live with an affected person in their household (two patients from each site) will be established and involved in the preparation and implementation of the study via videoconference as demonstrated in a previous trial[5, 6, 9]. It will meet every three or six months. Regarding the initial considerations of the study design, there was no inclusion of individuals suffering from Post-COVID-19 syndrome. However, in the course of the study development, the study has already been presented twice in the already established citizens' forum in Würzburg (Bürgerforum) [47, 48]. In the citizens' forum, scientific projects are discussed with citizens about 4 times a year. Concerning PreVitaCOV, the participants were asked about their opinion on the feasibility and practicability of the proposed procedure. Furthermore, various information materials were presented and discussed with regard to their comprehensibility. The feedback and the opinion of the participants led to the revision of the study procedure, the information flyer, the patient information, as well as the patient diary. Another presentation is planned at the end of the study to inform the participants of the citizens' forum about the further course of the study.

Gender Aspects

According to existing studies, women's risk of developing PC19S seems to be double that of men among patients aged between 40 and 50. After the age of 60 the risk level of long-term COVID between male and female seems to be similar.[49] However, we will take the gender aspect into account by a recruitment strategy that prevents unequal distributions. In the data analyses we will pay particular attention to gender differences concerning symptoms and gender-specific treatment effects. As a standard procedure in clinical trials pregnant women or female patients considering to get pregnant during the trial and within 1 week after the last dose of trial medication are not eligible, negative pregnancy test during the first visit is a condition of enrolment. Women getting pregnant while receiving trial medications will stop trial medication and will receive special attention detailed below.

7 Trial objectives

7.1 Primary, secondary and safety endpoints

The trial is divided into two phases:

In a pilot study we will evaluate feasibility and safety of treating PC19S with Prednisolone and/or Vitamin B1, B6, and B12 in comparison to placebo in patients recruited by their GPs.

1. The primary outcome of the Pilot study will be feasibility and acceptance of screening and recruitment in primary care, as assessed by the retention rate at day 28.

If the pilot phase shows feasibility, the study will be transferred into a confirmatory study to determine the effectiveness of the interventions.

1. The primary outcome of the confirmatory study will be the change in symptom severity to day 28 as assessed by a specifically tailored total score based on the patient reported outcome measurement information system (PROMIS).

Secondary endpoints will be:

1. Severity of each PC19 symptom (PROMIS [50] total and sub scores in the domains included in the total score, MYMOP [51]; PC19S functional status [52]; PC19 symptom list)
2. Health related quality of life (EQ-5D-5L [53] and visual analogue scale)
3. Depression (PHQ-8) [54]
4. Fatigue (Chalder Scale) [55]
5. Pain (Numeric rating scale for pain)
6. Cognitive function: Alertness, distractibility, divided attention, flexibility and visual scanning (TAP) [56]
7. Physical exercise (1minute Sit-to-Stand-Test) [57]
8. Use of on-demand medication and change in concomitant medication (patient diary)
9. feasibility and acceptance (qualitative interviews with subgroup sample; questionnaire)

Safety will be assessed by AE and SAE. In addition, number of patients with ongoing/worsening symptoms: symptoms at the same level or worse two months after inclusion.

8 Description of the trial

8.1 Overall trial design

This is a multicenter, randomized, placebo controlled, double-blind phase III trial with four parallel groups.

8.2 Flow Chart

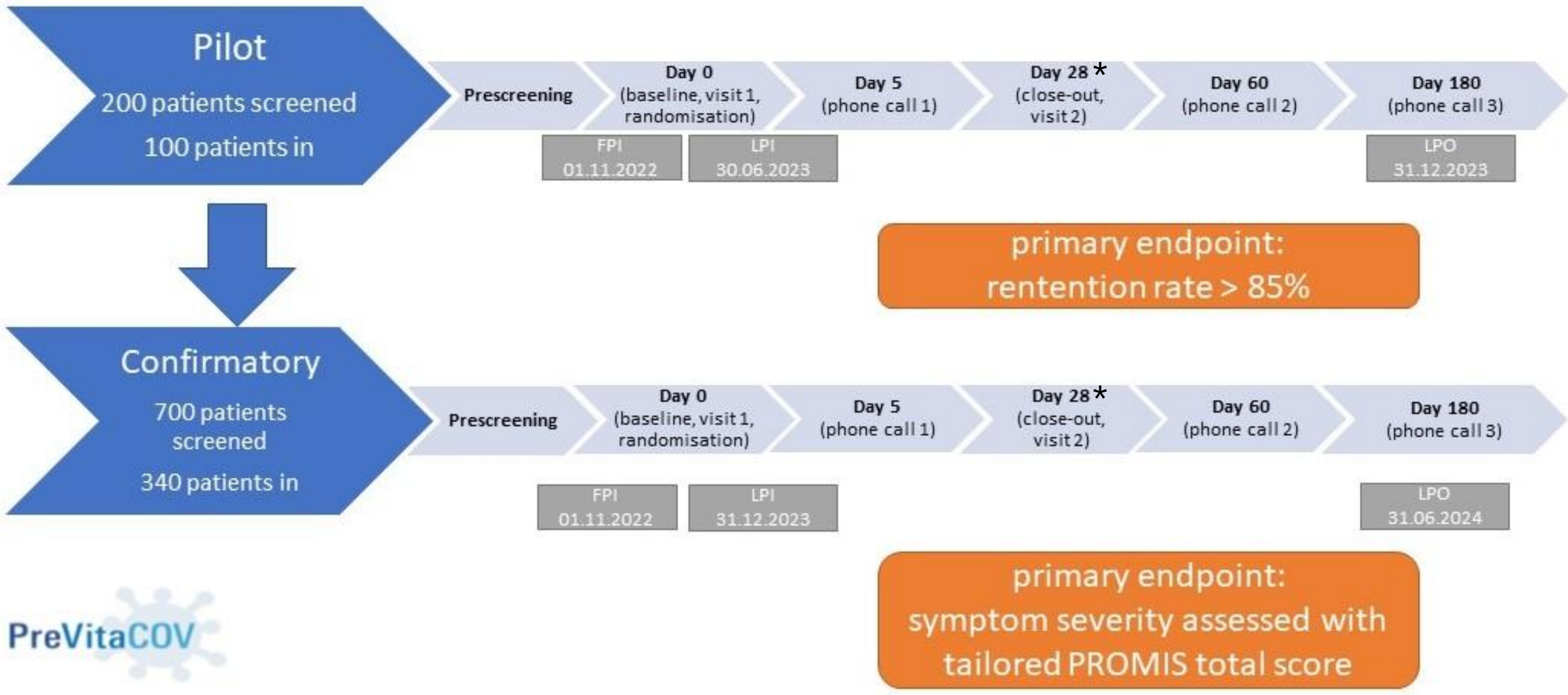

* for women with childbearing potential an additional pregnancy test is performed on day 32 (+ 2)

8.3 Primary outcome measures

The primary endpoint of **the pilot study** is the retention rate measured as the proportion of patients who completed the 28 days intervention.

The primary outcome measure of **the confirmatory study** is the change in the specifically tailored PROMIS total score from baseline to day 28 (visit 2). The score is a weighted mean composed by the sub scores of five domains (fatigue, dyspnoea, cognition, anxiety, depression) relating to typical symptom areas of a PC19S.

Composition and construct of the primary endpoint:

With the primary outcome we aimed to develop a tailored measure of disease burden for patients with Post-COVID-19-Syndrome. This should be a patient reported outcome not too burdensome for patients. We operationalized this as a mean of the T-scores from 5 PROMIS® Short Form instruments: Fatigue 4a, Dyspnoea Severity 10a, Cognitive Function 4a, Emotional Distress-Anxiety 4a and Emotional Distress-Depression 4a.

Because we must distinguish between negatively formulated concepts such as Fatigue, Dyspnoea, Anxiety and Depression and positively formulated concepts such as Cognitive Function, the outcome measure is not a simple mean. Rather, it is a linear combination with the 4 T-scores for the 4 PROMIS Short Forms Fatigue, Dyspnoea Severity, Anxiety, Depression are summed and the T-score for Cognitive Function is subtracted from this subtotal.

8.4 Secondary outcome measures

The secondary outcome measures for **the pilot study** will be

1. severity of PC19S (tailored PROMIS total sore, PROMIS subscores, MYMOP and PC19S functional status (PCFS - Scale), symptom list
2. health related quality of life (EQ-5D-5L and visual analogue scale)
3. depression (PHQ 8)
4. fatigue (Chalder fatigue Scale)
5. numeric rating scale for pain
6. alertness, divided attention, distractibility, flexibility and visual scanning (TAP)[†]
7. physical fitness (1 Minute Sit-to-Stand-Test)
8. use of on-demand medication and change in concomitant medication (patient diary)
9. feasibility and acceptance (qualitative interviews with a subgroup sample, questionnaire)
10. physical examination (auscultation of chest and heart, orientating neurological examination, lymph node status)

Secondary outcome measures for **the confirmatory study** will be

1. severity of PC19S (PROMIS sub scores, MYMOP and PC19S functional status (PCFS - Scale), symptom list
2. health related quality of life (EQ-5D-5L and visual analogue scale)
3. depression (PHQ 8)
4. fatigue (Chalder fatigue Scale)
5. numeric rating scale for pain
6. alertness, divided attention, distractibility, flexibility and visual scanning (TAP)
7. physical fitness (1 Minute Sit-to-Stand-Test)
8. use of on-demand medication and change in concomitant medication (patient diary)
9. feasibility and acceptance (self-developed questionnaire)
10. physical examination (auscultation of chest and heart, orientating neurological examination lymph node status)

Safety outcomes will be adverse events and severe adverse events for both phases as well as the number of patients with worsening PC19 symptoms until day 60.

[†] https://www.psytest.net/de/testbatterien/tap-m/zielsetzung

In addition to the retention rate, feasibility and acceptance of the study procedures will be evaluated by semi-structured interviews with a subgroup sample (n=5-7 patients in each trial site) and a self-developed questionnaire.

8.5 Validation of the outcomes for the elderly

The data collection in this study is very comprehensive and includes individual complaints in addition to the standardised survey of symptoms.

Most of the instruments used are validated in the elderly population, in particular the Montreal Cognitive Assessment (MoCA), which is mainly used in older people, the EQ-5D-5L, the PHQ 8 and the sit-to-stand-Test. For the TAP, which tests attention, distractability and alertness age-adapted reference values are available (cf. table 1).

Using the MoCA and the TAP we carry out an intensive evaluation of cognition, which in case of impairment has serious consequences especially in older people.

Therefore, an extension of the survey module is not necessary from our point of view.

Table 1: Outcomes of the PreVitaCOV-study and their validation for the elderly

Outcomes	Validation for the elderly? Comments and References
**PROMIS total score**<br>Score is a weighted mean of five domain sub scores (fatigue, dyspnoea, cognition, anxiety, depression) relating to typical symptom areas of PC19S.	**„Most PROMIS-PF measures demonstrate sufficient content validity in healthy seniors and various disease populations. "**<br>Zonjee, V. J., Abma, I. L., de Mooij, M. J., van Schaik, S. M., den Berg-Vos, V., Roorda, L. D., & Terwee, C. B. (2022). The patient-reported outcomes measurement information systems (PROMIS®) physical function and its derivative measures in adults: a systematic review of content validity. *Quality of Life Research*, 1-14.
**MoCA**	**Validated for the elderly (55-85 years)**<br>Nasreddine, Z. S., Phillips, N. A., Bédirian, V., Charbonneau, S., Whitehead, V., Collin, I., & Chertkow, H. (2005). The Montreal Cognitive Assessment, MoCA: a brief screening tool for mild cognitive impairment. *Journal of the American Geriatrics Society*, *53*(4), 695-699.
**MYMOP**	**The pictorial version was conceptualised for the elderly.**<br>Ishaque, S., Johnson, J. A., & Vohra, S. (2019). Individualized health-related quality of life instrument Measure Yourself Medical Outcome Profile (MYMOP) and its adaptations: a critical appraisal. *Quality of Life Research*, *28*(4), 879-893.<br>Paterson, C. (1996). Measuring outcomes in primary care: a patient generated measure, MYMOP, compared with the SF-36 health survey. *Bmj*, *312*(7037), 1016-1020.
**PCFS – Scale**	**Construct validity in adults (46 ± 11 years); due to the novelty of the topic there is no validation study for the elderly yet.**<br>Machado, F. V., Meys, R., Delbressine, J. M., Vaes, A. W., Goërtz, Y. M., van Herck, M., ... & Spruit, M. A. (2021). Construct validity of the Post-COVID-19 Functional Status Scale in adult subjects with COVID-19. *Health and quality of life outcomes*, *19*(1), 1-10.

**EQ-5D-5L and visual analogue scale**	**Validated for the elderly.**<br>Penton, H., Dayson, C., Hulme, C., & Young, T. (2022). A Qualitative Investigation of Older Adults' Conceptualization of Quality of Life and a Think-Aloud Content Validation of the EQ-5D-5L, SF-12v2, Warwick Edinburgh Mental Wellbeing Scale, and Office of National Statistics-4. *Value in Health*.<br>Bhadhuri, A., Kind, P., Salari, P., Jungo, K. T., Boland, B., Byrne, S., & Schwenkglenks, M. (2020). Measurement properties of EQ-5D-3L and EQ-5D-5L in recording self-reported health status in older patients with substantial multimorbidity and polypharmacy. *Health and quality of life outcomes*, *18*(1), 1-17.
**PHQ-8**	**We use the PHQ 9, omitting the last question concerning the wish to be dead. The PHQ-8 and PHQ-9 are comparable in screening results.**<br>Lamers, F., Jonkers, C. C., Bosma, H., Penninx, B. W., Knottnerus, J. A., & van Eijk, J. T. M. (2008). Summed score of the Patient Health Questionnaire-9 was a reliable and valid method for depression screening in chronically ill elderly patients. *Journal of clinical epidemiology*, *61*(7), 679-687.
**Chalder Fatigue Scale**	**Validated for adults, not specifically for the elderly.**<br>Shahid, A., Wilkinson, K., Marcu, S., & Shapiro, C. M. (2011). Chalder fatigue scale. In *STOP, THAT and One Hundred Other Sleep Scales* (pp. 97-98). Springer, New York, NY.<br>Fong, T. C., Chan, J. S., Chan, C. L., Ho, R. T., Ziea, E. T., Wong, V. C., ... & Ng, S. M. (2015). Psychometric properties of the Chalder Fatigue Scale revisited: an exploratory structural equation modeling approach. *Quality of Life Research*, *24*(9), 2273-2278.
**TAP** (Test Alertness, divided attention, distractibility, optional flexibility and visual scanning)	**Age-specific norm values are available.**<br>https://www.psytest.net/de/testbatterien/tap-m/zielsetzung<br>**General validation: [56]**<br>Zimmermann, P., & Fimm, B. (2002). A test battery for attentional performance. Applied neuropsychology of attention. Theory, diagnosis and rehabilitation, 110-151.
**1min-Sit-to-Stand-Test**	**Validated for the elderly.**<br>Ritchie, C., Trost, S. G., Brown, W., & Armit, C. (2005). Reliability and validity of physical fitness field tests for adults aged 55 to 70 years. *Journal of Science and Medicine in Sport*, *8*(1), 61-70.<br>Bohannon, R. W., & Crouch, R. (2019). 1-Minute sit-to-stand test: systematic review of procedures, performance, and clinimetric properties. *Journal of cardiopulmonary rehabilitation and prevention*, *39*(1), 2-8.<br>Strassmann, A., Steurer-Stey, C., Lana, K.D. et al. Population-based reference values for the 1-min sit-to-stand test. Int J Public Health 2013; 58:949–953.

8.6 Sample size

Pilot study:

A total of 100 patients will be enrolled for the pilot study. When this stage indicates feasibility (defined as a retention rate of at least 85% until day 28 or according to a recommendation of the DSMB), the trial will be transformed into a confirmatory study to investigate effectiveness of the treatments.

The aim of the sample size calculation for the pilot study was to ensure that we could estimate the retention rate - that means proportion of patients who completed the 4-week intervention, with sufficient precision. Sufficient precision here means that the width of the corresponding 95% confidence interval not exceeds 0.15 (15%). Conservatively we base the calculation on the exact (Clopper-Paerson) method. Furthermore, because the target retention rate in this trial is 0.85 (85%) we assume for the calculation an observed sample proportion of 0.85. Then 100 patients will be needed to yield a confidence interval with the postulated precision.

According to the study design 4 equal sized treatment groups are planned. Therefore 4*25 = 100 patients are to be randomized and analysed in a 1:1:1:1 ratio at the beginning. This means in turn that n=200 are to be assessed for eligibility and n=100 are to be allocated to trial

The observed retention rate will be one component within the criteria for continuing to the confirmatory study, the confirmatory part comparing the effectiveness of treatments. For example, the sample size of 100 allows an underlying retention rate of 0.85, which is considered as an acceptable retention rate here, to be distinguished from an underlying retention rate of 0.75 with power 78% at a significance level 0.05 and from an underlying retention rate of 0.73 with power of 90%.

We do not want to base the decision of whether to continue with the confirmatory study exclusively on a formal statistical criterion, so we calculated the sample size for the pilot study based on the precision of the estimate, not on a procedure of sequential testing.

Confirmatory study:

In accordance with the extension of the “CONSORT 2010 statement to randomized pilot and feasibility trials”[58] the primary endpoint of the confirmatory study is defined as severity of PC19S measured by the tailored PROMIS total score. This score is composed of sub scores for five typical symptom domains. The corresponding primary outcome measure is the change in this score from day 0 to day 28. Therefore, the calculation of the total sample size needed for both phases, 4*72 = 288 for the primary analysis, was based on the analysis of this outcome measure. Accordingly, 340 are to be allocated to the trial and 700 to be assessed for eligibility in total, whereas the patients of the pilot study are to be included.

8.7 Strategy for transition from pilot study to confirmatory study

An important condition for a subsequent confirmatory study (successfully assessing and comparing the effectiveness of treating PC19S patients with prednisolone and/or vitamin B1, B6 and B12 vitamins, is a sufficient underlying retention rate. For this we assess 0.85 (85%) as a sufficient retention rate. Therefore, an actually observed retention rate based on 100 patients which is not compatible to this requirement would lead to a stop and not passing to the confirmatory study. We assess an observed retention rate < 0.80 (80%) as such an incompatible retention rate. A reasoning for this argumentation is that assuming an observed retention rate of 0.80 the upper limit of the corresponding one-sided upper-limit 90% confidence interval, calculated according to the Wilson-score method, is 0.846 (84.6%). That means this one-sided upper-limit 90% confidence interval would not include the target value 0.85 and with this indicate insufficient retention. If the retention rate is not achieved, the DSMB may recommend to continue based on the progression of retention (e.g., if retention has been improved in the course of the study, but has formally fallen the 85%).

If the criteria to continue (retention rate or recommendation DSMB) are met, there will be no analysis of secondary endpoints and the study will be transformed into the confirmatory study. **Patients enrolled in the pilot study will be included in the analyses of the confirmatory study.**

According to paragraph 10, section 1, of the German GCP Ordinance, the sponsor will inform both the ethics committee and the competent federal higher authority (BfArM) if any changes to the protocol should be necessary, especially after phase 1 of the study. In addition to that, the sponsor will inform both the ethics committee and the BfArM within 15 days if, during or after phase 1 of the study, the sponsor becomes aware of facts that must be assumed to have changed the benefit-risk-assessment (paragraph 13, section 4, of the German GCP Ordinance). This regards in particular a) reports of expected and severe side effects with unexpected outcome, b) an accumulation of expected and severe side effects that are considered clinically relevant, c) suspected cases of unexpected and severe side effects occurring after the participant had ended the trial, and d) any event related to the trial of preparation of the trial medication that is possibly endangering the safety of participants.

8.8 Timetable

The end of the study will be reached either after the pilot study if the feasibility is not given or if the data safety monitoring board suggests and the sponsor decides to stop the study prematurely. Otherwise, the study ends when 340 patients have been included and completed the follow up phone call 3.

For the individual patient:

The study intervention per patient will take 28 days followed by an observation period of 5 months after the end of study treatment. The individual patient study duration is 6 months.

Pilot study:

The overall pilot study duration (including data analysis) is 18 months. The recruitment period is a maximum of 8 months (from the initiation visit). The recruitment target is 25 patients per treatment arm for the pilot study. The treatment phase is 28 days. Follow-ups take place until 6 months after inclusion.

First patient first visit (FPFV): 01.11.2022
Last patient last visit (LPLV): 31.12.2023

Confirmatory study (including the pilot study in case of transformation):

The overall confirmatory study duration (including data analysis) is 24 months. The recruitment period is a maximum of 14 months (from the initiation visit). The recruitment target is 72 patients per treatment arm for the confirmatory study. The treatment phase is 28 days. Follow-ups take place until 6 months after inclusion.

First patient first visit (FPFV): 01.11.2022

Last patient last visit (LPLV): 30.06.2024

9 Trial population

9.1 Inclusion criteria

1. Adult patients.
2. History of SARS-CoV-2 infection at least 12 weeks ago (if patients had more than one infection with SARS-CoV-2 the 12 weeks refer to the infection causing the Post-COVID-19 symptoms); the infection must be documented by either a positive PCR- or Antibody-Test or be confirmed by the patient's GP on the provided sheet to confirm Post-COVID-19 diagnosis, pre-existing conditions, and prior medication.
3. Symptoms concerning at least one of the following domains: fatigue, dyspnea, cognition, anxiety, depression.
4. Symptoms that developed during or after the SARS-CoV-2 infection, that persist until study inclusion and that are associated with COVID-19 (the latter will be assessed by the patient's GP or the local investigator).

9.2 Exclusion criteria

1. Acute COVID-19 at baseline visit (rapid SARS-CoV-2 antigen test).
2. Patients who were treated in the intensive care unit because of COVID-19.
3. Pregnancy/ breastfeeding.
4. Diabetes mellitus
5. PC19S symptoms can be explained by an alternative diagnose (e.g., chronic fatigue syndrome, depression, active or preceding cancer therapy, severe anemia, sleep apnea syndrome) as assessed by the patients' GP or the investigator (responsible physician)
6. <u>History of severe</u> medical conditions such as
    - concomitant acute infectious disease
    - gastrointestinal ulcer
    - liver disease/ liver cirrhosis
    - malabsorption or condition after bariatric surgery
    - chronic airway disease (e.g., asthma, COPD)
    - chronic heart failure (NYHA III or IV)
    - neurologic disorders (e.g., multiple sclerosis, motoneuron disease)
    - untreated hypothyroidism
    - significantly impaired glucuronidation (e.g., Gilbert-Meulengracht, ROTOR, or Crigler-Najjar syndrome)
    - immunodeficiency or a chronically weakened immune system (e.g., HIV, AIDS, lymphoma, chemo-radio-therapy, immunosuppressive pathology)
    - Mental disorders (e.g., depression, psychosis, dementia)

- Active cancer
- any other severe medical conditions that preclude participation as determined by responsible physician

7. current use of
    - immunosuppressive drugs
    - non-steroidal anti-inflammatory drugs (NSAID), ASS, Indometacin
    - fluoroquinolones
    - anticoagulation: phenprocoumon or other cumarin derivates, direct oral anticoagulants
    - any other drug with a possible interaction that could exhibit clinically relevant interactions with the study medication (as described in Fachinformation Prednisolon STADA®, Predni H Tablinen® Zentiva or Fachinformation Vitamin B komplex Hevert). The decision regarding the clinical relevance of the interactions is at the discretion of the clinical investigator.
8. Systemic treatment with prednisolone for at least 7 days since COVID-19 or any parenteral application since the end of the acute phase of COVID-19; treatment with vitamins B1, B6, or B12 in doses equivalent to the dose of the study medication for at least 7 days or any parenteral application since the end of the acute phase of COVID-19; vitamin supplements containing vitamin B1, B6, or B12 should have been ceased at least 4 weeks prior to the inclusion in the study.
9. Known allergy and contraindications to the intervention drugs.
10. Need of care and/or peer dependency
11. Nursing home residents
12. Inability to understand the scope of the study, to follow study procedures and to give informed consent or to attend the study sites.
13. Participation in another interventional trial at the same time or within the past 3 months before enrollment
14. Female patients considering to get pregnant during the trial and within 1 week after the last dose of study drug(s)

9.3 Patient recruitment

A two-step-approach will be pursued for recruiting the patients.

a) First, patients who visit their GP with symptoms of PC19S will be screened for eligibility by GPs in their practices. In addition, GPs will screen patients based on the electronic health records. To support screening, we will provide diagnostic guidance for GPs. Eligible patients will receive a letter of invitation from their GP inviting them to participate and providing the contact address of the trial sites.

b) Second, patients will present at the trial sites upon appointment, equipped with their medical history and concomitant medication provided by their GP on paper. Again, in- and exclusion criteria will be proven carefully by clinicians at the trial site at day 0 (baseline visit) to minimize the risk of false patient inclusion. Further trial management including patients' enrolment, treatment and management will be carried out at the three trial sites. If recruitment rates are low, further recruitment strategies (e.g., social media, press releases) will be considered.

Patient screening by GPs follows a pragmatic approach using their experience in patient assessment in term of eligibility for our trial. These considerations justify that the trial puts the diagnostic decision into the hands of GPs, mimicking a real-life situation.

We expect the guidelines on the diagnostics of PC19S to evolve, and we will provide the GPs working in the trial with an up-to-date guideline on how to diagnose PC19S and how to exclude patients with other diagnoses than PC19S in the GP setting.

Patients who contact one of the sites directly may also be admitted. Ideally, however, a presentation should be made to one of the participating GPs in advance, so that they can collect important medical data (such as medical history, concomitant medication) and forward them to the site. Basically, recruitment via the GPs is to be preferred for reasons of standardization.

10 Trial medication

10.1 Trial drug 1: Prednisolone

The dosage will be Prednisolone 20 mg for five days and subsequently 5 mg for 23 days (both 1x1 capsule / day).
Trial drug 1 will be distributed by the Hospital Pharmacy of the University Hospital Hamburg-Eppendorf, Gebäude Nord 19, Martinistraße 52, 20246. Prednisolone 5 mg and 10 mg tablets will be encapsulated and packed into a box containing 5 capsules of Prednisolone 20 mg (20 mg = 2 tablets 10 mg/capsule), and a second box containing 28 capsules of Prednisolone 5 mg (5 mg = 1 tablet 5 mg/ capsule). Storage will require a room temperature ≤ 25°C. The boxes will be labelled in a blinded manner as an investigational medicinal product (IMP) by the Hospital Pharmacy. Shelf life of the pharmaceutical form is 12 months.

For further details see Investigational Medicinal Product Dossier (IMPD) of the pharmacy and Summary of Product Characteristics (SmPC) (Fachinformation) Prednisolon STADA®, Predni H Tablinen® Zentiva .

10.2 Trial drug 2: Vitamin B compound

Trial drug 2 will be provided by Hevert-Arzneimittel GmbH, In der Weiherwiese 1, 55569 Nussbaum in bulk blister à 20 filmcoated tablets. Two blisters will be packaged in folding boxes by the Hospital Pharmacy of the University Hospital Hamburg. Blister and folding boxes will be labelled in a blinded manner as an IMP. The shelf life of the bulk corresponds to the approval, i.e., 3 years. The shelf life of the IMP is blinded and adjusted to the shelf life of the placebo tablets. The initial shelf life of placebo is 12 months and will be extended up to 35 months depending on further stability data, attached by Hevert, for the placebo tablets.

For further details see IMPD of the pharmacy and SmPC (Fachinformation) Vitamin B Komplex forte Hevert.

10.3 Placebo for Prednisolone

The dosage will be Placebo-Prednisolone 20 mg for five days and subsequently Placebo-Prednisolone 5 mg for 23 days (both 1x1 capsule/ day)
Trial drug 3 will be distributed by the Hospital Pharmacy of the University Hospital Hamburg-Eppendorf, Gebäude Nord 19, Martinistraße 52, 20246 Hamburg. P-Tabletten 8 mm weiß Lichtenstein® will be encapsulated (Placebo-Prednisolone 20 mg = 4 tablets/capsule and Placebo-Prednisolone 5 mg = 1 tablet/capsule) and packed into a box containing 5 capsules of Placebo-Prednisolone 20 mg and a second box containing 28 capsules of Placebo-Prednisolone 5 mg. Storage will require a room temperature ≤25°C. The boxes will be labelled in a blinded manner as an IMP by the Hospital Pharmacy. Shelf life of the pharmaceutical form is 24 months, the shelf life of the IMP is blinded and adjusted to the shelf life of the Verum capsules.

For further details see IMPD of the pharmacy and SmPC (Fachinformation) P-Tabletten 8 mm weiß Lichtenstein

10.4 Placebo for Vitamin B compound

Trial drug 4 will be provided by Hevert-Arzneimittel GmbH, In der Weiherwiese 1, D-55569 Nussbaum in bulk blister à 20 filmcoated tablets. Two blisters will be packaged in folding boxes by the Hospital Pharmacy of the University Hospital Hamburg-Eppendorf. Blister and boxes will be labelled in a blinded manner as an IMP. Shelf life corresponds to 1.2 Trial drug 2: Vitamin B

For further details see IMPD of the pharmacy.

10.5 Packaging and labeling

Packaging and labelling of the IMPs will be carried out by the Hospital Pharmacy of the University Hospital Hamburg. The Hospital Pharmacy of the University Hospital Hamburg-Eppendorf holds a manufacture's license pursuant §13 German Medicines Act (AMG) for the production of IMPs. The labelling will take place pursuant to Annex VI Clinical Trial Regulation 536/2014 (CTR).

The following investigational medications will be given to the patient:

- One box with 5 capsules of prednisolone 20 mg or placebo
- One box with 28 capsules of prednisolone 5 mg or placebo
- One folding box with 2 blisters, 20 tablets each, of Vitamin B compound or placebo.

Fertigarzneimittel Prednisolon 20 mg:
Kapselbox Nr. 1/2
Dateiname:
KP.Eti.Box1.RA-PreVitaCov.Zwf.3664.dotx

NUR ZUR KLINISCHEN PRÜFUNG BESTIMMT
Sponsor: Universitätsklinikum Würzburg, Josef-Schneider-Str. 2/D7, 97080 Würzburg,
Hauptansprechpartner: Prof. Dr. med. Ildikó Gágyor, Tel. +49 (0) 931 201-47802
Prüfplancode: F001AM0222_1 EudraCT-Nr: 2022-001041-20 **Dose 1/2**
Studien-ID: ___________ **Random.-Nr.: 000000**
Kapseln zum Einnehmen Inhalt: 5 Kapseln
1 Kapsel enthält Prednisolon 20 mg oder Placebo. Enthält Lactose.
Dosierung: nach Anweisung durch den Prüfarzt.
Ch.-B.: RA-K20/00 Verwendbar bis: 00.00.0000
Dose trocken, vor Licht geschützt und dicht verschlossen aufbewahren.
Nicht über 25°C lagern.
Für Kinder unzugänglich aufbewahren.
Klinikapotheke UKE, Martinistraße 52, 20246 Hamburg, Tel.: +49 (0) 40 7410 56239

Fertigarzneimittel Prednisolon 5 mg:
Kapselbox Nr. 2/2
Dateiname:
KP.Eti.Box2.RA-PreVitaCov.Zwf.3664.dotx

NUR ZUR KLINISCHEN PRÜFUNG BESTIMMT
Sponsor: Universitätsklinikum Würzburg, Josef-Schneider-Str. 2/D7, 97080 Würzburg,
Hauptansprechpartner: Prof. Dr. med. Ildikó Gágyor, Tel. +49 (0) 931 201-47802
Prüfplancode: F001AM0222_1 EudraCT-Nr: 2022-001041-20 **Dose 2/2**
Studien-ID: ___________ **Random.-Nr.: 000000**
Kapseln zum Einnehmen Inhalt: 28 Kapseln
1 Kapsel enthält Prednisolon 5 mg oder Placebo. Enthält Lactose
Dosierung: nach Anweisung durch den Prüfarzt.
Ch.-B.: RA-K5/00 Verwendbar bis: 00.00.0000
Dose trocken, vor Licht geschützt und dicht verschlossen aufbewahren.
Nicht über 25°C lagern.
Für Kinder unzugänglich aufbewahren.
Klinikapotheke UKE, Martinistraße 52, 20246 Hamburg, Tel.: +49 (0) 40 7410 56239

Fertigarzneimittel Vitamin B Komplex:
Faltkarton
Dateiname:
KP.Eti.Karton.RA-PreVitaCov.Zwf.3475.dotx

NUR ZUR KLINISCHEN PRÜFUNG BESTIMMT
Sponsor: Universitätsklinikum Würzburg, Josef-Schneider-Str. 2/D7,
97080 Würzburg,
Hauptansprechpartner: Prof. Dr. med. Ildikó Gágyor, Tel. +49 (0) 931 201-47802
Prüfplancode: F001AM0222_1 EudraCT-Nr: 2022-001041-20
Studien-ID: ______________ **Random.-Nr.: 000000**
Tabletten zum Einnehmen Inhalt: 40 Tabletten
1 Tablette enthält Vitamin B Komplex aus 100 mg Vitamin B1 (Thiaminnitrat), 50 mg Vitamin B6 (Pyridoxinhydrochlorid), 500 µg Vitamin B12 (Cyanocobalamin) oder Placebo.
Dosierung: nach Anweisung durch den Prüfarzt.
Vor Licht geschützt und nicht über 25°C lagern. Packung trocken und verschlossen aufbewahren. Für Kinder unzugänglich aufbewahren.
Ch.-B.: RA-T/00 Verwendbar bis: 00.00.0000
Klinikapotheke UKE, Martinistraße 52, 20246 Hamburg, Tel.: +49 (0) 40 7410 56239

Fertigarzneimittel Vitamin B Komplex:
Blister
Dateiname:
KP.Eti.Blister.RA-PreVitaCov.Zwf.3666.dotx

Sponsor: Universitätsklinikum Würzburg, Josef-Schneider-Str. 2/D7,
97080 Würzburg, Prof. Dr. med. Ildikó Gágyor
Prüfplancode: F001AM0222_1
EudraCT-Nr: 2022-001041-20
Studien-ID:
Random.-Nr.: 000000
1 Tbl enthält Vitamin B komplex oder Placebo
Verwendbar bis: 00.00.0000
Ch.-B.: RA-T/00

10.6 Storage, release and return and documentation of the trial product (drug accountability)

The trial drugs will be sent by the Hospital Pharmacy of the University Hospital Hamburg- Eppendorf to the three trial sites Würzburg, Tübingen, and Kiel. Written acknowledgement of receipt will be provided by the investigators. Further orders due to the limited shelf life will be placed by the trial center in Würzburg. Replacement of trial medication still at the trial sites will be carried out by the trial center in Würzburg. Residual stocks after completion of the recruitment phase will be destroyed by the hospital pharmacies of the trial sites.

The PIs will also be responsible for making the storage site inaccessible to unauthorized individuals, so that only the investigators and authorized personnel have access. The personnel with access to the investigational product must be entered by name and area of responsibility on the delegation log.

When patients receive the trial drug, they will be carefully instructed in how to handle and store the medication. This includes being told that any leftover medication and the empty pack must be returned to the trial site.

The dispensing and return of the trial product to the trial site will be documented in the Drug Accountability Log in the investigator site file (identifying the patient, the date and quantity of medication dispensed, the date and quantity of medication returned to the investigator, the date and quantity of medication returned to the study centers). For the destruction of the trial products conducted by the local hospital pharmacy of the trial site, the returned trial products will be send by the PI to the Hospital Pharmacy of the trial sites after the close out visit. A certificate of destruction will be send afterwards to the sponsor.

10.7 Randomization

Patients will be allocated to the 4 treatment groups via stratified block randomization with varying block size. The single stratification factor is the study center. With this method of random treatment assignment, we try to avoid predictability, selection bias and full or partial unblinding still accomplish an acceptable balance. The randomization lists will be generated outside the secuTrial ® and then imported to secuTrial ®. Computerized central randomization and generation of the randomization list recording the code numbers and assignment to the treatments will be carried out by the clinical trial center in Würzburg. The process will be overseen by the trial statistician. The randomization code will be marked on his/her medication and linked with the patient identifier (Pat-ID) within secuTrial ®.

10.8 Treatment schedule

	**Day 0**	**Day 1-5**	**Day 6-28**
**1st arm (prednisolone and placebo)**	enrolment	20 mg prednisolone 1x1 and<br>placebo 1x1	5 mg prednisolone 1x1 and<br>placebo 1x1
**2nd arm (placebo and Vitamin B compound)**	enrolment	placebo 1x1 and<br>Vitamin B compound (100 mg B1, 50 mg B6, 500 µg B12) 1x1	placebo 1x1 and<br>Vitamin B compound (100 mg B1, 50 mg B6, 500 µg B12) 1x1
**3rd arm (prednisolone and Vitamin B compound)**	enrolment	20 mg prednisolone 1x1 and<br>Vitamin B compound (100 mg B1, 50 mg B6, 500 µg B12) 1x1	5 mg prednisolone 1x1 and<br>Vitamin B compound (100 mg B1, 50 mg B6, 500 µg B12) 1x1
**4th arm (placebo and placebo)**	enrolment	placebo 1x1 and<br>placebo 1x1	placebo 1x1 and<br>placebo 1x1

10.9 Administration instructions

Patients will be instructed on how to take the medication. The tablets are swallowed in whole 1x daily with plenty of liquid in the morning.

10.10 Emergency procedures

On enrolment in the study, the patient will receive an emergency card containing information about the study and a telephone number for emergencies for further information and unblinding, respectively.

If hypersensitivity reactions or AEs occur while the trial drug is being taken, the action to be taken is at the discretion of the treating physician. Emergency unblinding should take place only if it is categorically necessary in a medical emergency which requires information about the trial drug.

Any unblinding has to be reported to the Sponsor and to the responsible PI of the respective center without delay.

10.11 Concomitant medication

Patients will be advised to avoid the intake of drugs that may interfere with the trial medication:

- non- steroidal antirheumatic drugs (NSAID, i.e., painkillers like ibuprofen or diclofenac; ASS even in prophylactic anticoagulatory dosage). Patients will be made aware of a potential risk associated with NSAR and ASS, given alternatives for analgesic medication, and instructed to contact his or her general practitioner if they wish to take NSAR or ASS, for example as an analgesic. This information is also provided to the patient in form of a leaflet. If the use of NSARs or ASS becomes necessary for the patient, the treating general practitioner is sensitized to adjust the medication. We will inform him of his patient's possible corticosteroid use at the start of the study. When the patient presents for newly initiated therapy with pain medication, the general practitioner decides on the necessity of therapy with PPI according to individual risk.
- fluoroquinolones
- antacids (magnesium-/aluminiumhydroxid) for at least 2 hours after taking the trial medication
- Any other drug with a possible interaction that could exhibit clinically relevant interactions with the study medication (as described in Fachinformation Prednisolon STADA®, Predni H Tablinen® Zentiva or Fachinformation Vitamin B komplex Hevert). The decision regarding the clinical relevance of the interactions is at the discretion of the clinical investigator.

Patients will also be advised to avoid the intake of the trial medication available as over the counter drugs:

- vitamin supplementation containing vitamin B1, B6, or B12
- prednisolone (not including local application)

Long-term medication will be documented on the eCRF; ingestion should continue as usual. Patients will record in the follow-up survey whether they received any new medical treatment since enrollment in the trial.

Furthermore, apart from oral instruction of the patients, a patient leaflet containing further information concerning the intake of concomitant medication, especially NSAIDs because auf their increased ulcerogenic potential, will be handed out on the first visit at the study center.

10.12 Compliance

Compliance will be checked and documented on day 28 (visit 2) considering the empty pack or any residual medication returned to the trial site.

10.13 Blinding and sealed opaque envelopes

10.13.1 Blinding

This study is designed as a double-blind trial. Therefore, neither patients nor clinicians will know the study arm they are in. This will be achieved by an identical appearance of the tablets containing Vitamin B or placebo and identically encapsulated tablets for prednisolone or placebo (double dummy design).

Blinding and labelling of the trial drugs will be performed by the Hospital Pharmacy of the University Hospital Hamburg in accordance with §13 AMG. The trial drugs will be sent to the trial center in Würzburg and then distributed to the individual trial sites.

10.13.2 Early unblinding

In case where urgent unblinding is necessary, the responsible study center can arrange the unblinding at any time. For this purpose contact information is provided on the patient emergency card.

Early unblinding is performed only in case of medical emergency when it is essential to know the trial medication, i.e., when the physician believes that clinical management depends importantly upon knowledge of whether the patient received prednisolone and / or Vitamin B compound, this also counts for pregnancies. In this case, the responsible study center must inform the sponsor immediately. Urgent unblinding during the PreVitaCOV study is performed by the responsible study center. The responsible physician performs the unblinding of the patient using the eCRF and its unblinding tool. In case of unblinding, the sponsor will be informed automatically. If the eCRF is not available, unblinding must be performed manually using emergency envelopes. In this case, the sponsor needs to be informed by fax +49 (0)931-201-60-39340 and by using the emergency unblinding report form without delay. As soon as the eCRF is available again the unblinding needs to be documented on the eCRF as well.

The emergency and/or the SAE as well as the reason for the early unblinding must be documented on both the eCRF and in case a manually unblinding was performed on the opaque envelope. The trial medication will be stopped but the patient will be monitored by the end of the follow-up.

10.13.2.1 Emergency envelopes

The trial medication will come with a sealed opaque envelope for each medical kit. The envelope will be kept in the investigator' site file. In addition, copies of the envelopes will also be available at the study pharmacy (University Hospital Hamburg) to ensure immediate unblinding in case of emergency.

The following information will be printed on the sealed envelope: address and telephone number of the study centers, randomization code number, trial number, and the statement "To be opened only in an emergency". The sealed opaque envelope will contain the following information: trial medication according to the randomization number; section for the date, time and reason for opening.

The envelope must be opened only if it is necessary to identify the trial medication in an emergency. This must be documented on the eCRF and ISF. The integrity of the envelopes will be checked during monitoring. The sealed opaque envelopes will be collected at the close-out visit.

10.13.3 Regular unblinding

Regular unblinding takes place after completion of data collection and finalization of database and statistical analysis plan.

11 Trial procedures

Data are collected at baseline (day 0; visit 1), at day 5(+3) (phone call 1), at day 14 (patient diary 1), at day 21 (patient diary 2), at day 28(+3) (visit 2), at follow-up after 60(+/- 5) days (phone call 2) and 180 (+/-7) days (phone call 3).

Trial documents

1. trial protocol (this document)
2. ethics vote
3. code book/ biostatistics
4. information sheet for GPs and patients
5. data protection concept
6. informed consent
7. questionnaires
8. patient diary
9. emergency card and follow-up appointments
10. patient leaflet on medication
11. patient leaflets and recruiting material
12. core publication concerning the primary endpoint and intervention effectiveness

11.1 Prescreening

Upon calling the study sites for an appointment for the baseline visit, patients will be asked some prescreening questions in order to check the inclusion and exclusion criteria. This will avoid appointments with patients who are not eligible for the study. Upon this first call patients will also be asked if they would be interested in participating in the interview study.

In case inclusion and exclusion criteria are met there is a possibility for an additional telephone call by a physician. The inclusion and exclusion criteria are checked again and within the framework of the telephone call the patient is trained and informed about the study and the course of the study. If the patient is interested in participating in the study, an appointment is made for an on-site appointment at the study center. A written invitation as well as the patient information is sent to the interested participants in advance by mail or e-mail. They should bring this with them on the day of the baseline visit. At this time there is also the possibility to ask questions before the patients are asked to sign the consent form. This procedure is intended to ensure that patients have sufficient time for consideration.

11.2 Visit 1 (baseline, day 0)

Patients will be informed in detail by the investigator and based on the information sheet about the content and objectives of the trial and will be asked whether they are interested in participating. If they are interested, the inclusion and exclusion criteria will be checked. Once all the patients' questions have been clarified, patients will be asked to sign two copies of the data protection sheet and

informed consent. One copy will then be given to the patients; the second copy will be kept in the investigator site file. If more time for consideration is required by the patient another appointment for conduction of the assessments of visit 1 will be set.

Visit 1:

- Information of the patients,
- Data protection sheet, patient informed consent
- Verification of in- and exclusion criteria,
  - Urine pregnancy test in women need to be assessed by the investigator and must have a sensitivity down to at least 25mlU/ml for human chorionic gonadotrophin (hCG)
- Medical history,
- Concomitant medication and psychoactive substances,
- TAP
- Mental fitness (MoCA test [59]) and physical activity (questionnaire),
- Biosamples (see chapter 11.9) and vital signs (blood test, blood glucose, antibody test for SARS-CoV2, height, weight, O2 saturation, heart rate, blood pressure),
- Physical examination (auscultation of chest and heart, orientating neurological examination, lymph node status)
- Randomization, allocation,
- Questionnaires and tests to collect outcome measures,
- Dispensing of the blinded trial medication with instructions on intake,
- Patient instructions on drug intake, diary and how to prepare for telephone call 1 and visit 2,
- Handing out questionnaire templates for the telephone call on day 5,
- Handing out patient diary,
- Handing out emergency card,
- Documentation of data in the eCRF.

11.3 Phone call 1 (day 5+3)

Patients will be phoned on day 5 (+3 days) to collect data on (selected) outcomes (as far as this can be performed by telephone), on the consumption of concomitant medication and psychoactive substances, check AEs / SAEs as well as drug accountability.

Phone call 1:

- Assessment of selected outcomes
- Assessment of AEs (SAEs are recorded immediately and continuously, independent of visits and phone calls
- Documentation of data in the eCRF

11.4 Patient diary (day 1 to 28)

Patients will complete a diary noting the intake of any medication in addition to their usual daily medication (medication on demand and any change in concomitant medication). Furthermore, the PROMIS short form will be completed on day 14 and 21 by the patients alone. Additionally, blood pressure measurements should be taken from Day 1 to Day 7.

11.5 Visit 2 (day 28+3)

Patients will present again at the study site. They will hand back any study medication not taken. Patients will complete questionnaires and clinical tests to collect the data of outcome measures. Selected laboratory parameters will be reassessed and vital signs will be taken again. Furthermore, concomitant medication, as well as (S)AEs and drug accountability will be checked. Finally, patients will be instructed on the follow-up procedure and be provided with questionnaire templates to support follow-up phone call interviews 2 and 3, 60 days resp. 6 months (180 days) after baseline.

Visit 2:

- Biosamples (see chapter 11.9) and vital signs (blood test, blood glucose, weight, O2 saturation, heart rate, blood pressure)
- Physical examination (auscultation of chest and heart, orientating neurological examination lymph node status)
- Assessment of outcomes
- Check of AEs (SAEs are recorded immediately and continuously, independent of visits and phone calls
- Assessment of additional therapies during the study period (e.g., rehabilitation, physiotherapy, complementary medicine, others)
- Drug accountability (Number of capsules and tablets left per box and day)
- Patient instructions regarding follow-up, how to prepare for telephone call 2 and 3, Handing out questionnaire templates for the telephone calls 2 and 3
- Documentation of data in the eCRF (including patient diary)

Handing out and instructions on the use of a pregnancy test (sensitivity at least 25mIU/ml for hCG) to all female participants of childbearing potential to perform on their own responsibility at the earliest on the 4th day (day 32 + 2) after the end of medication intake.
Patients are instructed to contact the study center to report the test result after the test. If the

patient does not call on her own, the study center will contact her the day after to ask for the test result, no later than 3 days after testing.

11.6 Phone call 2 (day 60+/-5)

Phone call 2:

- Assessment of selected outcomes supported by questionnaire templates
- Check of AEs (SAEs are recorded immediately and continuously, independent of visits and phone call).
- Assessment of additional therapies during the study period (e.g., rehabilitation, physiotherapy, complementary medicine, others)
- Patient instructions regarding final follow-up phone call
- Documentation of data on the eCRF

11.7 Phone call 3 (day 180+/-7)

Phone call 3:

- Assessment of selected outcomes supported by questionnaire templates
- Documentation of data on the eCRF
- Assessment of additional therapies during the study period (e.g., rehabilitation, physiotherapy, complementary medicine, others)

  Questionnaire on feasibility and acceptance of the study (postal return to avoid distortion due to effect of social desirability)

11.8 Assessment of safety

The following safety endpoints will be measured in the study:

- number of patients with worsened symptoms based on the PROMIS-Score at 2 months after inclusion
- number of AE and SAE by system organ class, day 0-2 months
- proportion of patients with at least 1 AE, day 0-2 months

The number of AEs/SAEs will be documented on the eCRF on the basis of the data collected at the trial site. In the event of the cumulative occurrence of AE or SAE, the DSMB will then recommend to the sponsor whether to continue or discontinue the trial. In case of a drug related AE/SAE the trial drug should be discontinued.

11.9 Biosamples (Laboratory tests)

Biosamples will be collected at baseline (day 0) and at visit 2 (day 28) and all results will be documented in the eCRF.

After enrollment in the study at baseline (Visit 1)	- conventional urine pregnancy test (sensitivity down to at least 25mlU/ml for human chorionic gonadotrophin (hCG)<br><br>- Antibody test for SARS-CoV2 (anti-nucleocapsid)<br>- Glutamat-Oxalacetat-Transaminase (GOT/ASAT)<br>- Glutamat-Pyruvat-Transaminase (GPT/ALAT)<br>- Gamma-Glutamyltransferase (GGT)<br>- Creatinine and GFR<br>- Thyroid-stimulating-hormone (TSH)<br>- Potassium<br>- Sodium<br>- Calcium<br>- Non-fasting glucose<br>- Regular and differential blood count<br>- Bilirubin<br>- Cortisol level
	- Vitamin B12 status (Homocystein, MMA, HoloTC)<br>- cytokines
At visit 2 (day 28 + 3)	- Non-fasting glucose
	- Vitamin B12 status (Homocystein, MMA, HoloTC)<br>- cytokines
Day 32 + 2	- Conventional urine pregnancy test (sensitivity down to at least 25mlU/ml for human chorionic gonadotrophin (hCG)

Routine and safety analyses as well as pre-analytics including storage of blood samples will be carried out by the hospital laboratories of each trial site, except for cortisol-levels and SARS-CoV2-antibodies. For a better comparison, these parameters will be analyzed in the central laboratory of the university hospital in Tübingen Therefore, the study centers will store a tube of each patients baseline blood sample at – 40 °C or below until. The samples will be sent from Würzburg and Kiel to Tübingen to be analysed. Using pseudonyms, so the laboratory in Tübingen will not know the patient names.

11.9.1 <u>Routine analyses</u>:

Incidental findings of pathological parameters could occur. The investigator of the trial site decides whether there is a reportable finding based on medical considerations. The patient was informed about the possibility of incidental findings before the inclusion in study. Patients have the opportunity to claim their right not to know about the incidental finding. If patients decide not to be informed about possible findings, this will be noted in the eCRF. If the investigator considers a finding to be in need of a follow-up examination or medical intervention and if the patient agreed on getting informed, the

investigator will immediately inform the patient about the findings. If patients agree, the information will also be sent to the GP of the patient.

<u>11.9.2 Evaluation of vitamin B12 status and cytokine measurements:</u>

For evaluation of the vitamin B12 status (measurement of serum concentrations of vitamin B12, holotranscobalamine, homocysteine and methylmalonic acid), and cytokine measurements, analyses and quality controls will be carried out by the Medical School Rüdersdorf of the University of Brandenburg. Levels of interferones α, β, γ, λ1 and λ2/3, interleukin 1β, 6, 8, 10, 12, Tumor Necrosis Factor Alpha (TNF-α), and Recombinant Human Granulocyte Macrophage Colony-Stimulating Factor (GM-CSF) will be measured using a multiplex cytokine/chemokine array.

<table>
<tr><th></th><th>Minimum required amount</th><th>Examples of samples collection systems</th></tr>
<tr><td>Homocysteine</td><td>3 ml blood in special collecting tube</td><td>S-Monovette® HCY-Z-Gel Sarstedt</td></tr>
<tr><td>Vitamin B12 (cobalamine)</td><td>1 ml serum</td><td rowspan="3">9 ml Serum-Monovette</td></tr>
<tr><td>Holotranscobalamine (HoloTC)</td><td>1 ml serum</td></tr>
<tr><td>Methylmalonic acid (MMA)</td><td>1 ml serum</td></tr>
<tr><td>Cytokine measurements
• Interferones α, β, γ, λ1 and λ2/3
• Interleukin 1β, 6, 8, 10, 12
• TNF-α
• GM-CSF</td><td></td><td>9 ml Serum-Monovette</td></tr>
</table>

Blood samples will be centrifuged immediately after collection. Minimum centrifugation time is:

Relative centrifugal acceleration (g)	2000 x g	2500 x g	3000 x g	3500 x g	4000 x g
Serum-Monovette	10 min	10 min	6 min	4 min	4 min
Serum-Gel-Monovette	15 min	10 min	4 min	4 min	4 min

Serum (supernatant) will be transferred into fresh tubes (separate aliquots for vitamin B12-diagnostics and cytokine concentration determinations) and stored at -20 °C. The procedure is described in detail as attached in an appropriate standard operating procedure (SOP).

After the last patient has completed the study, the samples packed on dry ice are to be shipped by courier to the Brandenburg Medical School, Institute of Clinical Pharmacology. The shipment should be pre-arranged and should take place at the beginning of a week to ensure arrival before the weekend. The shipping address is: *Prof. Oliver Zolk, Immanuel Klinik Rüdersdorf, Institute for Clinical Pharmacology, Building Poliklinik Room 313, Seebad 82/83, 15562 Rüdersdorf (Tel. +49 (0)33638 83991, Email: oliver.zolk@mhb-fontane.de)*.

11.10 Sub study *Patients' perspectives and needs concerning care of Post-COVID-19*

A sample of five to seven participants of each trial site will be interviewed, based on a semi-structured interview-questionnaire. Questions will focus on patients' perception of health care and their needs regarding health care. Main objectives are a better understanding of current Post-COVID care, how it is perceived by patients, and to identify approaches to improve care of these patients.

11.10.1 Operationalization of interviews

Patients who are interested in the main study will receive a written information along with the contact information of the local trial center. All patients calling the trial center for an appointment for baseline visit 1 of the main study will be asked if they would also like to participate in the additional interview-study. It will be emphasized that participation is voluntary.

Patients who are interested in participating in the interview-study will be send additional written information concerning this sub-study. Patients will get the opportunity to speak to a trial physician about the benefits and risks of the study. Patients will be asked for their written informed consent which will be returned to the trial site in Tübingen in a prepaid envelope.

They will then receive a call via a conference platform (e.g., Zoom®). Interviews will take between 20 to 40 minutes and the audio will be recorded via the conference tool. The audio-recordings will be stored on the server of the university hospital Tübingen behind a fire wall until transcription. After transcription, the recordings will be destroyed (deleted from the server). Transcripts will be pseudonymized.

The same pseudonyms will be used in the main study to identify the interviews and to be able to link the interview with sociodemographic data so that patients do not have to answer these questions twice.

11.10.2 Selection of the sample

Five to seven patients will be recruited in each trial site, aiming at a sample size of about 20 participants. Selection criteria will consider the following criteria: gender, age, Post-COVID-19 condition (e.g., dyspnea, fatigue, cognitive disorder, anxiety, depression), and symptom severity (based on the Post-COVID-19 Functional Scale). At least one criterion each should be represented in the study cohort. If the target number of 20 participants with all of the selection criteria will be reached before the end of the main study recruitment period, then patients calling the trial centers for an appointment will no longer be asked about participating in this sub-study.

11.10.3 Benefits and risks

Patients benefit from the opportunity to express their perceptions of health care in the context of Post-COVID-19 symptoms. Risks concern the time spent for the duration of the interview. Risks regarding data protection will be kept to a minimum by keeping to data protection standards and pseudonymization.

11.10.4 Analysis

The interviews will be analyzed according to qualitative content analysis (Schreier) using the software MaxQDA®.

11.11 Continued treatment of patients after the end of the trial

The trial medication will be finished after 28 days. Continued treatment of patients beyond this period is not foreseen.

11.12 Pregnancy

Pregnant and breast-feeding women are excluded from participation in the trial. If a pregnancy is reported during the period of the trial, trial medication will be discontinued and an early unblinding-must occur.

Every female patient of childbearing potential* must have a negative pregnancy test at baseline. Patients of reproductive potential (men and women) must agree to use highly effective methods of contraception** during the course of the study and for 1 week after the last dose of study drug(s), even if oral contraceptives are also used.

A second pregnancy test will be performed after the end of corticosteroid's biological effects, calculated for day 4 after the end of the intake of the trial medication. This is based on the rationale that according to the SmPC (Fachinformation) Predni H Tablinen® Zentiva, the plasma elimination half-life is approx. 3 h. The duration of action of prednisolone is longer than the residence time in the serum; according to the SmPC, it is 18 to 36 h in the medium dose range (the dosage of 5mg Prednisolone administered during the 3 weeks preceding the last intake at day 28 is considered to be a low dose). It follows that the drug can be considered washed out after 5 t1⁄2, i.e., after 17.5 h at the latest, it then no longer induces pharmacological effects. Since prednisolone influences the transcription and thus the expression of different genes by interaction with intracellular receptors, the effect lasts until the expression of the genes has normalized, which is described as a maximum of 36 hrs. Accordingly, we estimate the maximum plausible time for residual effects of prednisolone after the end of therapy to be 17.5 h + 36 h = 53.5 h after the last dose. Thus, there should be at least 3 days after the last prednisolone intake (including a further safety margin of 18.5 hrs) before the 2nd pregnancy test is performed.

At visit 2 (day 28), the patients are given a pregnancy test (with a sensitivity of at least 25mIU/ml) and are instructed to perform it themselves 4 days after the last medication (day 32). Patients are instructed to contact the study center to report the test result after the test. If the patient does not call on her own, the study center will contact her the day after to ask for the test result, no later than 3 days after testing.

* Following CTFG *"Recommendations related to contraception and pregnancy testing in clinical trials"*, a woman is considered of childbearing potential, i.e. fertile, following menarche and until becoming post-menopausal unless permanently sterile. Permanent sterilisation methods include hysterectomy, bilateral salpingectomy and bilateral oophorectomy. A postmenopausal state is defined as no menses for 12 months without an alternative medical cause. A high follicle stimulating hormone (FSH) level in the postmenopausal range may be used to confirm a post-menopausal state in women not using hormonal contraception or hormonal replacement therapy. However in the absence of 12 months of amenorrhea, a single FSH measurement is insufficient.

For the purpose of this document, a man is considered fertile after puberty unless permanently sterile by bilateral orchidectomy.

** Effective methods of birth control include:

- Hormonal contraception (oral, injectable, implantable, transdermal) plus a barrier method;
- intrauterine device (IUD) or intrauterine hormone-releasing system (IUS) plus a barrier method;
- bilateral tubal occlusion (females)
- Sexual abstinence
- vasectomized partner (males)

Prednisolone during pregnancy and lactation (information for healthcare professionals):

> *Although early animal studies suggested that corticosteroid exposure in the womb may cause cleft lip and palate, the majority of human studies do not support this finding. While this is reassuring, no studies have accurately investigated outcomes in pregnant women only using potent corticosteroids and further research is therefore required. Some studies have shown that women taking a systemic corticosteroid in pregnancy may have a higher chance of miscarriage or preterm delivery. However, this is thought to be at least partly explained by the fact that some of the health conditions that systemic corticosteroids are used to treat have themselves been linked to a higher chance of these pregnancy outcomes. Overall, there is no convincing evidence that corticosteroid use in pregnancy directly causes miscarriage or preterm delivery. (www.medicinesinpregnancy.org)*

Vitamin B compound during pregnancy and lactation (information for healthcare professionals):

> *No studies have investigated the chance of birth defects in babies born to women taking vitamin B12 at any dose in pregnancy. However, there are 17 case reports of babies without birth defects born to women taking high dose vitamin B12 to treat methylmalonic acidemia in pregnancy. No studies have investigated the likelihood of miscarriage or stillbirth in women taking vitamin B12 at any dose in pregnancy. No studies have assessed the chance of preterm birth or low infant birth weight following use of vitamin B12 at any dose in pregnancy. (www.medicinesinpregnancy.org)*

11.12.1 Documentation of pregnancies

The investigator must inform the sponsor immediately after becoming aware of a pregnancy by transmitting the Clinical Trial Pregnancy Form by fax +49 (0)931-201-60-39340 and document the progress accordingly. The investigator‘s documentation duty commences immediately after he/she becomes aware of a pregnancy reported in a trial patient after the first dose of the trial product or within the follow-up time stipulated in the protocol and it ends with the first examination of the child after birth.

The person responsible for monitoring (assigned by the CTCW) will supervise compliance with documentation. Any queries from the Sponsor to the responsible investigator can go through this person.

11.12.2 Documentation and follow-up of pregnancy complications and miscarriage

The investigator must document the occurrence of serious complications (as defined in ICH Topic E 6, Note for Guidance on Good Clinical Practice (GCP) (CHMP/ICH)135/95), 1997) during pregnancy while an investigational product is being taken as well as hereditary (congenital) abnormalities or birth defects as SAEs and report them to the Sponsor in compliance with the protocol.

If an AE with a suspected causal relationship with the trial product occurs, the Sponsor is responsible for compliance with the legal reporting obligations.

SAEs include:

- premature pregnancy termination – induced or spontaneous – (N.B.: a pregnancy termination for social reasons does not have to be reported),
- complications during pregnancy,
- an unhealthy or dead child and/or
- abnormalities in the child within the first 12 months after birth.

If the assessment shows that the case is a suspected unexpected serious adverse reaction (SUSAR), it must be reported in accordance with the legal stipulations.

If relevant, positive and negative experience during the pregnancy and during breast-feeding should also be reported in the development safety update report (see ENTR/CT3, 5.2.1).

11.12.3 Documentation storage

The originals of reports on the occurrence and outcome of a pregnancy exposed to a trial substance will be stored in the investigator site file and copies in the trial master file.

11.13 Patient withdrawals (Drop-outs)

Patients can leave the clinical trial early at any time at their own request without giving reasons and without consequences for their future treatment. In addition, they can be removed from the trial by the investigator. However, the duration of the drug trial is relatively short with drug intake for 28 days so few drop-outs are expected.

The reason for the removal of the person in question from the trial will be documented on the eCRF. All patients who leave the trial early will be urged to notify the investigator of this.

11.13.1 Discontinuation of (single) subjects

Discontinuation of the trail for (single) subjects can be necessary in case of:

- Patient’s request
- Patient’s withdrawal of consent
- Loss to follow-up
- Loss of contact
- Relocation of the patient

- Study termination
- Significant non-compliance
- Retroactive failure to fulfil inclusion/exclusion criteria if individual benefit/risk assessment does not overrule
- New medical conditions not allowing continuation of the protocol conform medication
- Occurrence of adverse events (e.g., allergic reaction to study medication, severe laboratory abnormalities) leading to substantial changes in the individual risk-benefit considerations that suggest a discontinuation of the study drug
- Pregnancy
- Enrolment in any other clinical trial involving an investigational product or enrolment in any other type of medical research judged not to be scientifically or medically compatible with this study
- If the patient, for any reason, requires treatment with another therapeutic agent that has been demonstrated to be effective for treatment of the study indication
- If the patient, for any reason, requires treatment with a concomitant medication that during this trial should be avoided for reasons of safety and/or efficacy

11.14 Early termination of the trial

The Sponsor is entitled to terminate the trial early for relevant medical/ administrative reasons. The reasons for stopping the trial will be documented in detail. The PI in consultation with the Sponsor or the Data Safety Monitoring Board (DSMB) can also recommend to discontinue the trial. Patients who are still being treated at the time of discontinuation of the trial must undergo a final examination, which will be documented on the eCRF. If an investigator has ethical concerns about continuing the trial, this must be reported without delay to the PI.

The Sponsor is entitled to terminate the clinical trial early if

- the patient recruitment rate is inadequate,
- serious and insoluble problems emerge concerning the quality of the data collected,
- unforeseeable circumstances occur at all of the trial sites preventing continuation of the clinical trial,
- new scientific findings during the trial prevent it from being continued,
- the DSMB recommends not to continue the trial due to the number of serious unexpected events and complications.

12 Adverse events, adverse reactions

12.1 Definitions

An **adverse event (AE)** is any untoward occurrence experienced by a person (trial patient) to whom a trial product was administered, and which is not necessarily causally connected with this treatment. It could be diseases, signs of illness, clinically significant laboratory values or symptoms which emerge or worsen after enrollment of the patient in the trial ('worsening symptoms' related to post-COVID are not classified as AEs; see 'study-specific safety-relevant events' for clarification). AEs will be recorded from day 0 till phone call 3 (day 180).

An **adverse reaction (AR)** is any untoward and unintended response to a trial product, regardless of its dosage. A response will be classified as this if a connection between the event and the trial product is considered at least possible. ARs will be recorded from day 0 till phone call 3.

An **unexpected adverse drug reaction (UADR)** is an adverse reaction whose nature or severity does not tally with the available information on the trial product as described in IB Prednisolon STADA®, Predni H Tablinen® Zentiva or IB Vitamin B komplex Hevert, see attachment). UADRs will be recorded from day 0 till phone call 3.

A **serious adverse event (SAE)** or **serious adverse reaction (SAR)** is any undesirable event or any adverse reaction which

- results in death or
- is life-threatening or
- results in persistent or significant disability or incapacity or
- requires hospitalization or prolongation of existing hospitalization or
- results in congenital anomalies or birth defects or
- is medically significant for other reasons (event for which medical intervention is necessary to prevent a serious outcome).

SAE or SAR will be recorded from day 0 till phone call 3 (day 180). The Sponsor or a second evaluator entitled by the Sponsor will check all reported SAE to see if they were expected or if there is a possible causal connection to the study medication.

A **suspected unexpected serious adverse reaction (SUSAR)** is as defined in Directive 2001/20/EC. In the current study, the compound is being used for the first time in the indication post-COVID. Information on side effects in this indication is not yet available. Accordingly, any SAR in this study will be treated as a SUSAR and reported according to Section 13 (2) and (3) of the GCP regulation. SUSARs will be recorded from day 0 till phone call 3. For reporting SUSARs the study team need to give any information needed for the SUSAR (CIOMS) form to the sponsor by a pre-filled form. In case a SAR does not lead to an unblinding, the unblinding for the SUSAR reporting is performed by the sponsor to maintain the blinding of the study team.

Study-specific safety-relevant events, day 0 till phone call 3:

- reinfection with SARS-CoV-2

If an infection with SARS-CoV-2 occurs during day 0 to 5, patients will be advised to stop the intake of the study medication. If any other study-specific events occur, in addition to being documented as an AE/SAE as usual, they will be reported promptly to the PI who will decide on clinical management.

12.2 Documentation of AEs

All AEs which occur from day 0 up to phone call 3 must be documented. If it is an AE requiring a consultation with the investigator it will be documented by the investigator on the eCRF and in the patient records. Data on symptoms and disorders which do not require consultation will be collected by the telephone interview, documented on the eCRF by the study nurse and disclosed to the investigator. If an AE **with a causal relationship to the trial medication occurs** the patient must be monitored by the investigator and/or in telephone interviews until the symptoms have subsided, the condition stabilizes (in the case of persistent impairment) or a plausible explanation for the AE has been found. All others should be monitored until the symptoms have subsided, the condition stabilizes (in the case of persistent impairment) or at least up to the last follow up (which come first).

The following details concerning the AE will be documented:

- Nature of the adverse event: symptoms, illness, accident
- Differentiation between serious/non-serious
- Evaluation of the (exacerbation of existing) concomitant conditions as an AE
- Date of occurrence/subsidence
- Assessment of the causal relationship to the trial medication
- Severity
- Action in relation to the trial product (continuation/ discontinuation), action to restore or improve the well-being of the patient
- Outcome of the event if a causal relationship to the trial or the trial product is probable.

12.3 Documentation of SAEs

The following points must be observed when documenting SAEs:

- The SAE must be documented on the relevant AE page of the eCRF and on the SAE form.
- Every SAE must be reported as fully as possible. The person responsible for monitoring must review the data for completeness at the trial site and ensure that the information on the SAE report tallies with the information in the database and with other data sources.

Cases of overdose, usage errors, etc., should be documented even if an AE does not occur.

If an investigator learns of an (S)AE that can be reasonably related to study drug he should document and report this event even after the patient's study end.

12.4 Reporting of AEs and ARs

12.4.1 Notification duties of the Investigator (§ 13 (1) – (6) GCP-V pursuant to § 42 AMG)

SAEs

The investigator must inform the CTCW about the occurrence of a SAE **without undue delay but not later than within 24 h** of obtaining knowledge of it. The investigator should be done by using the SAE form in the eCRF (in case of technical problems manually by fax of the report to +49 (0) 931-201-60-39340). The investigator must then transmit a detailed written report on the SAE in question to the Sponsor or its representative.

Death of a trial patient

In the event of the death of a patient, the investigator must also provide the competent ethics committee, the competent federal higher authority (BfArM) and the Sponsor (or its authorized representative) with all the additional information necessary for them to fulfill their tasks (on request).

Reporting of (S)AEs after the trial has ended

If an investigator learns of an (S)AE that can be reasonably related to study drug he should notify the sponsor even after the patient's study end.

12.4.2 Notification duties of the Sponsor (§ 13 (1) – (6) GCP-V pursuant to § 42 AMG)

AEs

The Sponsor or its representative must document all the AEs of which it is notified in detail and transmit these on request to the competent federal higher authority (BfArM).

SUSARs

The Sponsor or its representative must notify the competent ethics committee, the competent federal higher authority, Federal Institute for Drugs and Medical Devices (BfArM) as well as the investigators participating in the clinical trial and the DSMB of each SUSAR of which it is notified promptly but at the latest **within 15 days** after it becomes aware of it. The SUSAR assessment will be carried out by the Principal Investigator (PI).

In the event of a SUSAR which results in **death** or is **life-threatening**, the Sponsor (or its representative) must provide the competent ethics committee, the competent federal higher authority (BfArM) as well as the investigators participating in the clinical trial with all the information of importance for the assessment promptly but at the latest **within seven days** after it becomes aware of it and provide other relevant information within a maximum of **a further eight days.**

Further review of the benefit/risk assessment

The Sponsor or its representative must inform the competent ethics committee as well as the competent federal higher authority (BfArM) of any circumstances that require a further review of the benefit/risk assessment of the trial product promptly but at the latest **within 15 days** after it becomes aware of them. This includes in particular:

- Individual reports of expected SARs with an unexpected outcome,
- An increase in the frequency of expected SARs assessed as clinically relevant,
- SUSARs which occurred after the patient had already finished the clinical trial,
- Events that are connected with the performance of the trial or the development of the trial product and may possibly interfere with the safety of the patients.

List of all SARs and Development Safety Update Report

The Sponsor or its representative, in consultation with the PI, must provide the competent ethics committee as well as the competent federal higher authority (BfArM) with a list of all the SUSARs which have occurred during the clinical trial as well as a report on the safety of the patients once a year or on request.

Measures to protect against immediate danger

If the safety of the patients is affected and the PI as well as the investigator take measures to protect them from immediate danger, the PI will in the event of immediate danger inform the competent ethics committee and the competent federal higher authority (BfArM) about these measures as well as the triggering circumstances.

Data Safety Monitoring Board (DSMB)

A 4-member team of independent experts has been established as a Data Safety Monitoring Board (DSMB) in line with EMEA/CHMP/EWP/5872/03 Corr. to assess the safety aspects of the clinical trial regularly. The head of the independent body is Jörg-Janne Vehreschild. For details of the members and their profession please see the “Participating persons/institutions” overview (p. 10).The DSMB receives confidential information on the progress of the study regularly and is responsible for the following tasks:

- Approval of the trial protocol with regard to patient safety,
- Assessment and discussion of safety aspects (SAEs, SUSARs and trial-related hospital referrals),
- Issue of a recommendation to the sponsor about how to proceed (to continue or discontinue the clinical trial) according to the frequency and severity of safety-relevant events.

Details of the tasks and responsibilities of the DSMB are specified in the DSMB charta.

13 Documentation

It is the investigator's responsibility to make sure that the trial is conducted in accordance with ICH-GCP guidelines, the AMG, as well as the protocol and ensure that the data are correctly entered into the eCRF. All the data recorded in this trial must be entered into the eCRF by duly authorized persons. This also applies to data on people who were removed from the trial. If patients withdraw their consent, the pseudonymization list will be censored, patients will be informed about data protection according to the DSGVO).

The investigator must note participation on a special patient identification list. This makes it possible for the patients to be identified at the clinical trial site and contains patient number, full name, date of birth and date of admission to the clinical trial. The patient identification list remains at the trial site after completion of the trial. In addition, in this clinical trial the participation of the relevant people must also be noted in the patient records (trial product, patient number/ randomization number, start and end of trial).

Furthermore, it must be ensured that the people who are responsible for eCRF documentation can be identified. A list containing the signature and initials of the people permitted to make entries into the eCRF will be kept in the investigator site file and the trial master file.

13.1 Electronic case report form (eCRF)

Trial with electronic documentation

It is the investigator's responsibility for data collected as part of the clinical trial to be entered correctly and completely in the database created specifically for this trial. It is possible to use worksheet as a documentary aid.

The PI is responsible for the correct and complete entry of data collected at their trial center. Data entry at the trial sites must be carried out only by authorized individuals.

Corrections to the eCRF must be made only by authorized individuals or by the responsible investigator and must be justified. Corrections are made in a way that the old entry can still be retrieved. All data and corrections will automatically be logged with the date, time and person making the entry.

13.2 Investigator site file

The Sponsor will provide the trial site with an investigator site file. This holds the documents required for the clinical trial. The up-to-datedness and completeness of the investigator site file will be checked as part of the monitoring process on a regular basis. It must be kept for a minimum of 15 years after the completion or discontinuation of the trial.

13.3 Data archiving

13.3.1 Archiving duties of the Sponsor

All of the essential clinical trial documents must be kept by the Sponsor for a duration of at least 15 years after the end or discontinuation of the clinical trial. The Sponsor must archive the trial-related paperwork and documents in accordance with the legal provisions.

13.3.2 Archiving duties of the investigator

Records and documents connected with the testing or dispensing of trial products (e.g., case report forms, consent declarations, medication distribution lists and other relevant documents) must be kept by the investigators for a minimum of 15 years.

14 Quality Control and Quality Assurance

Clinical monitoring, audits and (if requested by authorities) inspections will be conducted during the clinical trial for quality assurance purposes.

In accordance with the principles of ICH-GCP, the Sponsor takes responsibility for monitoring of the study through use of a contract research organization (CRO). The CTCW will serve as the CRO. The objectives of the monitoring procedures are to ensure that the trial subject's safety and rights as a study patient are respected, that accurate, valid and complete data are collected, and that the trial is conducted in accordance with the trial protocol, the principles of GCP and local legislation.

All aspects of the protocol are to be complied with during the trial. Should amendments become necessary, these are to be discussed immediately and in detail with the coordinating investigator resp. the Sponsor. The agreement reached is presented in writing in the form of a protocol amendment. The primary investigator either applies for the approval of the responsible ethics committee ("substantial" amendment) and authorities or informs the responsible ethics committee and authorities accordingly ("non-substantial amendment").

Every deviation from the trial protocol is to be entered into the case report form including reasons given by the investigator or a person designated by him/her.

All persons involved in the trial are to be informed of their responsibilities. The correct execution of all tasks related to the trial is to be supervised by the investigator during the course of the trial.

14.1 Monitoring

The investigators will also agree to the person responsible for monitoring carrying out a review of the data to make sure that the data is being recorded satisfactorily and to ensure compliance with the protocol.

Furthermore, they will also agree to cooperate with this person and to provide him/her with all required information, whenever necessary. This includes access to all documents connected with the trial, including the originals of the trial-related patient records. The responsibilities of the investigators include maintaining patient records as fully as possible, recording information on the medical history, concomitant conditions, admission to the trial, visit dates, results of tests/examinations, medication dispensing as well as AEs. The monitor will also be enabled to review the data and to compare them with the relevant patient records in accordance with ICH-GCP guidelines at pre-defined time intervals in order to ensure compliance with the protocol and continual data recording. During this, all the original medical reports which are necessary as a source for the information on the eCRF and in the database may be reviewed. The participants will have agreed to such a review by signing the informed consent form.

In the clinical trial, monitoring will be carried out by a monitor from Center for Clinical Trials of the University Hospital Würzburg. An adaptive monitoring plan will be implemented following a specific risk assessment. The scope and contents will be laid down in a study-specific work instruction.

14.2 Auditing

Within the framework of trial sites audit, the investigators agree to allow an auditor delegated by the sponsor, the supervisory authorities or the registration authorities, or a member of the ethics committee responsible, to verify that the execution of the clinical trial is carried out in accordance with the trial protocol and GCP and to check all the relevant documents whereby random sample checks of the original documents (source data) can be undertaken. The clinical investigator shall allow this person, as well as the monitor, access to all premises used for trial execution and documentation of the clinical trial.

Both monitoring and auditing may require the presence of the clinical investigator for several hours.

15 Data entry and data management

Trial with electronic documentation

The CTCW is acting as a trial support center and data management center. A computerized data entry and management system will be developed by the CTCW where all study data relating to the described main study can be entered.

A closed and password protected data entry system has been designed to ensure that only the responsible data entry person and the supervisor can enter and/or edit data and this can be done only by using the programs and/or utilities available on the menu system. An audit trail will be created by data and user stamping. Range checks, review screens, and various error trapping routines are built into the system as quality control procedures. The data will be managed and processed by the Center for Clinical Trials of the University Hospital Würzburg.

15.1 Data Collection and Data Correction

15.1.1 Electronic Data Capture System

The PIs or other delegated participating site staff will use electronic case report forms (eCRF) to capture all relevant data pertaining to patients enrolled in this study in a timely manner.

While entering data, edit checks will be performed to verify the data electronically. If certain data are not available or not applicable this will be indicated as such in the appropriate space in the eCRF. It is the responsibility of the PIs to assure that all data gathered in the course of the study are entered completely and correctly in the respective database. A PI or Co-Investigator must confirm the integrity of the data by electronic signature. Completeness and plausibility checks can be performed from remote desktop. Proper completion of the eCRFs will be checked by the study monitor. The monitor supervises the progress of the study and performs the source data verification on-site. The monitor will initiate any necessary additions and corrections immediately after checking with the PIs.

15.2 Source Documentation

All data entered into the eCRF will also be documented in the source documents. Participating sites need to ensure that source data are available either in paper form or that the monitors have access to the electronic data of the study patients.

15.3 Database and Data Management

Implementation and programming of the study database will be performed by data management of the CTCW. The system provides the capability to perform the major data management activities within a consistent, auditable and integrated electronic environment (data security, data entry, data validation). All data will be entered directly in the eCRFs and saved in the study database. Range, validity and consistency checks will be implemented in the system for application during data entry.

Data entries will be recorded routinely via audit trail, as well as data modifications and data corrections. In case of necessary corrections or existing data inconsistencies, data queries will be generated consecutively by the data manager. Queries will be sent electronically to the study team of the respective trial site. Afterwards, data in the database will be corrected according to the answered and resolved queries. After database lock data are imported into standard statistical software systems and analyzed by biostatisticians at the CTCW.

15.4 Confidentiality of Study Data and Data Protection

The patient identification list will be under strict control of the PIs and will not be transferred to the data management center CTCW. Data recorded in the eCRF will be pseudonymised. Any personal data (such as medical history) will be identifiable only by a unique, private patient identification code (PreVitaCOV patient ID).

In accordance with applicable legal provisions, individuals who have direct access to source data shall take all the necessary precautions to ensure the confidentiality of information relating to IMP, research, patients and particularly their identity, as well as the results obtained. These individuals, like the investigators, are subject to the conditions of professional secrecy.

The CTCW will ensure no unauthorized access to the computer system takes place and that no data loss occurs according to adequate data backup procedures. Only specified persons involved in the study will be given authorization to enter or access data in the study databases based on predefined roles. There will be a complete audit trail of all transactions.

All employees of the CTCW involved in the study are required to actively protect confidential data against access by third parties in accordance with the prevailing guidelines.

All patient-related data will be recorded after pseudonymisation. The PreVitaCOV patient ID is defined according to the following standardized procedure:

All patients are numbered by the participating site code consisting of one numerical digit for the specific participating site number and four numerical digits for consecutive numbering of study patients per participating site. The patient number in the order of study enrolment is starting from ‘0001’ in every participating site. All subsequent patients are assigned consecutive numbers in chronologic sequence (e.g., second patient is assigned patient number 0002 and the third patient is assigned patient number 0003, etc.). Consequently, each patient included into the study will be uniquely identified in the study by a combination of his/her participating site and patient number which is their PreVitaCOV patient ID.

All patients eligible for the study will be documented in the screening log. In case a patient will not be included, this will be documented with the specific reason for non-inclusion.

The numbering will be performed by the eCRF. Additionally, the patient will be randomized by the CTCW via the eCRF with the deposited randomization list (by problems with the availability of the eCRF by the printed randomization list through the study team). The PIs will be maintaining a patient identification list linking the identification number with the personal data of the patient. This list will be kept strictly confidential at the participating site under control of the PIs. After termination of the study, this list has to be archived at the participating site following local guidelines.

15.5 Archiving

At the end of the study and after all close out visits have been completed, archiving of study documents and study data will be performed according to the internal SOP of the data management center at the CTCW. Archiving of the clinical database including the audit trail will be performed by the data management center in a machine independent format and will be sent to the Sponsor for archiving. The originals of all essential study documents are filed in the Trial Master File (TMF), which will be archived at the University Hospital Würzburg for at least 15 years after preparation of the final report. PIs will file all participating site-specific documents (e.g., correspondence with ethic committee), the patient identification list, the signed informed consent forms and general study documents (e.g., Protocol, amendments) in the Investigator Site File (ISF), which will be archived at the participating site for at least 15 years or as long as is required by the local regulatory requirements if longer.

The data will be reviewed through programmed range, validity and consistency checks. There may be queries raised by the trial software and by duly authorized individuals. The investigator can check and address the discrepancies which have occurred from the queries.

Once the trial has ended, the database will be locked after all entries have been made and queries clarified. This process will be documented.

16 Statistics

Analyses will focus on the following Hypotheses:

Pilot study: The retention rate on day 28 will be about 85% of all included patients.

Confirmatory study: There are differences among the 4 treatments regarding the improvement of PC19 symptoms on day 28 as assessed by a tailored score based on the PROMIS. At this the improvement is lowest in the placebo group as detected by post-hoc tests.

The trial result is positive if superiority of any treatment is demonstrated over placebo. To further differentiate possible treatment effects, all secondary outcomes will be analyzed with regard to differences in treatment effects and post-hoc tests for superiority of any treatment arm compared to placebo.

Furthermore, the laboratory parameters and the safety outcomes will be compared between the groups.

16.1 Sample size planning

Pilot study

The aim of the sample size calculation for the pilot study was to ensure that we could estimate the retention rate, i.e., the proportion of patients who completed the 4-week intervention, with sufficient precision. Sufficient precision here means that the width of the corresponding 95% confidence interval doesn't exceed 0.15 (15%). Conservatively we base the calculation on the exact (Clopper-Paerson) method. Furthermore, because the target retention rate in this trial is 0.85 (85%), we assume for the calculation an observed sample proportion of 0.85. Then, 100 patients will be needed to yield a confidence interval with the postulated precision.

According to the study design, 4 equal sized treatment groups are planned. Therefore 4*25 = 100 patients are to be randomized in a 1:1:1:1 ratio at the beginning.

Confirmatory study:

<u>Summary of sample size calculation</u>:

The aim is the detection of an underlying main effect corresponding to at least 3-points (T-score) on the outcome scale. The outcome scale is the scale for the changes in our primary endpoint from baseline to day 28. The primary outcome measure is a tailored weighted mean T-score from 5 PROMIS subscales.

The range of values for the primary outcome measure is 16.7 – 57.7

In the case that this 3-point difference is the smaller one of the two underlying main treatment effects (vitamin B compound and prednisolone) the sample size will ensure that both effects will be detected with sufficient power (80%). In the case that this 3-point difference is the stronger one of the two underlying main treatment effects the sample size will ensure with sufficient power that at least this stronger main effect will be detected.

Within the primary analysis four treatment groups are compared: patients with neither intake of vitamin B compound nor prednisolone, patients with intake of vitamin B compound only, patients with intake of prednisolone only and patients with intake of both vitamin B compound and prednisolone combined.

We base the sample size calculation of the primary analysis for the primary endpoint by a two-factor analysis of variance (ANOVA). The dependent variable within the ANOVA model is our primary endpoint, operationalized as the change in the tailored PROMIS score from baseline to day 28 (visit 2). Furthermore, the two-factor ANOVA model contains the two main factors vitamin B compound and prednisolone, both with two factor levels: intake no or yes. Finally, the model contains the interaction factor with four levels. Within this model we will estimate the main- and interaction effects and test the two null hypotheses of no difference between the two-factor level means in each case (for the main factors vitamin B compound and prednisolone) and test the null hypothesis of no interaction between the factors vitamin B compound and prednisolone by the corresponding F-tests with one nominator degree of freedom in each case.

<u>Assumptions for sample size calculation</u>: K. Kroenke[50] et al. reported a difference in T-score of 3 to 4 points as a **reasonable minimally important difference** for the PROMIS depression scales. Considering this for our primary outcome measure, as a weighted mean of T-scores from 5 PROMIS short form instruments, we aim to detect such a difference of 3 points as a significant effect. The same seems to be reasonable for change scores that means for our primary endpoint. A 3-point difference between the effects of the two level (*no intake* versus *intake yes*) for a main factor within the linear model for a two-factor experiment corresponds to a standard deviation of means (SM) of 1.5.

Conservatively, we claim that the sample size has to be adequate in this sense that **any** underlying main effect corresponding at least to a standard deviation of means (SM) of 1.25 can be detected as a significant deviation from the null hypothesis of no effect with a power of 80% by the corresponding F-test at a significance level $\alpha = 0.05$. Furthermore, we assume a standard deviation (SD) of 7.5 points for the primary endpoint variable (see section 16.4.1 Outcome measures). With this expressed in terms of **effect sizes**, the assumptions above correspond to an effect size about 0.167 (precisely 1.25/ 7.5).

A sample size of 4*72 = 288 ensures a power of 80% to detect any main effect with an effect size of at least 0.167 as significant deviation from the null hypothesis of no effect.

Assuming a lost to follow up rate of 15%, **4*85 = 340** patients are to be included in the study.

<u>Remark:</u> The difference between the two underlying marginal means for one factor (vitamin B compound or prednisolone) depends additionally on the strength of interaction effects!

16.2 Placebo as a comparator

Using placebo as a comparator will not only overcome context effects of the trial but also balance for placebo effects. Yet, the use of placebo as a comparator will result in some participants not receiving the active treatment. However, as long as effective evidence-based treatment options are lacking, participants randomized in the placebo will not be deprived of the standard care.

PC19S is a new entity and therefore only few placebo-controlled trials are available yet. Based on the current literature and evidence from similar conditions, the placebo response rate in patients

suffering from PC19S is expected to be between 20% and 40%. Thus, a recent study involving patients with PC19S and olfactory impairment reported that 42% of participants in the placebo group improved.[34] Other studies of patients with long-term conditions like Lyme disease found a placebo response rate of 36%.[60] A systematic review and meta-analysis on placebo responses in chronic fatigue syndrome which shares some similarities with PC19S reports a placebo response rate of 19.6%.[61]

16.3 Randomization

Computerized central randomization and generation of the randomization list recording the code numbers and assignment to the treatments will take place at the CTCW; the process will be overseen by the trial statistician.

Patients will be allocated to the four treatment groups via stratified block randomization with varying block size. The single stratification factor is the study center.

Based on the randomization list, the trial medication will be labeled with code numbers by the Hospital Pharmacy of the University Hospital Hamburg. On entry to the study, the patients will receive the code number marked on their medication.

16.4 Statistical methods

16.4.1 Outcome measures

Primary outcome (primary endpoint of trial)

Pilot study

The primary outcome for the pilot study is the retention rate at day 28 of the trial.

Confirmatory study

The intention for the primary endpoint of the confirmatory study was to construct a tailored measure for the disease burden of patients with Post-COVID-19-Syndrome. This should be a patient reported outcome not too burdensome for patients. We operationalized this as a mean of the T-scores from 5 PROMIS® Short Form instruments:

- PROMIS Short Form v1.0 – Fatigue 4a
- PROMIS Short Form v1.0 – Dyspnoea Severity 10a
- PROMIS Short Form v2.0 – Cognitive Function 4a
- PROMIS Short Form v1.0 – Emotional Distress-Anxiety 4a
- PROMIS Short Form v1.0 – Emotional Distress-Depression 4a

At this, we have to distinguish between negatively-worded concepts (like Fatigue, Dyspnoea, Anxiety and Depression) and positively worded concepts like Cognitive Function. Therefore, the outcome measure isn’t a simple mean but a linear combination adding up the 4 T-scores for the 4 PROMIS Short Forms Fatigue, Dyspnoea Severity, Anxiety, Depression and subtracting the T-score for Cognitive Function.

Mean and SD for all T-scores of the five validated instruments are 50 and 10 within the reference population from the population 2000 General US census. Therefore, by construction above the mean of our primary outcome measure in the reference population will be 30. But we don't know the variance and with this don't know the SD in the reference population. For this, we would need to know all 10 pairwise covariances resp. all 10 pairwise correlation coefficients in the reference population between the 5 T-scores. But we can get a very rough approximation: if we assume for all 10 pairs of the 5 T-scores a medium strength of correlation, quantified by a Pearson correlation coefficient between 0.4 and 0.5, the SD of our transformed Post-COVID-19 tailored PROMIS total score will be between 7 and 8 (rather conservative).

Our primary outcome measure in the confirmatory study is defined as a change score, the difference in our specifically tailored outcome measure from baseline (visit 1) to visit 2 after 28 days:

Tailored-score (V2) – Tailored-score (V1).

Assuming a medium strength for the correlation between baseline measurements and measurements 28 days after baseline, we assume for this change score roughly a SD between 7 and 8 points too, justified by experience gained from earlier change score analyses.

16.4.2 Definition of analysis populations

The primary and secondary outcome measures (superiority) and the AEs will be analyzed in the intention to treat (ITT) and per protocol (PP) population. As the purpose of this trial is to compare treatment strategies, all randomized trial patients in the relevant group will be analyzed, regardless of whether or not they have taken the trial medication. The methods used to deal with missing data are described in section 16.4.3 below. The PP population consists of all patients in the ITT population with protocol-compliant ingestion of the trial medication, full documentation of the symptom scores within the first 28 days and no serious protocol violations, in accordance with the blinded assessment of the steering committee.

16.4.3 Data analysis

Before analyses the entered data will be checked for plausibility and completeness.

<u>Data analysis for the primary endpoint of **the pilot study**: retention rate at day 28:</u>

For the first endpoint of the pilot study, the proportion of patients who completed the 4-week intervention, point and interval estimations (95% CI, confidence interval) will be given. This will be done combining all 4 treatment groups.

<u>Data analysis for the primary endpoint of **the confirmatory study**: the specifically tailored PROMIS total score, derived from 5 Short Form instruments:</u>

- Summarizing descriptive statistics at baseline and day 28 (visit 2) for the tailored score within the 4 treatment groups
- Mean change in the tailored score from baseline to day 28 (visit) with 95% CI within the 4 treatment groups
- Factorial ANOVA for the dependent variable change in the tailored score from baseline to day 28 (visit 2)

- Problems investigated by corresponding F-tests within a model of a 2-factor ANOVA (factorial design)
    - Are there differences in the mean change score among patients treated with and without vitamin B compound?

    Null hypothesis: there is no effect for administration of vitamin B compound

    - Are there differences in the mean change score among patients treated with and without prednisolone?

    Null hypothesis: there is no effect for administration of prednisolone

    - Are there any vitamin-B-compound-prednisolone combinations that exhibit a mean change score that cannot be explained by administration of vitamin B compound and/or administration of prednisolone alone?

    Null hypothesis: there is no such interaction effect.

Further analysis within the 2-factor ANOVA:

- Estimation of marginal means for the primary outcome measure for the two factors intake of vitamin B compound (no/yes) and intake of prednisolone (no/yes) with 95%CI
- 95% CI for the mean change score within all 4 factor combinations of vitamin B compound and prednisolone.

For all secondary endpoints listed in section 2.1 the corresponding total sum scores and if applicable subscores will be calculated for the different visits together with the corresponding change scores from baseline to day 28. Where appropriate, convenient transformations will be made.

Generally, all study variables will be presented using the appropriate descriptive statistics according to the measurement scale of data and for interval and ratio scaled data, respectively, according to the properties of the observed distributions (e.g., outlier values, skewness). Measures of frequency, of central tendency and dispersion or variation will be given as appropriate.

That means, for continuous variables the number of missing and of non-missing observations, arithmetic mean and/or median, range, SD or interquartile range, minimum, maximum. Frequency tables will be used to summarize categorical variables for the 4 treatment groups at different points in time. If appropriate, shift (change from baseline) tables will be given. For binary variables, numbers of missing and non-missing observations will be given together with the corresponding proportions. Shift tables (change from baseline) will be given if possible and appropriate.

In exploratory analyses, factors (such as trial center, age, symptom duration or laboratory parameters) potentially influencing the primary outcome will be investigated using appropriate multivariable modelling.

Analysis for the secondary endpoint use of on-demand medication (patient diary): to compare the counts of on-demand medications within the period from baseline to day 28 between the four treatments negative binomial regression (NBR) models resp. zero-inflated negative binomial regression (ZINBR) models will be used. Within these models the count of using additional on-demand medication is the dependent variable, furthermore the models include the two binary main factors treatment with vitamin B compound, treatment with prednisolone as well the interaction factor. Using NBR

models we can account for possible over-dispersion and using ZINBR models we can account for a possible elevated frequency of zero accounts in addition.

All secondary outcomes will be analyzed in exploratory analyses. Further details on the statistical analyses will be provided in a detailed statistical analysis plan.

Safety outcomes will be analyzed by reporting frequencies (percentages) of (S)AEs as well as the number of patients with worsening symptoms for each group.

Planned statistical analyses will be done using the current SAS version (SAS Institute Inc., Cary, NC, USA) as well as the current IBM SPSS version.

For selected secondary endpoints (specified within the detailed statistical analysis plan) secondary analyses will be carried out for the corresponding change scores from baseline to day 28 (where appropriate after a convenient transformation) in the same manner as for the primary endpoint by a 2x2 factorial analysis of variance.

Statistical methods for the analyses of changes from baseline to day 28 in outcome variables from physical examination (auscultation of chest and heart, orientating neurological examination) depend on measurement scales (interval, ordinal, nominal, dichotomous) and data types (categorical, counts, numerical, continuous, ratios) in each case. Descriptive analysis may comprise appropriate summarizing statistics and shift tables. Confirmatory analysis resp. model-based analysis may comprise variance and covariance analysis as well as hierarchical models like hierarchical binary or ordinal logistic regression. Details for this as well as for all other statistical analyses will be given within the SAP.

Missing data:

For the calculation of the primary endpoint the free Health Measuring Scoring Service (HMSS) will be used to obtain T-scores for the 5 PROMIS Short Forms (Fatigue, Dyspnea Severity, Cognitive Function, Anxiety, Depression). To indicate when a patient did not answer an item, whether because it was skipped or not applicable, either the word “SKIP” or a blank field will be listed and with this the HMSS algorithm will deal with missing data at the calculation of T-scores.

Generally, multiple imputation at the score level will be used for the patient reported outcome measures in this study.

16.3.4 Blinding assessment and interpreting data from questionnaires in the case of imperfect blinding

Because the primary endpoint and many of the secondary endpoints in the study are subjective in the sense that based on questionnaires, possible problems associated with unblinding have to be considered. Therefore, we will try to measure the degree to which the blinding was successful in the study by appropriate statistical methods. Blinding fails when patients become aware of the treatment arm they were assigned or when clinicians administering the treatment or assessing the state of disease becomes aware of the assignment for particular patients.

Therefore a post-trial, „blinding“ questionnaire is planned to quantify the patients‘ knowledge and study personal resp. belief about the administered treatment. For this the patients both in the active

treatment groups and in the placebo group are ask to guess which treatment they received. The questionnaire admits the following five choices:

1: I believe to be in the placebo group

2: I believe to be in the prednisolone group

3: I believe to be in the vitamins group

4: I believe to be in the group with combined prednisolone and vitamins treatment

5: I don't know / I'm undecided about the treatment group (**DK**)

For the analysis we will use two blinding indexes (BIs), James‘ BI and the generalized Bang blinding index by Williamson (FBI, 2014) and calculate point- and interval estimates (95% two-sided CI) for these indexes.

James‘ BI was developed already in 1996 and is well known. But this blinding index has received criticism because the very strong influence of the DK responses and that it generates only a single value for the entire study that means no possibility for an arm-specific blinding index. The generalized Bang blinding index by Williamson (FBI, 2014) is sensitive to both indicators of successful blinding: excessive DK responses or / and random guessing among non-DK responses. The method yields very interpretable arm-specific index values and is applicable to studies with any number of treatment arms (provided the sample sizes are large enough). Especially important, the method is appropriate for hypothesis testing, e.g., regarding random guessing. Under the null hypothesis of random guessing in the auxiliary questionnaire the expected value of each arm-specific FBI_i (i=1, 2, 3, 4) is zero. The value of the FBI_i can be interpreted as the proportion guessing correctly the own assigned arm beyond random balance.

For each single treatment arm unblinding may be claimed if the corresponding two-sided 95% CI does not cover 0. For the whole study claiming unblinding can be based on a two-sided simultaneous 95% CI with a multiple comparison correction. This blinding index is a generalization of Bang's blinding index (BI, 2004) which has advantages over the James index but is applicable only in the case of two treatments arms, contrary to the PreVitaCOV trial with 4 arms.

To detect unblinding possibly due to possible failure in protocols, the amount of blinding will be additionally investigated in each of the three centers and compared with one another. That means the binding indexes will be calculated also for each center separately with corresponding 95% CI. Subsequently statistical pairwise comparisons will be done. Consider the case that the blinding assessment hints to a substantial level of unblinding that means the blinding in the study was not successful. In this case the main trial results, especially the primary outcome, change in symptom severity to day 28 as assessed by the specifically tailored total score based on PROMIS, needs to be interpreted carefully.

Generally, it can be expected that the effectiveness of the administration of vitamins or/and prednisolone depends on the inherent effects of the treatments *as well as* the patients' expectations. Therefore, within a secondary analysis the effectiveness of the treatments will be inferred by a kind of „like-for-like „comparisons. Firstly, the patients were stratified, each stratum contains the patients who share the same combination of the intervention (the 4 treatment arms) and the same expressed

belief regarding their group assignments (the 5 choices listed above). The distributions of the main outcome variable will be characterized by summarizing descriptive statistics in each stratum. Subsequently the mean primary outcome in the subgroup of patients assigned to the placebo group whose feedback expresses belief also in the placebo group assignment will be compared only with the mean primary outcome of the subgroup of patients assigned to the vitamins group who equally expressed belief in the placebo group assignment. In the same way mean outcomes of patients actually assigned to the vitamins group and mean outcomes of patients actually assigned to the placebo group will be compared within the population of patients whose feedback expresses belief in the vitamins group assignment as well as within the population of patients with the „DK“(don’t know) answer. The same procedure will be applied for the comparison of the mean outcomes of patients actually assigned to the prednisolone group and mean outcomes of patients actually assigned to the placebo group. Finally, this will be done for the comparison of the mean outcomes of patients actually assigned to the combination (vitamins and prednisolone) group and mean outcomes of patients actually assigned to the placebo group.

Clearly partially small strata sizes are to be expected. Therefore, comparisons will be done, if indeed possible, by two- tailed t test / Mann-Whitney test / permutation test depending on sample sizes and properties of the observed distributions.

17 Reporting

Minutes of the meetings of the various bodies will be produced to document the progress and development of the trial. Regular interim reports will be submitted in accordance with the guidelines of the Bundesministerium für Bildung und Forschung (BMBF).

17.1 Trial report

All the information concerning this clinical trial is to be treated as confidential. The statistical analysis and report (produced by CTCW) and the final scientific report (produced by the coordinating investigator, Prof. Gágyor) will be evaluated and signed by the PIs of the other trial sites as well as all other responsible individuals.

17.2 Publications

The results of this clinical trial will be published. Publication or a presentation of the results requires prior comment and approval by the coordinating investigator. In all publications, data protection for all the patient data must be ensured. Furthermore, the clinical trial will be registered at www.clinicaltrials.gov.

By signing the bottom of the protocol, the investigators are declaring their agreement to the results of this clinical trial being submitted to the competent federal higher authority (BfArM). At the same time, they are agreeing to their names, addresses, qualifications and scope of participation in the clinical trial being made known in this connection.

18 Ethical, legal and administrative aspects

18.1 Responsibilities of the Sponsor and investigator

Pursuant to § 4 AMG the coordinating investigator as the delegate Sponsor of the trial (investigator initiated trial, IIT) will ensure that it is performed in accordance with the existing laws and provisions, in compliance with ICH GCP guidelines, the Declaration of Helsinki as well as the provisions of the AMG and the GCP Ordinance. The PIs of all trial sites will accept the requirements of the signed protocol.

His/her responsibilities will include:

1. Understanding the properties of the trial product described in the information for healthcare professionals
2. Understanding and implementing the treatment plan
3. Ensuring that sufficient time and capacity are available to perform the trial
4. Gathering and documenting the data correctly, reporting
5. Making available all of the data to the Sponsor/ coordinating investigator, for monitoring, or to the relevant authorities for audits and/or inspections
6. Ensuring that the information on patients as well as all of the information received by the Sponsor/coordinating investigator on all of the persons involved in the trial is treated confidentially
7. Declaring the involvement of persons potentially dependent on the investigators
8. Declaring information on possible financial and other interests of the investigators in connection with the trial products.

Pursuant to § 4 AMG, the relevant investigators are responsible for the performance of the clinical trial at the trial site.

18.2 Ethics committee opinion and notification of the authorities

The application will be submitted to the ethics committee in charge as well as to the Federal Institute for Drugs and Medical Devices (BfArM) on behalf of the coordinating investigator in compliance with § 7 GCP-V pursuant to § 42 AMG by CTCW. (In addition, the competent state authorities in Würzburg, Tübingen and Kiel will be notified of the clinical trial in compliance with § 12 GCP-V pursuant to § 67 AMG by the respective PI of the appropriate site.

18.3 Patient information and informed consent

Before the trial starts, every patient participating in the trial must give the investigator written consent after being fully informed of the nature, significance and scope of the clinical trial verbally and in writing in a manner which they are able to understand. The contents of this information will be documented on the informed consent form. The patients will be notified if new findings about the trial product emerge during the trial.

The declaration of consent to participate in the clinical trial will be dated and signed by the patient as well as by the local investigator. One copy of the signed patient information/ informed consent will be given to the patient and the second copy will be placed in the investigator site file.

It is expressly pointed out that no trial-related tests/ examinations whatsoever are permitted to take place before a legally valid informed consent declaration has been obtained.

The consent to participate can be withdrawn by the patient at any time without giving reasons and without any detriment to him/her.

18.4 Patient insurance

The prescribed patient insurance has been taken out with the following insurer for all of the patients in the trial on behalf of the Sponsor pursuant to § 40 para. 1 No. 8 and para. 3 AMG:

HDI Global SE

Riethorst 2

30659 Hannover

This provides insurance for trial-related injuries to health with a maximum sum covered of 500.000 € per patient. This insurance covers any injuries which the patient suffers directly or indirectly as a result of the trial product or interventions connected with the clinical trial as well as securing travel accident insurance for all patients going to and from the site.

In order not to jeopardize the insurance cover, the trial patients must adhere precisely to the instructions of the trial staff. The investigator must be informed promptly of any emergency treatment.

An injury to health that might have occurred as a result of the clinical trial must be reported promptly by the patient to the investigator and the insurance company. Moreover, the patients must take all reasonable steps to clarify the cause and scope of the injury which has occurred.

The trial patients will be given a copy of the insurance terms by the investigator.

18.5 Data protection and secrecy

The recording, disclosure, storage and analysis of personal data within this clinical trial will take place in accordance with the legal provisions (Data Protection Laws of the Federal States, German Federal Data Protection law, DSGVO). A prerequisite for this is the voluntary agreement of the patients as part of the consent process prior to participation in the clinical trial. They will be informed of the following as part of the consent process for this clinical trial:

1. Data collected as part of this clinical trial will be recorded on paper case report forms or an electronic data storage media, will be treated in strict confidence and will be transmitted without mention of any names (pseudonymized) only to:
   - the Sponsor for scientific analysis and assessment of AEs,
   - the competent supervisory authorities (the regional councils and the German federal higher authority, BfArM), the ethics committees involved (see 18.2) and the European database to review the proper performance of the trial as well as to assess the trial results and AEs.

2. If necessary to review the clinical trial, authorized and secrecy-bound representatives of the Sponsor (monitoring, auditing) and/or of the competent supervisory authority can inspect the personal data held at the trial site. The investigator is released from the duty of secrecy for this measure.
3. The consent to the collection and processing of personal data as part of this clinical trial cannot be withdrawn. Patients will be informed that they can stop participating in the clinical trial at any time – without giving reasons and without any ensuing disadvantages. Upon withdrawal of consent, the data stored at this time will continued to be used only if necessary to assess the effects of the trial interventions. In this case, they will be used in an anonymized manner that is, without mentioning a person’s name or parts of his/her birthday, to ensure that his/her protection-worthy interests concerned are not harmed.

19 Protocol amendments

To ensure largely comparable conditions at all trial sites as well as in the interests of flawless data analysis, amendments to the agreed trial conditions laid down in the protocol are not envisaged.

However, amendments to the trial conditions are possible in exceptional cases. They will take place only by reciprocal agreement between the PIs and the Sponsor. Any amendment to the procedure set out in the protocol must state the relevant reason, and be signed by all project partners of the trial. The amendments will then be considered part of the protocol. If necessary (e.g. in the event of a change in trial product dose and/or other significant changes indicating a direct effect on the safety of the trial patients), the agreement of the competent ethics committee and/or authorities as well as of the patient to the protocol amendments will be obtained, and the amendment will be submitted to the German federal higher authority (BfArM).

20 References

1. Nehme, M., et al., *COVID-19 Symptoms: Longitudinal Evolution and Persistence in Outpatient Settings.* Ann Intern Med, 2021. **174**(5): p. 723-725.
2. Crook, H., et al., *Long covid-mechanisms, risk factors, and management.* Bmj, 2021. **374**: p. n1648.
3. Förster, C., et al., *Persisting Symptoms After COVID-19.* Dtsch Arztebl Int, 2022. **119**(10): p. 167-174.
4. Al-Aly, Z., Y. Xie, and B. Bowe, *High-dimensional characterization of post-acute sequelae of COVID-19.* Nature, 2021. **594**(7862): p. 259-264.
5. Nasserie, T., M. Hittle, and S.N. Goodman, *Assessment of the Frequency and Variety of Persistent Symptoms Among Patients With COVID-19: A Systematic Review.* JAMA Netw Open, 2021. **4**(5): p. e2111417.
6. Davis, H.E., et al., *Characterizing long COVID in an international cohort: 7 months of symptoms and their impact.* EClinicalMedicine, 2021. **38**: p. 101019.
7. Lund, L.C., et al., *Post-acute effects of SARS-CoV-2 infection in individuals not requiring hospital admission: a Danish population-based cohort study.* Lancet Infect Dis, 2021. **21**(10): p. 1373-1382.
8. Schulz, M.M., Sandra; Scherer, Martin; Karagiannidis, Christian; Czihal, Thomas, *Post-Acute Sequelae of SARS-CoV-2 Infection*

Characterization of Community-Treated Patients in a Case–Control Study Based on Nationwide Claims Data. Deutsches Ärzteblatt International, 2022. **119**(10): p. 177-178.
9. Yong, S.J., *Long COVID or post-COVID-19 syndrome: putative pathophysiology, risk factors, and treatments.* Infect Dis (Lond), 2021. **53**(10): p. 737-754.
10. Montefusco, L., et al., *Acute and long-term disruption of glycometabolic control after SARS-CoV-2 infection.* Nat Metab, 2021. **3**(6): p. 774-785.
11. McCaddon, A. and B. Regland, *COVID-19: A methyl-group assault?* Med Hypotheses, 2021. **149**: p. 110543.
12. Calderón-Ospina, C.A. and M.O. Nava-Mesa, *B Vitamins in the nervous system: Current knowledge of the biochemical modes of action and synergies of thiamine, pyridoxine, and cobalamin.* CNS Neurosci Ther, 2020. **26**(1): p. 5-13.
13. Elkon, K. and P. Casali, *Nature and functions of autoantibodies.* Nature Clinical Practice Rheumatology, 2008. **4**(9): p. 491-498.
14. Kazama, I., *Stabilizing mast cells by commonly used drugs: a novel therapeutic target to relieve post-COVID syndrome?* Drug Discov Ther, 2020. **14**(5): p. 259-261.
15. Myall, K.J., et al., *Persistent Post-COVID-19 Interstitial Lung Disease. An Observational Study of Corticosteroid Treatment.* Ann Am Thorac Soc, 2021. **18**(5): p. 799-806.
16. Goel, N., et al., *Systemic corticosteroids for management of 'long-COVID': an evaluation after 3 months of treatment.* Monaldi Arch Chest Dis, 2021. **92**(2).
17. Utrero-Rico, A., et al., *A Short Corticosteroid Course Reduces Symptoms and Immunological Alterations Underlying Long-COVID.* Biomedicines, 2021. **9**(11).
18. Bleidorn, J., et al. *Why do - or don't - patients with urinary tract infection participate in a clinical trial? A qualitative study in German family medicine*. German medical science : GMS e-journal, 2015. **13**, Doc17 DOI: 10.3205/000221.
19. BayFoNet. *Bayerisches Forschungsnetz in der Allgemeinmedizin,* https://bayfonet.de/.
20. FoPraNet-BW. *Forschungspraxennetz Baden-Württemberg,* https://www.forschungspraxennetz-bw.de
21. FOSA. *Fach- und Organspezifische Arbeitsgruppen des Netzwerks Universitätsmedizin,* https://fosa.netzwerk-universitaetsmedizin.de/.
22. NAPKON. *Nationales Pandemie Kohorten Netz,* https://napkon.de/.
23. DEGAM. *Deutsche Gesellschaft für Allgemeinmedizin und Familienmedizin,* https://www.degam.de/.
24. DEGAM, *SARS-CoV-2/Covid-19-Informationen & Praxishilfen für niedergelassene Hausärztinnen und Hausärzte, S2e-Leitlinie, AWMF-Register-Nr. 053-054*. 2022.

25. Buttgereit, F., et al., *Standardised nomenclature for glucocorticoid dosages and glucocorticoid treatment regimens: current questions and tentative answers in rheumatology.* Ann Rheum Dis, 2002. **61**(8): p. 718-22.
26. Klein, J., et al., *Distinguishing features of Long COVID identified through immune profiling.* medRxiv, 2022: p. 2022.08.09.22278592.
27. STADAPHARM, *Fachinformation Prednisolon STADA®(Zusammenfassung der Merkmale des Arzneimittels/SPC)*. 2021.
28. Authority, E.F.S., *TOLERABLE UPPER INTAKE LEVELS FOR VITAMINS AND MINERALS*. 2006.
29. Tanner, A., et al., *Dose-dependent prednisolone kinetics.* Clin Pharmacol Ther, 1979. **25**(5 Pt 1): p. 571-8.
30. Hevert, *Vitamin B-Komplex forte Hevert - Fachinformation*. 2008.
31. Health, N.I.o. *Vitamin B6 - Fact Sheet for Health Professionals*. 2022 02-06-2022 23-09-2022]; Available from: https://ods.od.nih.gov/factsheets/VitaminB6-HealthProfessional/.
32. DESAM-ForNet. *Initiative Deutscher Forschungspraxennetze; https://www.desam-fornet.de/*.
33. Di Blasi, Z. and J. Kleijnen, *Context Effects:Powerful Therapies or Methodological Bias?* Evaluation & the Health Professions, 2003. **26**(2): p. 166-179.
34. Di Stadio, A., et al., *Ultramicronized Palmitoylethanolamide and Luteolin Supplement Combined with Olfactory Training to Treat Post-COVID-19 Olfactory Impairment: A Multi-Center Double-Blinded Randomized Placebo- Controlled Clinical Trial.* Curr Neuropharmacol, 2022. **20**(10): p. 2001-2012.
35. Guslandi, M., *Steroid ulcers: Any news?* World J Gastrointest Pharmacol Ther, 2013. **4**(3): p. 39-40.
36. Innocenti F, Y.G.A., Ramirez J, Ratain MJ, *In vitro glucuronidation of prednisone.* Clinical Pharmacology & Therapeutics, 2005. **77**(2): p. 36-36.
37. Narum, S., T. Westergren, and M. Klemp, *Corticosteroids and risk of gastrointestinal bleeding: a systematic review and meta-analysis.* BMJ Open, 2014. **4**(5): p. e004587.
38. Venerito, M., et al., *Contribution of Helicobacter pylori infection to the risk of peptic ulcer bleeding in patients on nonsteroidal anti-inflammatory drugs, antiplatelet agents, anticoagulants, corticosteroids and selective serotonin reuptake inhibitors.* Aliment Pharmacol Ther, 2018. **47**(11): p. 1464-1471.
39. Masclee, G.M., et al., *Risk of upper gastrointestinal bleeding from different drug combinations.* Gastroenterology, 2014. **147**(4): p. 784-792.e9; quiz e13-4.
40. Fischbach, W., et al., *S2k-Guideline Helicobacter pylori and gastroduodenal ulcer disease.* Z Gastroenterol, 2017. **55**(02): p. 167-206.
41. Ratiopharm, *Pantoprazol-ratiopharm 40 mg magensaftresistente ® Tabletten - Fachinformation*. 2018.
42. Mebrahtu, T.F., et al., *Oral glucocorticoids and incidence of hypertension in people with chronic inflammatory diseases: a population-based cohort study.* Canadian Medical Association Journal, 2020. **192**(12): p. E295-E301.
43. Gulliford, M.C., J. Charlton, and R. Latinovic, *Risk of diabetes associated with prescribed glucocorticoids in a large population.* Diabetes Care, 2006. **29**(12): p. 2728-9.
44. Tamez-Pérez, H.E., et al., *Steroid hyperglycemia: Prevalence, early detection and therapeutic recommendations: A narrative review.* World J Diabetes, 2015. **6**(8): p. 1073-81.
45. Heckmann, J.G., *S2k Guideline: Therapie der idiopathischen Fazialisparese (Bell's palsy).* Deutsche Gesellschaft für Neurologie, 2022.
46. van der Goes, M.C., et al., *Monitoring adverse events of low-dose glucocorticoid therapy: EULAR recommendations for clinical trials and daily practice.* Annals of the Rheumatic Diseases, 2010. **69**(11): p. 1913-1919.
47. Schilling, I., et al. *Patients' and researchers' experiences with a patient board for a clinical trial on urinary tract infections*. Research involvement and engagement, 2019. **5**, 38 DOI: 10.1186/s40900-019-0172-0.

48. Bürgerforum. *Institut für Allgemeinmedizin, https://www.allgemeinmedizin.uni-wuerzburg.de/en/research/citizens-forum/*.
49. Salamanna, F., et al., *Post-COVID-19 Syndrome: The Persistent Symptoms at the Post-viral Stage of the Disease. A Systematic Review of the Current Data.* Front Med (Lausanne), 2021. **8**: p. 653516.
50. Kroenke, K., et al., *Minimally important differences and severity thresholds are estimated for the PROMIS depression scales from three randomized clinical trials.* J Affect Disord, 2020. **266**: p. 100-108.
51. Hermann, K., et al., *A brief patient-reported outcome instrument for primary care: German translation and validation of the Measure Yourself Medical Outcome Profile (MYMOP).* Health and Quality of Life Outcomes, 2014. **12**(1): p. 112.
52. Klok, F.A., et al., *The Post-COVID-19 Functional Status scale: a tool to measure functional status over time after COVID-19.* Eur Respir J, 2020. **56**(1).
53. Herdman, M., et al., *Development and preliminary testing of the new five-level version of EQ-5D (EQ-5D-5L).* Qual Life Res, 2011. **20**(10): p. 1727-36.
54. Kroenke, K., et al., *The PHQ-8 as a measure of current depression in the general population.* J Affect Disord, 2009. **114**(1-3): p. 163-73.
55. Chalder, T., et al., *Development of a fatigue scale.* J Psychosom Res, 1993. **37**(2): p. 147-53.
56. Zimmermann, P., Gondan, M. & Fimm, B, *Testbatterie zur Aufmerksamkeitsprüfung für Kinder KITAP. .* Herzogenrath: PSYTEST Verlag, 2002.
57. Meriem, M., et al., *Sit-to-stand test and 6-min walking test correlation in patients with chronic obstructive pulmonary disease.* Ann Thorac Med, 2015. **10**(4): p. 269-73.
58. Eldridge, S.M., et al., *CONSORT 2010 statement: extension to randomised pilot and feasibility trials.* Pilot Feasibility Stud, 2016. **2**: p. 64.
59. Thomann, A.E., et al., *The Montreal Cognitive Assessment: Normative Data from a German-Speaking Cohort and Comparison with International Normative Samples.* Journal of Alzheimer's Disease, 2018. **64**: p. 643 - 655.
60. Klempner, M.S., et al., *Treatment Trials for Post-Lyme Disease Symptoms Revisited.* The American Journal of Medicine, 2013. **126**(8): p. 665-669.
61. Cho, H.J., M. Hotopf, and S. Wessely, *The Placebo Response in the Treatment of Chronic Fatigue Syndrome: A Systematic Review and Meta-Analysis.* Psychosomatic Medicine, 2005. **67**(2): p. 301-313.

21 Signatures

The following people agree to the contents of the clinical trial and testify to this with their signature. Changes affecting the responsibility of any individual signatory must be reported promptly.

Sponsor/ Principal Coordinating Investigator and Principal Investigator Würzburg

Surname, first name (in block capitals)	Date, signature

Principal Investigator Trial Site Tübingen

Surname, first name (in block capitals)	Date, signature

Principal Investigator Trial Site Kiel

Surname, first name (in block capitals)	Date, signature
