# Supplementary material for: Low-dose prednisolone and B vitamins for post-COVID-19 syndrome in Germany: the PreVitaCOV randomised clinical trial
Source: eClinicalMedicine. 2026 Oct 24;101:104264. doi: 10.1016/j.eclinm.2026.104264 (PMC13720163; doi:10.1016/j.eclinm.2026.104264)
Supplement: Supplementary file 2 [file mmc3.pdf]

Prednisolone and vitamin B1, B6, and B12 in patients with Post-COVID-19-Syndrome (PC19S) – a randomized controlled trial in primary care

STATISTICAL ANALYSIS PLAN

Version 1.1

18.01.2024

Authors:
Uwe Malzahn

Study identifiers:

EudraCT No. 2022-001041-20

Table of Content

Statistical Analysis Plan ... 1
List of abbreviations ... 4
1 Administrative information ... 6
1.1 Study identifiers ... 6
1.2 Revision history ... 6
1.2.1 Contributors to the statistical analysis plan ... 6
1.2.2 Roles and responsibilities ... 6
1.2.3 Approvals ... 7
2 Study Synopsis ... 8
2.1 Study Objectives ... 8
2.1.1 Primary Objective ... 8
2.1.2 Secondary Objectives ... 8
2.2 Patient Population ... 9
2.2.1 Inclusion Criteria* ... 9
2.2.2 Exclusion criteria* ... 9
2.3 Endpoints ... 10
2.3.1 Primary Endpoint ... 10
2.3.2 Secondary Endpoints ... 11
2.3.3 Safety Endpoints ... 12
2.4 Intervention ... 12
2.5 Randomisation and Blinding ... 12
2.6 Sample Size ... 13
3 Statistical Analyses ... 14
3.1 General Principles ... 14
3.2 Interim Analysis ... 15
3.3 Multiplicity Adjustment ... 16
3.3.1 Post-Hoc Analysis within the primary analysis for the primary endpoint ... 16
3.3.2 Multiplicity adjustment within a secondary analysis of the primary endpoint referring to blinding assessment ... 16
3.4 Data Sets to be analysed ... 16
3.5 Subject Disposition ... 17
3.6 Patient Characteristics and Baseline Comparisons ... 17
3.7 Compliance to study Intervention(s) ... 18
3.8 Concomitant Therapies ... 19
3.9 Analysis of the Primary Endpoint ... 19
3.9.1 Main Analysis ... 20
3.9.2 Adjusted Analyses ... 21
3.9.3 Subgroup Analyses ... 21
3.9.4 Treatment of Missing Data ... 22

3.10 Other Sensitivity Analyses 23
3.11 Analysis of Secondary Endpoints 25
3.11.1 Sum scores for questionnaires as well as data from the VAS for QoL and NRS for pain 25
3.11.2 Ordinal secondary endpoints of Likert scale type 25
3.11.3 Physical exercise: 1-minute Sit-to-Stand-Test 25
3.11.4 Use of on-demand medication (from patient diary) 26
3.12 Analysis of Safety Endpoints 26
3.12.1 Number and Proportion of Patients with worsening PC19 Symptoms 26
3.12.2 Adverse Events 26
3.12.3 Serious adverse events 27
3.12.4 Laboratory data and vital signs 27
4 References 28
5 Examples for presented tables 29

List of abbreviations

**ANOVA**	Analysis of variance
**AE**	Adverse event
**COVID**	Coronavirus Disease
**CTCW**	Clinical Trial Center Würzburg
**DK**	Answer “I don’t know / I’m undecided about the treatment group” in the post-trial blinding questionnaire
**DSMB**	Data Safety Monitoring Board
**eCRF**	electronic case report form
**EQ-5D**	European Quality of Life 5 Dimensions
**EQ-VAS**	European Quality of Life visual analogue scale
**GP**	General Practitioner
**HMSS**	Health Measuring Scoring Service
**ICH**	International Council for Harmonization
**ICH E9**	Statistical principles for clinical trials of the ICH
**IMP**	Investigational Medicinal Product
**IIT**	Investigator-initiated trial
**ITT**	Intention to treat
**MI**	Multiple Imputation
**MoCA**	Montreal Cognitive Assessment
**NSAID**	Nonsteroidal Anti-inflammatory Drugs
**NRS**	Numeric Rating Scale
**PC19S**	Post-COVID-19-Syndrome
**PCFS**	Post-COVID-19 Functional Status Scale
**PCR**	Polymerase chain reaction
**PHQ8**	Eight-item Patient Health Questionnaire depression scale
**PI**	Principal investigator
**PP**	Per protocol
**PROMIS**	Patient Reported Endpoints Measurement Information System
**RCT**	Randomised controlled trial
**SAE**	Serious adverse event
**SAP**	Statistical analysis plan
**SAR**	Serious adverse reaction
**SD**	Standard deviation
**SM**	Standard deviation of means
**SUSAR**	Suspected unexpected serious adverse reaction
**TAP**	Testbatterie zur Aufmerksamkeitsprüfung

**UADR**	Unexpected adverse reaction

1 Administrative information

1.1 Study identifiers

- Protocol: 3.0, 17.10.2022 (Approved: 06.10.2022 ethic committee; 14.10.2022 competent authority)
- Protocol Amendment 1: 4.0, 20.03.2023 (Approved: 02.05.2023 ethic committee; 05.06.2023 competent authority)
- Protocol Amendment 2: 5.0, 28.08.2023 (Approved: 05.10.2023 ethic committee; 04.10.2023 competent authority)
- EudraCT No. 2022-001041-20
- Protocol code of the Sponsor: F001AM0222_1

1.2 Revision history

Version	Date	Changes made to document	Authors
1.1			

1.2.1 Contributors to the statistical analysis plan

1.2.2 Roles and responsibilities

Names and OCRID	Affiliation	Role on study	SAP contribution
Uwe Malzahn	University Hospital Würzburg, Center for Clinical Trials	Trial statistician	Development of the SAP

1.2.3 Approvals

The undersigned have reviewed this plan and approve it as final. They find it to be consistent with the requirements of the protocol as it applies to their respective areas. They also find it to be compliant with ICH-E9 principles and, in particular, confirm that this analysis plan was developed in a completely blinded manner (i.e. without knowledge of the effect of the intervention(s) being assessed).

< Prof. Dr. med. Ildikó Gágyor >

	<signature>	<date>

<Dr. rer. nat. Uwe Malzahn>

	<signature>	<date>

2 STUDY SYNOPSIS

This is a Phase IIIb, multicenter, randomised, double blinded, placebo-controlled study with factorial ANOVA design and four parallel groups.

Flow Chart

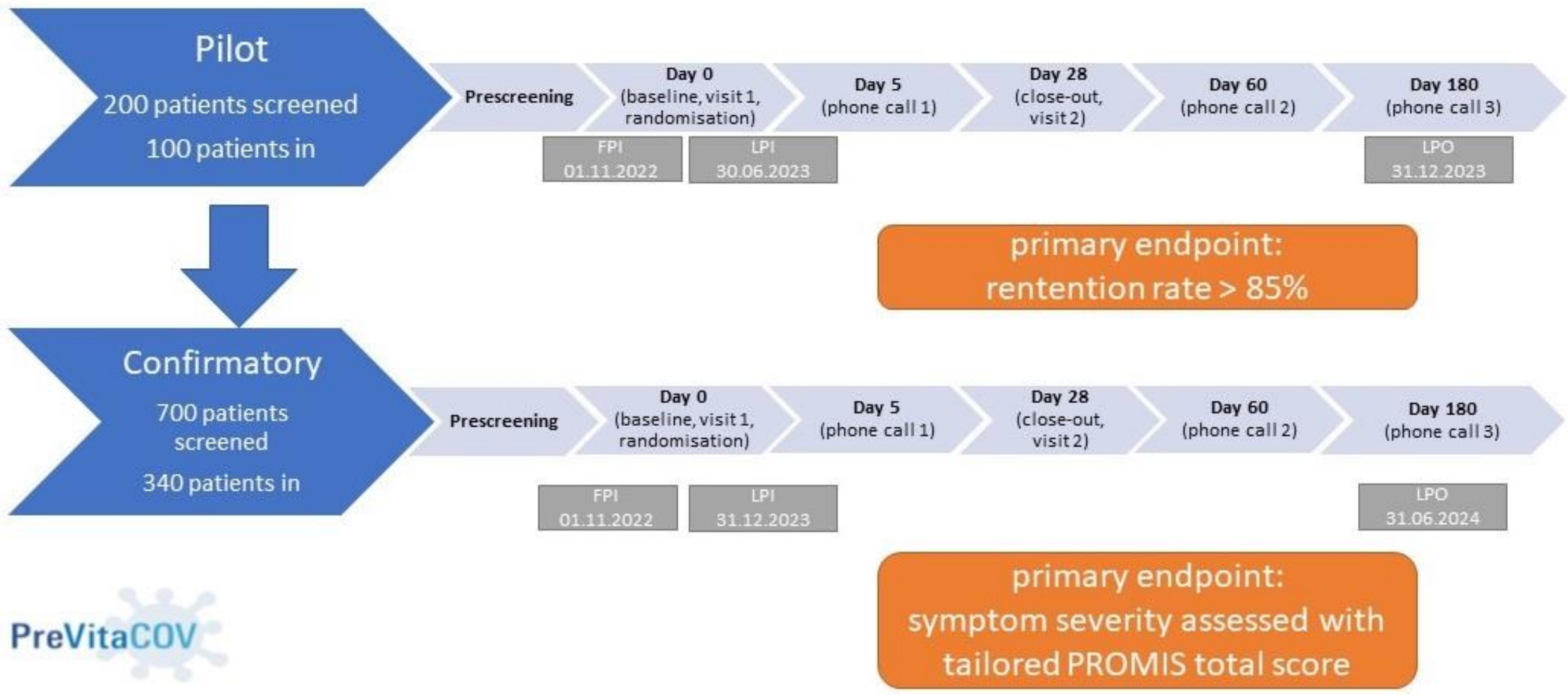

*for women with childbearing potential an additional pregnancy test is performed on day 32 (+ 2)

2.1 STUDY OBJECTIVES

2.1.1 PRIMARY OBJECTIVE

The primary objective of the Pilot study will be feasibility and acceptance of screening and recruitment in primary care, as assessed by the retention rate at day 28.

The primary objective of the confirmatory study will be to assess the effectiveness of treating patients with PC19S in primary care with prednisolone and/or vitamin B1, B6, and B12 in a fixed combination as change in symptom severity to day 28.

2.1.2 SECONDARY OBJECTIVES

The secondary objectives of the confirmatory study will be to assess the effectiveness of treating patients with PC19S in primary care with prednisolone and/or vitamin B1, B6, and B12 in a fixed combination in terms of the change to day 28 in other measures of symptom severity as well as in terms of the change in health related quality of life, depression, fatigue, pain, cognitive function, physical capacity and in terms of the use of on-demand medication and concomitant medication.

2.2 Patient Population

2.2.1 Inclusion Criteria*

- adult patients

  history of SARS-CoV-2 infection at least 12 weeks ago (if patients had more than one infection with SARS-CoV-2 the 12 weeks refer to the infection causing the Post-COVID-19 symptoms); the infection must be documented by either a positive PCR- or Antibody-Test or be confirmed by the patient's GP on the provided sheet to confirm Post-COVID-19 diagnosis, pre-existing conditions, and prior medication.
- symptoms concerning at least one of the following domains: fatigue, dyspnea, cognition, anxiety, depression
- symptoms that developed during or after the SARS-CoV-2 infection, that persist until study inclusion and that are associated with COVID-19 (the latter will be assessed by the patient's GP or the local investigator)

2.2.2 Exclusion criteria*

- acute COVID-19 at baseline visit (rapid SARS-CoV-2 antigen test)
- patients who were treated in the intensive care unit because of COVID-19
- pregnancy/breastfeeding
- diabetes mellitus
- PC19S symptoms can be explained by an alternative diagnose (e.g., chronic fatigue syndrome, depression, active or preceding cancer therapy, severe anemia, sleep apnea syndrome) as assessed by the patients' GP or the investigator (responsible physician)
- <u>History of severe</u> medical conditions such as
  - concomitant acute infectious disease
  - gastrointestinal ulcer
  - liver disease/ liver cirrhosis
  - malabsorption or condition after bariatric surgery
  - chronic airway disease (e.g., asthma, COPD)
  - chronic heart failure (NYHA III or IV)
  - neurologic disorders (e.g., multiple sclerosis, motoneuron disease)
  - untreated hypothyroidism
  - significantly impaired glucuronidation (e.g., Gilbert-Meulengracht, ROTOR, or Crigler-Najjar syndrome)
  - immunodeficiency or a chronically weakened immune system (e.g., HIV, AIDS, lymphoma, chemo-radio-therapy, immunosuppressive pathology)
  - mental disorders (e.g., depression, psychosis, dementia)
  - active cancer
  - any other severe medical conditions that preclude participation as determined by responsible physician

* refer to the last protocol version

- current use of
  - immunosuppressive drugs
  - non-steroidal anti-inflammatory drugs (NSAID), ASS, Indometacin
  - fluoroquinolones
  - anticoagulation: phenprocoumon or other cumarin derivates, direct oral anticoagulants
  - any other drug with a possible interaction that could exhibit clinically relevant interactions with the study medication (as described in Fachinformation Prednisolon STADA®, Predni H Tablinen® Zentiva or Fachinformation Vitamin B komplex Hevert). The decision regarding the clinical relevance of the interactions is at the discretion of the clinical investigator
- systemic treatment with prednisolone for at least 7 days since COVID-19 or any parenteral application since the end of the acute phase of COVID-19; treatment with vitamins B1, B6, or B12 in doses equivalent to the dose of the study medication for at least 7 days or any parenteral application since the end of the acute phase of COVID-19; vitamin supplements containing vitamin B1, B6, or B12 should have been ceased at least 4 weeks prior to the inclusion in the study
- known allergy and contraindications to the intervention drugs
- need of care and/or peer dependency
- nursing home residents
- inability to understand the scope of the study, to follow study procedures and to give informed consent or to attend the study sites
- participation in another interventional trial at the same time or within the past 3 months before enrollment
- Female patients considering to get pregnant during the trial and within 1 week after the last dose of study drug(s)

2.3 Endpoints

2.3.1 Primary Endpoint

The trial is divided into two phases:

In a pilot study, we will evaluate feasibility and safety of treating PC19S with Prednisolone and/or Vitamin B1, B6, and B12 in comparison to placebo in patients recruited by their GPs.

- The primary endpoint of the Pilot study will be feasibility and acceptance of screening and recruitment in primary care, as assessed by the retention rate at day 28. That means the proportion of patients who completed the 4-week intervention.

If the pilot phase shows feasibility, the study will be transferred into a confirmatory study to determine the effectiveness of the interventions.

- The primary endpoint of the confirmatory study will be the change in symptom severity to day 28 as assessed by a specifically tailored total score based on the patient reported outcome measurement information system (PROMIS). That means the primary endpoint is a change score, the difference in our specifically tailored outcome measure from baseline (visit 1) to visit 2 after 28 days:
  Tailored-score (V2) – Tailored-score (V1).

2.3.2 Secondary Endpoints

In the case that after analysis of the first 100 patients the decision will be not to proceed from the pilot study to the confirmatory study the following secondary endpoints are defined for **the final analysis at this stage**

- severity of PC19 Symptoms:
  - tailored PROMIS total score and subscores in the domains included in the tailored PROMIS total score
  - MYMOP
  - PC19S functional status (PCFS - Scale)
  - symptom list
- health related quality of life (EQ-5D-5L and visual analogue scale)
- depression (PHQ 8)
- fatigue (Chalder fatigue Scale)
- pain (numeric rating scale for pain[3] (NRS))
- cognitive function (alertness, divided attention, distractibility, flexibility and visual scanning[2] (TAP)1)
- physical fitness (1 Minute Sit-to-Stand-Test)
- use of on-demand medication and change in concomitant medication (patient diary)
- feasibility and acceptance (qualitative interviews with a subgroup sample, questionnaire)

The secondary endpoints for **the confirmatory study** will be

- severity of PC19 Symptoms:
  - PROMIS subscores in the domains included in the tailored PROMIS total score
  - MYMOP
  - PC19S functional status (PCFS - Scale)
  - symptom list
- health related quality of life (EQ-5D-5L and visual analogue scale)
- depression (PHQ 8)
- fatigue (Chalder fatigue Scale)
- pain (numeric rating scale for pain[3] (NRS))
- cognitive function (alertness, divided attention, distractibility, flexibility and visual scanning[2] (TAP))
- physical fitness (1 Minute Sit-to-Stand-Test)
- use of on-demand medication and change in concomitant medication (patient diary)
- feasibility and acceptance (self-developed questionnaire)

[1] https://www.psytest.net/de/testbatterien/tap-m/zielsetzung

[2] protocol version 3.0 was incorrect: sustained attention was not tested, it was always tested for flexibility and visual scanning

[3] protocol version 3.0 was incorrect: pain was not tested by numeric rating scale, it was always tested by numeric rating scale

2.3.3 SAFETY ENDPOINTS

- Adverse events by system organ class day 0 until month 2 (phone call 2)
- Severe adverse events by system organ class day 0 until month 2 (phone call 2)
- Proportion of patients with at least one AE until day 60 (phone call 2)
- Number of patients with worsening symptoms 2 months after inclusion (phone call 2)

2.4 INTERVENTION

Administration of vitamin B1 (thiamine), B6 (pyridoxine), and B12 (cyanocobalamin) and/or prednisolone in primary care patients with PC19S.

Trial drug 1: Prednisolone. The dosage will be Prednisolone 20 mg for five days and subsequently 5 mg for 23 days (both 1x1 capsule / day).

Trial drug 2: Vitamin B compound. Vitamin B1 (100 mg), B6 (50 mg), and B12 (500 µg) for 28 days.

Treatment group	Day 1 – Day 5	Day 6 – Day 28
1st arm: prednisolone and vitamin B compound placebo	prednisolone (20mg) and vitamin B compound placebo	prednisolone (5mg) and vitamin B compound placebo
2nd arm: prednisolone placebo and vitamin B compound	prednisolone placebo and vitamin B compound (100 mg B1, 50 mg B6, 500 µg B12)	prednisolone placebo and vitamin B compound (100 mg B1, 50 mg B6, 500 µg B12)
3rd arm: prednisolone and vitamin B compound	prednisolone (20mg) and vitamin B compound (100 mg B1, 50 mg B6, 500 µg B12)	prednisolone (5mg) and vitamin B compound (100 mg B1, 50 mg B6, 500 µg B12)
4th arm: prednisolone placebo and vitamin B compound placebo	prednisolone placebo and vitamin B compound placebo	prednisolone placebo and vitamin B compound placebo

The study intervention per patient will take 28 days followed by an observation period of 5 months after the end of study treatment. The individual patient study duration is 6 months.

2.5 RANDOMISATION AND BLINDING

Patients are allocated to the 4 treatment groups via stratified block randomisation with varying block size. The single stratification factor is the study center. With this method of random treatment assignment, we try to avoid predictability, selection bias and full or partial unblinding still accomplish an acceptable balance. The randomisation lists is generated outside the secuTrial ® and then imported to secuTrial ®.

This study is designed as a double-blind trial. Therefore, neither patients nor clinicians will know the study arm they are in. This will be achieved by an identical appearance of the tablets containing Vitamin B or placebo and identically encapsulated tablets for prednisolone or placebo (double dummy design).

2.6 SAMPLE SIZE

<u>**Sample size calculation for the pilot study**</u>

A total of 100 patients will be enrolled for the pilot study. When this stage indicates feasibility (defined as a retention rate of at least 85% until day 28 or according to a recommendation of the DSMB), the trial will be transformed into a confirmatory study to investigate effectiveness of the treatments.

The aim of the sample size calculation for the pilot study was to ensure that we could estimate the retention rate - that means proportion of patients who completed the 4-week intervention, with sufficient precision. Sufficient precision here means that the width of the corresponding 95% confidence interval not exceeds 0.15 (15%). Conservatively we base the calculation on the exact (Clopper-Paerson) method. Furthermore, because the target retention rate in this trial is 0.85 (85%) we assume for the calculation an observed sample proportion of 0.85. Then 100 patients will be needed to yield a confidence interval with the postulated precision.

According to the study design 4 equal sized treatment groups are planned. Therefore 4*25 = 100 patients are to be randomised and analysed in a 1:1:1:1 ratio at the beginning. This means in turn that n=200 are to be assessed for eligibility and n=100 are to be allocated to trial.

<u>**Summary of sample size calculation for the confirmatory study**</u>

The aim is the detection of an underlying main effect corresponding to at least 3-points (T-score) on the outcome scale. The outcome scale is the scale for the changes in our primary endpoint from baseline to day 28. The primary outcome measure is a tailored weighted mean T-score from 5 PROMIS subscales.

The range of values for the primary outcome measure is 16.7 – 57.7

In the case that this 3-point difference is the smaller one of the two underlying main treatment effects (vitamin B compound and prednisolone) the sample size will ensure that both effects will be detected with sufficient power (80%). In the case that this 3-point difference is the stronger one of the two underlying main treatment effects the sample size will ensure with sufficient power that at least this stronger main effect will be detected.

Within the primary analysis four treatment groups are compared: patients with neither intake of vitamin B compound nor prednisolone, patients with intake of vitamin B compound only, patients with intake of prednisolone only and patients with intake of both vitamin B compound and prednisolone combined.

We base the sample size calculation of the primary analysis for the primary endpoint by a two-factor analysis of variance (ANOVA). The dependent variable within the ANOVA model is our primary endpoint, operationalized as the change in the tailored PROMIS score from baseline to day 28 (visit 2). Furthermore, the two-factor ANOVA model contains the two main factors vitamin B compound and prednisolone, both with two factor levels: intake no or yes. Finally, the model contains the interaction factor with four levels. Within this model we will estimate the main- and interaction effects and test the two null hypotheses of no difference between the two-factor level means in each case (for the main factors vitamin B compound and prednisolone) and test the null hypothesis of no interaction between the factors vitamin B compound and prednisolone by the corresponding F-tests with one nominator degree of freedom in each case.

<u>Assumptions for sample size calculation</u>: K. Kroenke[50] et al. reported a difference in T-score of 3 to 4 points as a **reasonable minimally important difference** for the PROMIS depression scales. Considering this for our primary outcome measure, as a weighted mean of T-scores from 5 PROMIS short form instruments, we aim to detect such a difference of 3 points as a significant effect. The same seems to be reasonable for change scores, that means for our primary endpoint. A 3-point difference between the effects of the two

level (*no intake* versus *intake yes*) for a main factor within the linear model for a two-factor experiment corresponds to a standard deviation of means (SM) of 1.5.

Conservatively, we claim that the sample size has to be adequate in this sense that **any** underlying main effect corresponding at least to a standard deviation of means (SM) of 1.25 can be detected as a significant deviation from the null hypothesis of no effect with a power of 80% by the corresponding F-test at a significance level α = 0.05. Furthermore, we assume a standard deviation (SD) of 7.5 points for the primary endpoint variable (see section 3.9 Analysis of the primary endpoint). With this expressed in terms of **effect sizes**, the assumptions above correspond to an effect size about 0.167 (precisely 1.25/ 7.5).

A sample size of 4*72 = 288 ensures a power of 80% to detect any main effect with an effect size of at least 0.167 as significant deviation from the null hypothesis of no effect.

Assuming a lost to follow up rate of 15%, **4*85 = 340** patients are to be included in the study.
<u>Remark:</u> The difference between the two underlying marginal means for one factor (vitamin B compound or prednisolone) depends additionally on the strength of interaction effects!

3 Statistical Analyses

3.1 General Principles

Before any statistical analyses the entered data will be checked for plausibility and completeness.
The primary objective of the Pilot study will be feasibility and acceptance of screening and recruitment in primary care, as assessed by the retention rate at day 28. That means the proportion of patients who completed the 4-week intervention is the primary endpoint of the pilot study. For this endpoint point and interval estimates (95% confidence interval by the Wilson score method) will be given.
If the criteria to continue (sufficient retention rate or recommendation by the DSMB) are met, any other data analysis at this stage, beyond that of the primary endpoint, will be restricted to combined analyses of all four study groups describing baseline patient characteristics. Patients enrolled in the pilot study will be included in the analyses of the confirmatory study.
Regarding primary, secondary and safety endpoints of part 2, the confirmatory study, see chapter "2.3 endpoints".

For all secondary endpoints listed in section 2.3.2 the corresponding total sum scores and if applicable subscores will be calculated for the different visits together with the corresponding change scores from baseline to day 28. Where appropriate, convenient transformations will be made.

Generally, all study variables will be presented using the appropriate descriptive statistics according to the measurement scale of data and for interval and ratio scaled data, respectively, according to the properties of the observed distributions (e.g., outlier values, skewness). Measures of frequency, of central tendency and dispersion or variation will be given as appropriate.

That means, for continuous variables the number of missing and of non-missing observations, arithmetic mean and/or median, range, SD or interquartile range, minimum, maximum. Frequency tables will be used to summarize categorical variables for the 4 treatment groups at different points in time. If appropriate, shift (change from baseline) tables will be given. For binary variables, numbers of missing and non-missing observations will be given together with the corresponding proportions. Shift tables (change from baseline) will be given if possible and appropriate.

In exploratory analyses, factors (such as trial center, age, symptom duration or laboratory parameters) potentially influencing the primary outcome will be investigated using appropriate multivariable modelling.

All secondary outcomes will be analyzed in exploratory analyses.

Safety outcomes will be analyzed by reporting frequencies (percentages) of (S)AEs as well as the number of patients with worsening symptoms for each group.

For selected secondary endpoints (T-scores from the 5 PROMIS Short Form instruments forming the primary endpoint (Fatigue 4a, Dyspnoea Severity 10a, Cognitive Function 4a, Emotional Distress-Anxiety 4a and Emotional Distress-Depression 4a) as well as the EQ-VAS), secondary analyses will be carried out for the corresponding change scores from baseline to day 28 in the same manner as for the primary endpoint by a 2x2 factorial analysis of variance.

Statistical methods for the analyses of changes from baseline to day 28 in data from multiple response type resp. "check-all-that-apply" questionnaire items (e.g. physical examination: auscultation of chest and heart, orientating neurological examination) depend on measurement scales (interval, ordinal, nominal, dichotomous) and data types (categorical, counts, numerical, continuous, ratios) in each case. Descriptive analysis may comprise appropriate summarizing statistics and shift tables. Confirmatory analysis resp. model-based analysis may comprise parametric Analysis of Variance (ANOVA), the nonparametric Kruskal-Wallis test, parametric analysis of covariance (ANCOVA) resp. Nonparametric Randomisation-Based Analysis of Covariance (Koch et al. 1998). For ordinal variables resp. Likert-scale data hierarchical models will be applied (SAS: GLIMMIX, NLMIXED). When using Ordinal Response Mixed Models we will firstly check if cumulative link models are applicable, that means whether the proportional odds condition is met. If the assumption is violated hierarchical multinomial models will be used. In this case nonparametric sensitivity analyses will be done as shift analyses with pairwise comparisons of the 4 treatments. For this firstly shifts from baseline (visit 1) to visit 2 after 28 days will be calculated. These shifts will be analysed by the Cochran-Mantel-Haenszel test, stratified by the baseline score.

Planned statistical analyses will be done using the current SAS version (SAS Institute Inc., Cary, NC, USA) as well as the current IBM SPSS version.
Item response theory (IRT) scoring provided T-scores for the 5 short-form PROMIS subscales were provided by the Health Measure Scoring Service (HM-SS). For this the Institute of General Practise at the UKW will upload the corresponding completed templates with the raw short-form PROMIS data to the HM-SS homepage. The received scored data, that means the T-scores for the 5 short-form PROMIS instruments for the patients at the different visits, will be delivered by the Institute of General Practise to the Clinical trial Center Würzburg for the statistical analysis according this SAP.

3.2 Interim Analysis

Firstly a total of 100 patients will be enrolled for the pilot study. After completion of the 28-day visit for these 100 patients, the decision will be made whether to proceed from the Pilot Study to the confirmatory study.

When this stage indicates feasibility (defined as a retention rate of at least 85% until day 28 or according to a recommendation of the DSMB), the trial will be transformed into a confirmatory study to investigate effectiveness of the treatments.

The observed retention rate will be one component within the criteria for continuing to the confirmatory study, the confirmatory part comparing the effectiveness of treatments. For example, the sample size of

100 allows an underlying retention rate of 0.85, which is considered as an acceptable retention rate here, to be distinguished from an underlying retention rate of 0.75 with power 78% at a significance level 0.05 and from an underlying retention rate of 0.73 with power of 90%.

We do not want to base the decision of whether to continue with the confirmatory study exclusively on a formal statistical criterion, so we calculated the sample size for the pilot study based on the precision of the estimate, not on a procedure of sequential testing.

3.3 Multiplicity Adjustment

3.3.1 Post-Hoc Analysis within the primary analysis for the primary endpoint

Definition: let us denote by $\boldsymbol{\mu}_{ij}$ the population means of the primary endpoint in the study, the change in the tailored PROMIS score from baseline to day 28 (visit 2), i, j = 1,2.

Specifically let us denote:

μ_{11}: population mean for patients under placebo

μ_{12}: population mean for patients without intake of vitamins but with intake of prednisolone

μ_{21}: population mean for patients with intake of vitamins but without intake of prednisolone

μ_{22}: population mean for patients with intake as well of vitamins as of predisolone.

Within the primary analysis firstly we test the two main effects and the interaction effect and estimate the corresponding effect sizes.

We can rewrite the null hypotheses in the 2x2 factorial ANOVA in terms of the underlying cell means for:

Main factor vitamins : $\mu_{11} + \mu_{12} = \mu_{21} + \mu_{22}$

Main factor prednisolone : $\mu_{11} + \mu_{21} = \mu_{12} + \mu_{22}$

Interaction : $\mu_{12} - \mu_{11} = \mu_{22} - \mu_{21}$.

In the case that the interaction proved to be statistically significant we test the six simple effects, that means we test the six null hypotheses

$\mu_{11} = \mu_{12}$, $\mu_{11} = \mu_{21}$, $\mu_{12} = \mu_{22}$, $\mu_{21} = \mu_{22}$, $\mu_{11} = \mu_{22}$ and $\mu_{12} = \mu_{21}$.

For these multiple, pairwise comparisons we use the Tukey HSD test (if actually all group sizes equal) resp. the Tukey-Kramer test (if there are unequal group sizes because of drop-outs) to control the familywise error rate (FWER) at the level $\alpha = 0.05$. These tests are more powerful than e.g. the Bonferroni, Šidák or Scheffé test.

3.3.2 Multiplicity adjustment within a secondary analysis of the primary endpoint referring to blinding assessment

See paragraph "Blinding assessment and interpreting data from questionnaires in the case of imperfect blinding". We define the planned 9 multiple, pairwise comparisons addressing the problem of possibly imperfect blinding as one family of tests and use the Tukey-Kramer test to control the familywise error rate (FWER) at the level $\alpha = 0.05$. This test is more powerful than e.g. the Bonferroni, Šidák or Scheffé test.

3.4 Data Sets to be analysed

The primary analysis will be conducted according to the Intention-to-Treat (ITT) Principle within the Full Analysis Set (FAS). That means the treatment assigned to a patient for the analysis is the treatment

according to the randomisation in each case.

Full Analysis Set

As the purpose of this trial is to compare treatment strategies the Full Analysis Set contains all randomised patients with drug assignment designated according to the initial randomisation regardless of whether or not they have taken the trial medication.

Per Protocol Analysis Set

The Per Protocol Analysis Set consists of all patients in the Full Analysis Set with protocol-compliant ingestion of the trial medication, full documentation of the symptom scores within the first 28 days and no serious protocol violations, in accordance with the blinded assessment of the steering committee.

3.5 Subject Disposition

The following data will be given in table form:

- How many patients were screened and how many patients were randomised
- How many patients dropped out
    - Because of death
    - Because of withdrew consent
    - Because of loss to follow-up
    - Because of significant non-compliance
    - Because of adverse events leading to substantial changes in the individual risk-benefit considerations that suggest a
    - discontinuation of the study drug
    - Because of pregnancy

Furthermore the distribution of patients on the centres will be given and graphical representations of the course of recruitment by months will be given.

3.6 Patient Characteristics and Baseline Comparisons

We will summarize data by descriptive statistics according to the measurement level of the corresponding variables. For variables of categorical nominal level we will present in table form for the four treatment groups the number and proportion of observations in each category of the variable. For variables of categorical ordinal level with few possible values, e.g. data from Likert-type scales, we will proceed just as for nominal data, but explicitly maintaining the order of categories. Variables of categorical ordinal level with many possible values will be treated as numeric (see below). For variables at numeric interval or numeric ratio level we will also take into account the characteristics of the empirical distribution of the observed values, that means skewness, possible outlier values, possible gaps in distribution and possible bi-or multimodal distributions. As measures of location resp. central tendency we will use the arithmetic mean and/or the median. As measures of dispersion we will use the standard deviation and/or the values of the first and third quartile. In any case the minimum and maximum value will be given.
Descriptive statistics for the following baseline data will be separately listed for the 4 treatment groups:

- Sociodemographic data
    - Gender
    - Age
    - Family status
- Education
- Employment

- Mental fitness (MoCA)
- Physical fitness (1 Minute Sit-to-Stand-Test)
- Duration of Post-COVID-19 symptoms (weeks)
- Vaccination against SARS-CoV-2
- Endpoint variables:
  - Tailored PROMIS total score
  - 5 PROMIS® Short Form instruments: Fatigue 4a, Dyspnoea Severity 10a, Cognitive Function 4a, Emotional Distress-Anxiety 4a and Emotional Distress-Depression 4a.
  - MYMOP
  - PC19S functional status (PCFS - Scale)
  - health related quality of life (EQ-5D-5L and visual analogue scale)
  - depression (PHQ 8)
  - fatigue (Chalder fatigue Scale)
  - pain (numeric rating scale for pain (NRS))
  - cognitive function: Alertness, divided attention, distractibility, flexibility and visual scanning (TAP)
  - physical fitness (1 Minute Sit-to-Stand-Test) and physical examination (auscultation of chest and heart, orientating neurological examination, lymph node status)

Furthermore descriptive baseline comparisons will be done between two patient groups defined by the kind of confirmation for the inclusion criteria “history of SARS-CoV-2 infection at least 12 weeks ago”:

- by a positive PCR or Antibody-Test or
- confirmed by the patient’s general practitioner (from amendment 1/protocol V4.0 on included)

These comparisons will be done for the endpoint variables (see above) as well as for mental fitness, physical activity, duration of Post-COVID-19 symptoms (weeks) and vaccination against SARS-CoV-2.

3.7 Compliance to study Intervention(s)

When patients receive the trial drug, they will be carefully instructed in how to handle and store the medication. This includes being told that any leftover medication and the empty pack must be returned to the trial site.

Compliance will be checked and documented on day 28 (visit 2) considering the empty pack or any residual medication returned to the trial site.

The dispensing and return of the trial product to the trial site will be documented in the Drug Accountability Log in the investigator site file (identifying the patient, the date and quantity of medication dispensed, the date and quantity of medication returned to the investigator, the date and quantity of medication returned to the study centers).

Drug accountability will be captured by the number of capsules and tablets left per box and day.

By means of information from documented patients specific end of trial (possibly early termination), drug accountability as well as patient diary the proportion of days with protocol-compliant intake of tablets of the total intended treatment duration will be calculated for each patient. At this the total intended treatment duration is 28 days or the length from baseline to possible early study termination before day 28. After checking for possible serious violations from the assumptions of homoscedasticity and normal distribution the data will be analyzed by two-way ANOVA (see primary analysis of the primary endpoint) or by Kruskal Wallis test.

3.8 Concomitant Therapies

Use of on-demand medication and change in concomitant medication will be collected in the patient diary. Patients will complete a diary noting the intake of any medication in addition to their usual daily medication (medication on demand and any change in concomitant medication) from day 1 to day 28.

Analysis for the secondary endpoint use of on-demand medication (patient diary): to compare the counts of on-demand medications within the period from baseline to day 28 between the four treatments negative binomial regression (NBR) models resp. zero-inflated negative binomial regression (ZINBR) models will be used. Within these models the count of using additional on-demand medication is the dependent variable, furthermore the models include the two binary main factors treatment with vitamin B compound, treatment with prednisolone as well the interaction factor. Using NBR models we can account for possible over-dispersion and using ZINBR models we can account for a possible elevated frequency of zero accounts in addition.

3.9 Analysis of the Primary Endpoint

The intention for the primary endpoint of the pilot study was to evaluate the feasibility and acceptance of screening and recruitment in primary care as assessed by the retention rate at day 28. Point and interval estimations (95% CI, confidence interval) will be given for the retention rate.
For the primary analysis the retention rate is defined as the proportion of randomised patients who completed the visit V5, the 28-day visit.
For a secondary analysis we consider a more restrictive criterion: the proportion of patients completing the 28-day visit with correct intake of study medication. At this the criterion for a correct intake of study medication is: according to the drug accountability log at least 26 tablets and at least 26 capsules were taken within the period from randomisation to the 28-day visit. According to the study protocol the intake of 28 tablets and 28 capsules is planned, 26 from 28 tablets (capsules) corresponds to more than 90% of the planned amount.

The intention for the primary endpoint of the confirmatory study was to construct a tailored measure for the disease burden of patients with Post-COVID-19-Syndrome. This should be a patient reported outcome not too burdensome for patients. We operationalized this as a mean of the T-scores from 5 PROMIS® Short Form instruments:

- PROMIS Short Form v1.0 – Fatigue 4a
- PROMIS Short Form v1.0 – Dyspnoea Severity 10a
- PROMIS Short Form v2.0 – Cognitive Function 4a
- PROMIS Short Form v1.0 – Emotional Distress-Anxiety 4a
- PROMIS Short Form v1.0 – Emotional Distress-Depression 4a

At this, we have to distinguish between negatively-worded concepts (like Fatigue, Dyspnoea, Anxiety and Depression) and positively worded concepts like Cognitive Function. Therefore, the outcome measure isn't a simple mean but a linear combination adding up the 4 T-scores for the 4 PROMIS Short Forms Fatigue, Dyspnoea Severity, Anxiety, Depression and subtracting the T-score for Cognitive Function.

Mean and SD for all T-scores of the five validated instruments are 50 and 10 within the reference population from the population 2000 General US census. Therefore, by construction above the mean of our primary outcome measure in the reference population will be 30. But we don't know the variance and with this don't know the SD in the reference population. For this, we would need to know all 10 pairwise covariances resp. all 10 pairwise correlation coefficients in the reference population between the 5 T-scores. But we can get a very rough approximation: if we assume for all 10 pairs of the 5 T-scores a medium strength of correlation, quantified by a Pearson correlation coefficient between 0.4 and 0.5, the SD of our transformed Post-COVID-19 tailored PROMIS total score will be between 7 and 8 (rather conservative).

Our primary outcome measure in the confirmatory study is defined as a change score, the difference in our specifically tailored outcome measure from baseline (visit 1) to visit 2 after 28 days:

Tailored-score (V2) – Tailored-score (V1).

Assuming a medium strength for the correlation between baseline measurements and measurements 28 days after baseline, we assume for this change score roughly a SD between 7 and 8 points too, justified by experience gained from earlier change score analyses.

3.9.1 MAIN ANALYSIS

Data analysis for the primary endpoint of the pilot study: retention rate at day 28

For the first endpoint of the pilot study, the proportion of patients who completed the 4-week intervention, point and interval estimations (95% CI, confidence interval) will be given. This will be done combining all 4 treatment groups

Data analysis for the primary endpoint of the confirmatory study: the specifically tailored PROMIS total score, derived from 5 Short Form instruments

- The following will be analysed:Primary endpoint: mean change in the tailored score from baseline to day 28 (visit) with 95% CI within the 4 treatment groups
- Summarizing descriptive statistics at baseline and day 28 (visit 2) for the tailored score within the 4 treatment groups
- **Primary analysis:** Factorial ANOVA for the dependent variable change in the tailored score from baseline to day 28 (visit 2)
- Firstly we will simply check whether two general assumptions for the two-way ANOVA have been violated. For this we will use two diagnostic plots: a normal probability plot to evaluate the normality assumption, and a scale-location plot to evaluate the constant variance assumption.

Remark: But it is well known that generally in the case of equal sample sizes (means equal group sizes) F-tests in ANOVA are very robust (in the sense of type I error rates) against violations of the normal distribution assumption. Recent Monte Carlo simulation studies revealed that this is also the case for moderate violations of the common variance assumption.

Problems investigated by corresponding F-tests within a model of a 2-factor ANOVA (factorial design):
Are there differences in the mean change score among patients treated with and without vitamin B compound?

- Null hypothesis: there is no effect for administration of vitamin B compound
  - Are there differences in the mean change score among patients treated with and without prednisolone?
- Null hypothesis: there is no effect for administration of prednisolone
  - Are there any vitamin-B-compound-prednisolone combinations that exhibit a mean change score that cannot be explained by administration of vitamin B compound and/or administration of prednisolone alone?
- Null hypothesis: there is no such interaction effect.

Further analysis within the 2-factor ANOVA:

- Post-Hoc Analysis within the primary analysis for the primary endpoint

  See paragraph 3.3.1 in chapter 3.3: Multiplicity adjustment

- Estimation of marginal means for the primary outcome measure for the two factors intake of vitamin B compound (no/yes) and intake of prednisolone (no/yes) with 95%CI

- 95% CI for the mean change score within all 4 factor combinations of vitamin B compound and prednisolone.

3.9.2 ADJUSTED ANALYSES

Within an adjusted analysis general linear models will be fitted for the primary endpoint, the change in symptom severity to day 28 as assessed by the specifically tailored total score. These explanatory models should serve to estimate effects of the factors vitamins and prednisolone by means of adjusted effect estimation.

The models will include the categorical predictor variables:

- Intake of vitamins (binary)
- Intake of prednisolone (binary)
- Study center (three level)
- Age group of tens
- Gender

As well as the covariates:

- Baseline symptom severity as assessed by the specifically tailored total score (PROMIS)
- Duration of Post-COVID-19 symptoms at baseline

3.9.3 SUBGROUP ANALYSES

Because the primary endpoint and many of the secondary endpoints in the study are subjective in the sense that based on questionnaires, possible problems associated with unblinding have to be considered. In order to measure the degree to which the blinding was successful in the study a post-trial, „blinding“ questionnaire is planned to quantify the patients‘ knowledge and study personal resp. belief about the administered treatment. For this all patients in the study are ask to guess which treatment they received.

The questionnaire admits the following five choices:

1: I believe to be in the placebo group
2: I believe to be in the prednisolone group
3: I believe to be in the vitamins group
4: I believe to be in the group with combined prednisolone and vitamins treatment
5: I don't know / I'm undecided about the treatment group (DK).

Generally, it can be expected that the effectiveness of the administration of vitamins or/and prednisolone depends on the inherent effects of the treatments *as well as* the patients' expectations. Therefore, within a secondary analysis the effectiveness of the treatments will be inferred by a kind of „like-for-like „comparisons.

Firstly, the patients were stratified, each stratum contains the patients who share the same combination of the intervention (the 4 treatment arms) and the same expressed *belief* regarding their group assignments (the 5 choices listed above). The distributions of the main outcome variable will be characterized by summarizing descriptive statistics in each of these 20 strata.

Subsequently subgroup analyses will be done. Within these subgroup analyses mean outcomes will be compared between patients received different medication but with the same response in the patient's feedback questionnaire. These are purely explorative, simple comparisons to get a rough impression whether there could actually be differences in the treatment effects across subgroups formed by patients' belief and whether there could be robust treatment effects.

Explicitly the mean primary outcome will be compared between patients randomised to treatment with vitamins B and patients randomised to placebo within the following subgroups:

- Belief in assignment to placebo
- Belief in assignment to vitamins B
- undecided about the treatment group (DK).

In the same way the mean primary outcome will be compared between patients randomised to treatment with prednisolone and patients randomised to placebo *within* the following subgroups:

- Belief in assignment to placebo
- Belief in assignment to prednisolone
- undecided about the treatment group (DK).

And finally the mean primary outcome will be compared between patients randomised to combined prednisolone and vitamins treatment and patients randomised to placebo *within* the following subgroups:

- Belief in assignment to placebo
- Belief in assignment to combined vitamins B and prednisolone
- undecided about the treatment group (DK).

Clearly partially small strata sizes are to be expected. Therefore, comparisons will be done, if indeed possible, by two- tailed t test / Mann-Whitney test / permutation test depending on sample sizes and properties of the observed distributions.

3.9.4 Treatment of Missing Data

For the calculation of the primary endpoint the free Health Measuring Scoring Service (HMSS) will be used to obtain T-scores for the 5 PROMIS Short Forms (Fatigue, Dyspnea Severity, Cognitive Function, Anxiety, Depression). To indicate when a patient did not answer an item, whether because it was skipped or not applicable, either the word "SKIP" or a blank field will be listed and with this the HMSS algorithm will deal with missing data at the calculation of T-scores.

For the estimation of treatment effects and the ANOVA modelling the following strategy will be applied in the analysis of the primary endpoint, based on the results from an extensive simulation study (N.R. Thompson et al. 2019):

- Firstly missing values for each of the 5 PROMIS Short Forms (Fatigue, Dyspnea Severity, Cognitive Function, Anxiety, Depression) will be imputated separately on the original scale by multiple imputation.
- Secondly for each patient the 5 T-scores were computed using the HMSS on the combined complete and imputed original scores in each case
- Finally the primary endpoint, the specifically tailored PROMIS total score will be calculated for each patient.

Beforehand the preferred method for multiple imputation is predictive mean matching. One sensitive analysis will be done by applying proportional odds logistic regression for multiple imputation. But in the case of unexpected missing data patterns or an unforeseen extent of missing values, e.g. determined by a blind review of the data at the end of the study, we provide the opportunity to update the strategy above for dealing with missing values and to add some additional *post hoc* sensitivity analyses (e.g. complete case

analysis, worst- and best case scenarios). This is consistent with the EMA Guideline on Missing Data in Confirmatory Clinical Trials [3].

The first and the second step of the strategy above will applied in the same manner for the 5 PROMIS subscores as secondary endpoints.

For all other secondary endpoints missing values will not be imputed instead complete case analysis will be done.

3.10 Other Sensitivity Analyses

Blinding assessment and interpreting data from questionnaires in the case of imperfect blinding

Because the primary endpoint and many of the secondary endpoints in the study are subjective in the sense that based on questionnaires, possible problems associated with unblinding have to be considered. Therefore, we will try to measure the degree to which the blinding was successful in the study by appropriate statistical methods. Blinding fails when patients become aware of the treatment arm they were assigned or when clinicians administering the treatment or assessing the state of disease becomes aware of the assignment for particular patients.

Therefore a post-trial, „blinding“ questionnaire is planned to quantify the patients‘ knowledge and study personal resp. belief about the administered treatment. For this the patients both in the active treatment groups and in the placebo group are ask to guess which treatment they received.

The questionnaire admits the following five choices:

1: I believe to be in the placebo group
2: I believe to be in the prednisolone group
3: I believe to be in the vitamins group
4: I believe to be in the group with combined prednisolone and vitamins treatment
5: I don't know / I'm undecided about the treatment group (DK)

For the analysis we will use two blinding indexes (BIs), James‘ BI and the generalized Bang blinding index by Williamson (FBI, 2014) and calculate point- and interval estimates (95% two-sided CI) for these indexes.

James‘ BI was developed already in 1996 and is well known. But this blinding index has received criticism because the very strong influence of the DK responses and that it generates only a single value for the entire study that means no possibility for an arm-specific blinding index. The generalized Bang blinding index by Williamson (FBI, 2014) is sensitive to both indicators of successful blinding: excessive DK responses or / and random guessing among non-DK responses. The method yields very interpretable arm-specific index values and is applicable to studies with any number of treatment arms (provided the sample sizes are large enough). Especially important, the method is appropriate for hypothesis testing, e.g., regarding random guessing. Under the null hypothesis of random guessing in the auxiliary questionnaire the expected value of each arm-specific FBIi (i=1, 2, 3, 4) is zero. The value of the FBIi can be interpreted as the proportion guessing correctly the own assigned arm beyond random balance.

For each single treatment arm unblinding may be claimed if the corresponding two-sided 95% CI does not cover 0. For the whole study claiming unblinding can be based on a two-sided simultaneous 95% CI with a multiple comparison correction. This blinding index is a generalization of Bang's blinding index (BI, 2004) which has advantages over the James index but is applicable only in the case of two treatments arms, contrary to the PreVitaCOV trial with 4 arms.

To detect unblinding possibly due to possible failure in protocols, the amount of blinding will be additionally investigated in each of the three centers and compared with one another. That means the binding indexes

will be calculated also for each center separately with corresponding 95% CI. Subsequently statistical pairwise comparisons will be done. Consider the case that the blinding assessment hints to a substantial level of unblinding that means the blinding in the study was not successful. In this case the main trial results, especially the primary outcome, change in symptom severity to day 28 as assessed by the specifically tailored total score based on PROMIS, needs to be interpreted carefully.

Generally, it can be expected that the effectiveness of the administration of vitamins or/and prednisolone depends on the inherent effects of the treatments as well as the patients' expectations. Therefore, within a secondary analysis the effectiveness of the treatments will be inferred by a kind of „like-for-like „comparisons.

Firstly, the patients were stratified, each stratum contains the patients who share the same combination of the intervention (the 4 treatment arms) and the same expressed belief regarding their group assignments (the 5 choices listed above). The distributions of the main outcome variable will be characterized by summarizing descriptive statistics in each of these 20 strata.

Subsequently subgroup analyses will be done. Within these subgroup analyses mean outcomes will be compared between patients received different medication but with the same response in the patient's feedback questionnaire. These are purely explorative, simple comparisons to get a rough impression whether there could actually be differences in the treatment effects across subgroups formed by patients' belief and whether there could be robust treatment effects.

Explicitely the mean primary outcome will be compared between patients randomised to treatment with vitamins B and patients randomised to placebo *within* the following subgroups:

- Belief in assignment to placebo
- Belief in assignment to vitamins B
- undecided about the treatment group (DK).

In the same way the mean primary outcome will be compared between patients randomised to treatment with prednisolone and patients randomised to placebo *within* the following subgroups:

- Belief in assignment to placebo
- Belief in assignment to prednisolone
- undecided about the treatment group (DK).

And finally the mean primary outcome will be compared between patients randomised to combined prednisolone and vitamins treatment and patients randomised to placebo *within* the following subgroups:

- Belief in assignment to placebo
- Belief in assignment to combined vitamins B and prednisolone
- undecided about the treatment group (DK).

Clearly partially small strata sizes are to be expected. Therefore, comparisons will be done, if indeed possible, by two- tailed t test / Mann-Whitney test / permutation test depending on sample sizes and properties of the observed distributions.

Nevertheless we define these 9 multiple, pairwise comparisons addressing the problem of possibly imperfect blinding as one family of tests and use the Tukey-Kramer test to control the familywise error rate (FWER) at the level $\alpha = 0.05$. This test is more powerful than e.g. the Bonferroni, Šidák or Scheffé test.

3.11 Analysis of Secondary Endpoints

3.11.1 Sum scores for questionnaires as well as data from the VAS for QoL and NRS for pain

For the sum scores from the 5 PROMIS® Short Form instruments on the original scale:

Fatigue 4a, Dyspnoea Severity 10a, Cognitive Function 4a, Emotional Distress-Anxiety 4a and Emotional Distress-Depression 4a

- Descriptive statistics, stratified by treatment group and visit (days 0, 5, 14, 21, 28, 60, 180) in tabular form: Mean, median, minimum, maximum
- For the change in sum score from day 0 (V1) to day 28 (V2): Nonparametric Randomisation-Based Analysis of Covarianz with one 4-Level factor treatment and covariate sum score at day 0.

For the sum scores from EQ-5D-5L (general quality of life), for PHQ-8 (measure of current depression), Chalder fatigue scale (measure of severity of tiredness) and the NRS for pain:

- Descriptive statistics, stratified by treatment group and visit (days 0, 5, 28, 60, 180) in tabular form: Mean, median, minimum, maximum
- For the change in sum score from day 0 to day 28: Nonparametric Randomisation-Based Analysis of Covarianz with one 4-Level factor treatment and covariate sum score at day 0.

For the VAS QoL:

- Descriptive statistics, stratified by treatment group and visit (days 0, 5, 28, 60, 180) in tabular form: Mean, median, minimum, maximum
- For the change in VAS QoL from day 0 to day 28: Two-factor factorial Analysis of Variance.

3.11.2 Ordinal secondary endpoints of Likert scale type

MYMOP2 individual items, EQ-5D-5L individual dimensions, Post-COVID-19 functional status scale (PCFS), Test of attentional performance (TAP) items:

- Absolut and relative frequencies will be given, stratified by visit (days 0, 5, 28, 60 and 180) and treatment group. Furthermore shift tables for the change from day 0 to day 28.

For analysing the effect of the treatment on the change in the distribution of the individual symptoms resp. items from day 0 to day 28 we used the Wald-test for an interaction effect within generalized linear modelling. Specifically ordinal logistic regression will be applied with treatment as between- and the visit (days 0, 28) as within subject factor. In case of limited sample size or sparse response categories we will put together neighbouring categories appropriately. In any case before using proportional odds models the proportional odds assumption will be evaluated. To assess the proportional odds assumption firstly hypothesis testing will be performed (Brant's Wald test). If the hypothesis of proportional odds is rejected by significance testing this will be investigated more closely (e.g. by dichotomizing the outcome variable) because such tests partly fail in controlling the type I error. If the proportional odds assumption is definitely violated partial proportional odds models or multinomial logistic regression will be used.

3.11.3 Physical exercise: 1-minute Sit-to-Stand-Test

Descriptive statistics, stratified by treatment group in tabular form (Mean, median, minimum, maximum)

- For day 0, day 28 and change from day 0 to day 28

- For the change in sum score from day 0 to day 28: Nonparametric Randomisation-Based Analysis of Covarianz with one 4-Level factor treatment and covariate sum score at day 0.

3.11.4 USE OF ON-DEMAND MEDICATION (FROM PATIENT DIARY)

a) Frequency tables for the type of on-demand medications (relevant in this study), stratified by treatment group.

   This will be done together with the PCI and the PI's.

b) Analyses of the total count of on-demand medication (if possible because of the frequency of applications):

Reported ongoing from day 1 to day 28. In tabular form, stratified by treatment groups descriptive statistics will be given for the numbers of days up to visit 2 (day 28) with at least one on-demand medication and the total counts of tablets / capsules for on-demand medication.

Confirmatory analysis for the secondary endpoint use of on-demand medication (patient diary): to compare the counts of on-demand medications within the period from baseline to day 28 between the four treatments negative binomial regression (NBR) models resp. zero-inflated negative binomial regression (ZINBR) models will be used. Within these models the count of using additional on-demand medication is the dependent variable, furthermore the models include the two binary main factors treatment with vitamin B compound, treatment with prednisolone as well the interaction factor. Using NBR models we can account for possible over-dispersion and using ZINBR models we can account for a possible elevated frequency of zero accounts in addition.

This modeling is carried out for:

- The number of days with on-demand medication
- The total number of tablets / capsules for on-demand medication

3.12 ANALYSIS OF SAFETY ENDPOINTS

3.12.1 NUMBER AND PROPORTION OF PATIENTS WITH WORSENING PC19 SYMPTOMS

Here the criteria for the determination what is to be considered an worsening of symptoms in each case will be carried out together with the PCI and the PI's before database closure and start of the final statistical analysis.

In table form and separately for the four treatment arms:

- Descriptive statistics for the counts of reported worsening PC19 symptoms resp. newly occurring PC19 symptoms from baseline to day 60 (phone call 2): mean, median, minimum, maximum.
- Numbers and proportions of patients with at least one worsening PC19 symptom resp. newly occurring PC19 symptom from baseline to day 60.

The null hypothesis of equal underlying proportions for all 4 treatment will be tested by a $\chi 2$-test with 3 degrees of freedom for the corresponding likelihood ratio test statistic (see Y. Mai and Z. Zhang 2017).

3.12.2 ADVERSE EVENTS

In table form and separately for the four treatment arms we will report:

- Total count of AE
- Numbers and proportions of patients with at least one AE from baseline to day 60.
- Absolute and relative frequencies of events (up to day 60) according to:

- MedDRA system organ classes (SOC coding with preceding definitions)
    - Seriousness (binary)
    - Severity grade (5 categories)
    - Therapy for event (binary)
    - Outcome (6 categories)
    - Causality (5 categories)
    - Action taken with trial medication (6 categories)

The null hypothesis of equal underlying proportions for all 4 treatment will be tested by a χ^2-test with 3 degrees of freedom for the corresponding likelihood ratio test statistic (see Y. Mai and Z. Zhang 2017).

3.12.3 SERIOUS ADVERSE EVENTS

A chronological detailed SAE report from the SAE Report Form in the eCRF (line listing) will be provided.

The following data will presented graphically and in table form (including all events up to day 60):

- Total count of SAE
- Mean number of SAEs per patient
- Proportion of patients with at least one SAE, point and interval estimation (95% confidence interval according to the Wilson score method)
- Absolute and relative frequencies of events according to:
    - Severity or CTC grade (5 categories)
    - Intensity (3 categories)
    - Treatment of SAE (3 categories)
    - Outcome of SAE (6 categories)
    - Action taken with trial medication (6 categories)

3.12.4 LABORATORY DATA AND VITAL SIGNS

For selected vital signs (in consultation with the PCI and the PI's) changes from baseline to the visits with recorded data in continuous laboratory parameters will be summarized separately over all patients in the four treatment groups by n, mean, median, minimum and maximum values.
Possible laboratory data which could be presented in table form:

- vitamin B12 status
    - Homocystein
    - MMA
    - HoloTC
- cytokines
- cortisol level (amendement)
- non-fasting glucose

Possible vital signs which could be presented in table form:

- pulse
- BP
- Respiratory rate

4 References

1. Kurt Kroenke, Timothy E. Stump, Chen X. Chen, , Jacob Kean, Matthew J. Bair, Teresa M. Damush, Erin E. Krebs, Patrick O. Monahan.
   Minimally Important Differences and Severity Thresholds are Estimated for the PROMIS Depression Scales from Three Randomized Clinical Trials
   J Affect Disord. 2020 April 01; 266: 100–108.
2. David Cella, PhD, Seung W. Choi, PhD, David M. Condon, PhD, Ben Schalet, PhD, Ron D. Hays, PhD, Nan E. Rothrock, PhD, Susan Yount, PhD, Karon F. Cook, PhD, Richard C. Gershon, PhD, Dagmar Amtmann, PhD, Darren A. DeWalt, MD, Paul A. Pilkonis, PhD, Arthur A. Stone, PhD, Kevin Weinfurt, PhD, Bryce B. Reeve, PhD
   PROMIS® Adult Health Profiles: Efficient Short-Form Measures of Seven Health Domains
   VALUE HEALTH. 2019; 22(5):537–544
3. EUROPEAN MEDICINES AGENCY.
   Guideline on Missing Data in Confirmatory Clinical Trials
   EMA/CPMP/EWP/1776/99 Rev. 1. 2010 July 02

5 EXAMPLES FOR PRESENTED TABLES

Adverse Event Reporting (AE)

Seriousness								
	Treatment arm 1		Treatment arm 2		Treatment arm 3		Treatment arm 4	
Serious?	Frequency	Percent	Frequency	Percent	Frequency	Percent	Frequency	Percent
yes								
no								

Severity Grade								
	Treatment arm 1		Treatment arm 2		Treatment arm 3		Treatment arm 4	
Grading	Frequency	Percent	Frequency	Percent	Frequency	Percent	Frequency	Percent
CTC grade 1								
CTC grade 2								
CTC grade 3								
CTC grade 4								
CTC grade 5								

Therapy for Event								
	Treatment arm 1		Treatment arm 2		Treatment arm 3		Treatment arm 4	
Therapy?	Frequency	Percent	Frequency	Percent	Frequency	Percent	Frequency	Percent
yes								
no								

Outcome								
	Treatment arm 1		Treatment arm 2		Treatment arm 3		Treatment arm 4	
Outcome	Frequency	Percent	Frequency	Percent	Frequency	Percent	Frequency	Percent
recovered/resolved								
recovering/resolving								
not recovered/not resolved								
not recovered/not resolved with sequelae								
fatal								
unknown								

Causality								
	Treatment arm 1		Treatment arm 2		Treatment arm 3		Treatment arm 4	
Causality	Frequency	Percent	Frequency	Percent				
certain								
probable								
possible								
unrelated / unlikely								
unknown								

Action Taken with Medication								
	Treatment arm 1		Treatment arm 2		Treatment arm 3		Treatment arm 4	
Action	Frequency	Percent	Frequency	Percent	Frequency	Percent	Frequency	Percent
none								
dose reduced temporarily								
dose reduced								
dose discontinued temporarily								

Serious Adverse Event Reporting (SAE)

additional tables

Seriousness Criteria / Reportable Reason								
	Treatment arm 1		Treatment arm 2		Treatment arm 3		Treatment arm 4	
Criteria / Reason	Frequency	Percent	Frequency	Percent	Frequency	Percent	Frequency	Percent
results in death								
life-threatening								
requires inpatient hospitalisation or prolongation								
results in persistent or significant isability/incapacity								
congenital anomaly/birth defect								
other medically important condition								

Intensity								
	Treatment arm 1		Treatment arm 2		Treatment arm 3		Treatment arm 4	
Intensity	Frequency	Percent	Frequency	Percent	Frequency	Percent	Frequency	Percent
mild								
moderate								
severe								

MedDRA system organ class								
	Treatment arm 1		Treatment arm 2		Treatment arm 3		Treatment arm 4	
Grading	Frequency	Percent	Frequency	Percent	Frequency	Percent	Frequency	Percent
SOC code 1								
SOC code 2								
SOC code 3								
SOC code 4								
SOC code 5								
SOC code 6								
SOC code 7								
SOC code 8								
SOC code 9								
SOC code 10								
SOC code 11								
SOC code 12								
SOC code 13								
SOC code 14								
SOC code 15								

**SOC code 16**								
**SOC code 17**								
**SOC code 18**								
**SOC code 19**								
**SOC code 20**								
**SOC code 21**								
**SOC code 22**								
**SOC code 23**								
**SOC code 24**								
**SOC code 25**								
**SOC code 26**								
**SOC code 27**								

Sum scores from the 5 PROMIS® Short Form instruments on the original scale (Examplary Fatigue 4a)

Fatigue 4a							
Treatment arm 1							
	Day 0	Day 5	Day 14	Day 21	Day 28	Day 60	Day 180
**N Valid**							
**N Missing**							
**Mean**							
**Median**							
**Minimum**							
**Maximum**							

Fatigue 4a							
Treatment arm 2							
	Day 0	Day 5	Day 14	Day 21	Day 28	Day 60	Day 180
**N Valid**							
**N Missing**							
**Mean**							
**Median**							
**Minimum**							
**Maximum**							

Fatigue 4a							
Treatment arm 3							
	Day 0	Day 5	Day 14	Day 21	Day 28	Day 60	Day 180
N Valid							
N Missing							
Mean							
Median							
Minimum							
Maximum							

Fatigue 4a							
Treatment arm 4							
	Day 0	Day 5	Day 14	Day 21	Day 28	Day 60	Day 180
N Valid							
N Missing							
Mean							
Median							
Minimum							
Maximum							

Results from the confirmatory analysis of the primary outcome measure

Descriptive Statistics

Dependent Variable: Change in the T-transformed tailored PROMIS score

Vitamin B	Prednisolone	Mean	Std. Deviation	N
no	no			
	yes			
	Total			
yes	no			
	yes			
	Total			
Total	0			
	1			
	Total			

Tests of Between-Subjects Effects

Dependent Variable: Change in the T-transformed tailored PROMIS score

Source	Type III Sum of Squares	df	Mean Square	F	Sig.	Partial Eta Squared
Corrected Model						
Intercept						
Vitamin B						
Prednisolone						
Vitamin B * Prednisolone						
Error						
Total						
Corrected Total						

Estimates

Dependent Variable: Change in the T-transformedtailored PROMIS score

Vitamin B	Mean	Std. Error	95% Confidence Interval Lower Bound	95% Confidence Interval Upper Bound
no				
yes				

Pairwise Comparisons

Dependent Variable: Change in the T-transformedtailored PROMIS score

(I) Vitamin B	(J) Vitamin B	Mean Difference (I-J)	Std. Error	Sig.	95% Confidence Interval for Difference Lower Bound
no	yes				
yes	no				

Pairwise Comparisons

Dependent Variable: Change in the T-transformedtailored PROMIS score

(I) Vitamin B	(J) Vitamin B	95% Confidence Interval for Difference Upper Bound
no	yes	
yes	no	

Estimates

Dependent Variable: Change in the T-transformedtailored PROMIS score

Prednisolone	Mean	Std. Error	95% Confidence Interval Lower Bound	95% Confidence Interval Upper Bound
no				
yes				

Pairwise Comparisons

Dependent Variable: Change in the T-transformedtailored PROMIS score

(I) Prednisolone	(J) Prednisolone	Mean Difference (I-J)	Std. Error	Sig.	95% Confidence Interval for Difference Lower Bound
no	yes				
yes	no				

Pairwise Comparisons

Dependent Variable: Change in the T-transformedtailored PROMIS score

(I) Prednisolone	(J) Prednisolone	95% Confidence Interval for Difference Upper Bound
no	yes	
yes	no	

Interaction Vitamin B * Prednisolone

Dependent Variable: Change in the T-transformedtailored PROMIS score

Vitamin B	Prednisolone	Mean	Std. Error	95% Confidence Interval	
				Lower Bound	Upper Bound
no	no				
	yes				
yes	no				
	yes				
