# Supplementary material for: Low-dose prednisolone and B vitamins for post-COVID-19 syndrome in Germany: the PreVitaCOV randomised clinical trial
Source: eClinicalMedicine. 2026 Oct 24;101:104264. doi: 10.1016/j.eclinm.2026.104264 (PMC13720163; doi:10.1016/j.eclinm.2026.104264)
Supplement: Supplementary file 3 [file mmc1.docx]

**Supplementary appendix**

**Table of contents**

eTable 1. Numbers needed to treat (NNT) for clinical improvement with prednisolone

eTable 2. Predominant symptoms and signs as reported by participants in the Measure Yourself Medical Outcome Profile (MYMOP)

eTable 3. Concomitant medication at baseline and day 28

eTable 4. Assessment of allocation concealment by treatment group at day 180

eTable 5. Baseline characteristics of first 100 participants (pilot trial cohort) by treatment group

eTable 6. Symptoms and symptom severity at baseline

eTable 7. Summary of all protocol versions and amendments (v1.0–v5.0)

eTable 8. Changes between versions 1.0 and 1.1 of the statistical analysis plan

eFigure 1. Changes from baseline in the Patient-Reported Outcomes Measurement Information System (PROMIS) total score (primary endpoint on day 28) and its five domains in patients who did and who did not receive prednisolone (regardless of vitamin B administration), day 0 to day180

eFigure 2. Predominant Severe Acute Respiratory Syndrome (SARS)-CoV-2 variants in Germany from 2020 to July 2024 - and the timing of SARS-CoV-2 infection, which the patients perceived as the onset of post-COVID symptoms

**eTable 1. Numbers needed to treat (NNT)^a^ for clinical improvement with prednisolone**

| Outcome measures^b^ | Range of the scale | Clinically relevant difference^c^ | Pred-  nisolone | Number of patients who … | | |  | NNT [95% confidence interval (CI)] to… | | |
| --- | --- | --- | --- | --- | --- | --- | --- | --- | --- | --- |
|  |  |  |  | **worsen** | **do not change** | **improve** |  | **achieve one improvement** | **prevent one worsening** | **either outcome** |
| PROMIS | 16.7 … 57.5 | ≥5 | yes no | 4 5 | 88 104 | 65 44 |  | 7·9 [4·3, 43·4] | – | 7·5 [4·0, 55·1] |
| MYMOP | 0 … 6 | ≥1 | yes no | 3 5 | 84 109 | 69 39 |  | 5·3 [3·4, 12·0] | – | 5·0 [3·2, 11·8] |
| EQ-5D-5L | –0.66 … 1.00 | ≥0.04 | yes no | 19 35 | 48 54 | 90 63 |  | 6·3 [3·7, 20·4] | 9·2 [5·3, 40·4] | 3·7 [2·3, 9·9] |
| PHQ-8 | 0 … 24 | ≥4 | yes no | 6 10 | 104 119 | 46 24 |  | 7·2 [4·4, 21·3] | – | 6·1 [3·6, 18·3] |
| Chalder Fatigue Scale | 0 … 33 | ≥4 | yes no | 4 6 | 76 102 | 77 45 |  | 5·1 [3·3, 11·3] | – | 4·8 [3·0, 10·7] |
| Pain | 0 … 10 | ≥2 | yes no | 18 28 | 106 99 | 33 25 |  | 21·9 [7·4, ∞] | 14·4 [6·8, ∞] | 8·7 [4·1, ∞] |
| Sit-to-Stand-test | ≥0 | ≥3 | yes no | 21 33 | 55 65 | 74 47 |  | 5·9 [3·6, 17·8] | 11·4 [5·7, ∞] | 3·9 [2·4, 11·2] |
| PCFS | 0 … 4 | any except 0→1, 1→0 | yes no | 14 13 | 86 108 | 57 32 |  | 6·5 [3·9, 19·2] | – | 6·7 [3·6, 46·9] |

^a^ The NNT with prednisolone treatment regimen to achieve clinically relevant improvement, prevent clinically relevant worsening, or either outcome from day 0 to day 28 in the primary and secondary outcomes, compared to placebo.

^b^ Patient-Reported Outcomes Measurement Information System (PROMIS): five-component overall score; for the responder analysis and calculation of numbers needed to treat, a more conservative threshold of ≥5 T-score points was used to define clinically meaningful improvement within an individual participant. A ≥5-point change corresponds to one-half of a standard deviation on the PROMIS T-score metric and has previously been proposed as sufficient to identify clinically meaningful within-person change across several PROMIS domains. Measure Yourself Medical Outcome Profile (MYMOP®): burden of the two subjectively most important symptoms; EQ-5D-5L: limitations in daily activities (EuroQoL, 5 dimensions, 5 levels); Patient Health Questionnaire-8 (PHQ-8): sum score of 8-item depression questionnaire; Fatigue: Chalder Fatigue Scale; Pain NRS: 10-point numeric rating scale of pain; Sit-to-Stand: number of stand-ups from sitting in 1 min; PCFS: 5-point, categorical post-COVID functional status scale (0-4); High values are favourable for EQ-5D-5L and Sit-to-Stand, low values are favorable for all other outcomes.

^c^ Clinically relevant differences based on:

**PROMIS:** Jensen RE, Moinpour CM, Potosky AL, et al. Responsiveness of 8 Patient-Reported Outcomes Measurement Information System (PROMIS) measures in a large, community-based cancer study cohort. Cancer. 2017;123(2):327–335. doi:10.1002/cncr.30354; Norman GR, Sloan JA, Wyrwich KW. Interpretation of changes in health-related quality of life: the remarkable universality of half a standard deviation. Med Care. 2003;41(5):582–592. doi:10.1097/01.MLR.0000062554.74615.4C.

**MYMOP:** Hermann K, Kraus K, Herrmann K, Joos S. A brief patient-reported outcome instrument for primary care: German translation and validation of the Measure Yourself Medical Outcome Profile (MYMOP). Health Qual Life Outcomes 2014;12(1):112. doi: 10.1186/s12955-014-0112-5.

**EQ-5D-5L:** Bourke S, Bennett B, Oluboyede Y, et al. Estimating the minimally important difference for the EQ-5D-5L and EORTC QLQ-C30 in cancer. Health Qual Life Outcomes. 2024;22(1):81. doi: 10.1186/s12955-024-02294-3.

**PHQ-8:** Löwe B, Unützer J, Callahan CM, Perkins AJ, Kroenke K. Monitoring depression treatment outcomes with the patient health questionnaire-9. Med Care. 2004;42(12):1194-201. doi: 10.1097/00005650-200412000-00006.

**Chalder Fatigue Scale:** White PD, Goldsmith KA, Johnson AL, et al. Comparison of adaptive pacing therapy, cognitive behaviour therapy, graded exercise therapy, and specialist medical care for chronic fatigue syndrome (PACE): a randomised trial. Lancet. 2011;377(9768):823-36. doi: 10.1016/S0140-6736(11)60096-2.

**Pain (Numeric Rating Scale):** Dworkin RH, Turk DC, Wyrwich KW, et al. Interpreting the clinical importance of treatment outcomes in chronic pain clinical trials: IMMPACT recommendations. J Pain. 2008;9(2):105-21. doi: 10.1016/j.jpain.2007.09.005.

**Sit-to-Stand-test:** Vaidya T, de Bisschop C, Beaumont M, et al. Is the 1-minute sit-to-stand test a good tool for the evaluation of the impact of pulmonary rehabilitation? Determination of the minimal important difference in COPD. Int J Chron Obstruct Pulmon Dis. 2016;11(1):2609-2616. doi: 10.2147/COPD.S115439.

**PCFS (Post-COVID-19 Functional Status Scale):** Machado FVC, Meys R, Delbressine JM, et al. Construct validity of the Post-COVID-19 Functional Status Scale in adult subjects with COVID-19. Health Qual Life Outcomes. 2021;19(1):40. doi: 10.1186/s12955-021-01691-2.

**eTable 2. Predominant symptoms and signs as reported by participants in the Measure Yourself Medical Outcome Profile (MYMOP)**

| Category of problem / symptom | Total No. (%) of reports | No. (%) of patients who reported problems with… | No. of patients (%) with improvement by ≥1 point at day 28 | |
| --- | --- | --- | --- | --- |
|  |  |  | **did not receive prednisolone** | **received prednisolone** |
| Number of reports | 606 (=100%) |  |  |  |
| Number of patients who reported problems with… |  | 309 (=100%) | 153 (=100%) | 156 (=100%) |
|  |  |  |  |  |
| Fatigue | 184 (30) | 179 (58) | 35 (23) | 49 (31) |
| Concentration difficulties | 84 (14) | 84 (27) | 16 (11) | 21 (14) |
| Pain | 71 (12) | 66 (21) | 15 (10) | 16 (10) |
| Cognitive impairment | 64 (11) | 61 (20) | 13 (9) | 19 (12) |
| Physical exhaustion | 49 (8) | 47 (15) | 8 (5) | 15 (10) |
| Breathing difficulties | 44 (7) | 44 (14) | 10 (7) | 10 (6) |
| Brain fog | 23 (4) | 23 (7) | 9 (6) | 4 (3) |
| Post-exertional malaise (PEM) | 15 (3) | 15 (5) | 4 (3) | 5 (3) |
| Sleep problems | 11 (2) | 11 (4) | 0 | 4 (3) |
| Depressive symptoms | 9 (2) | 9 (3) | 2 (1) | 3 (2) |
| Dizziness | 9 (2) | 9 (3) | 1 (<1) | 3 (2) |
| Heart problems | 6 (1) | 6 (2) | 1 (<1) | 1 (<1) |
| Tinnitus | 5 (<1) | 5 (2) | 1 (<1) | 2 (1) |
| Anxiety | 4 (<1) | 4 (1) | 1 (<1) | 1 (<1) |
| Other | 28 (5) | 28 (9) | 3 (2) | 10 (6) |

MYMOP (Measure Yourself Medical Outcome Profile), participants reported 1 to 2 symptoms and scored their severity: 0 (as good as it could possibly be) to 6 (as bad as it could possibly be). The frequencies of total reports and the number of patients reporting are presented in different columns. The last two columns show the frequency of the respective categories that improved by at least one grade in patients who received prednisolone compared to those who did not.

**eTable 3. Concomitant medication at baseline and day 28**

|  | Overall  N = 315 | Prednisolone N = 78 | B vitamins  N = 76 | Prednisolone+ B vitamins N = 81 | Placebo  N = 80 |
| --- | --- | --- | --- | --- | --- |
| **Vitamins (A11)** |  |  |  |  |  |
| Baseline | 73 (23·2%) | 22 (28·2%) | 21 (27·6%) | 17 (21·0%) | 13 (16·3%) |
| Day 28* | 72 (23·3%) | 22 (29·0%) | 21 (28·4%) | 17 (21·0%) | 12 (15·4%) |
| **Anti-inflammatory and antirheumatic products, non-steroids (M01)** |  |  |  |  |  |
| Baseline | 67 (21·3%) | 18 (23·1%) | 17 (22·4%) | 14 (17·3%) | 18 (23·0%) |
| Day 28* | 66 (21·4%) | 18 (23·7%) | 17 (23·0%) | 14 (17·3%) | 17 (21·8%) |
| **Psychoanaleptics (N06)** |  |  |  |  |  |
| Baseline | 66 (21·0%) | 16 (20·5%) | 14 (18·4%) | 17 (21·0%) | 19 (23·8%) |
| Day 28* | 69 (22·3%) | 17 (22·4%) | 16 (21·6%) | 16 (19·8%) | 20 (25·6%) |
| **Drugs for obstructive airway diseases (R03)** |  |  |  |  |  |
| Baseline | 65 (20·6%) | 15 (19·2%) | 15 (19·7%) | 13 (16·1%) | 22 (27·5%) |
| Day 28* | 66 (21·4%) | 14 (18·4%) | 16 (21·6%) | 14 (17·3%) | 22 (28·2%) |
| **Analgesics (N02)** |  |  |  |  |  |
| Baseline | 61 (19·4%) | 16 (20·5%) | 20 (26·3%) | 14 (17·3%) | 11 (13·8%) |
| Day 28* | 64 (20·7%) | 16 (21·1%) | 21 (28·4%) | 15 (18·5%) | 12 (15·4%) |
| **Thyroid therapy (H03)** |  |  |  |  |  |
| Baseline | 48 (15·2%) | 7 (9·0%) | 15 (19·7%) | 12 (14·8%) | 14 (17·5%) |
| Day 28* | 48 (15·5%) | 7 (9·2%) | 15 (20·3%) | 12 (14·8%) | 14 (18·0%) |

* for n = 6 participants, no information on medication at day 28 was available

**eTable 4. Assessment of allocation concealment by treatment group at day 180**

| Patients' assumption of their allocation* | Overall  N = 299 | Prednisolone  N = 74 | B vitamins  N = 71 | Prednisolone+ B vitamins  N = 78 | Placebo  N = 76 |
| --- | --- | --- | --- | --- | --- |
| Prednisolone | 74 (25%) | 27 (36%) | 9 (13%) | 20 (26%) | 18 (24%) |
| B vitamins | 35 (12%) | 9 (12%) | 12 (17%) | 10 (13%) | 4 (5·3%) |
| Prednisolone + B vitamins | 33 (11%) | 6 (8·1%) | 11 (15%) | 11 (14%) | 5 (6·6%) |
| Placebo | 86 (29%) | 16 (22%) | 22 (31%) | 20 (26%) | 28 (37%) |
| Do not know | 71 (24%) | 16 (22%) | 17 (24%) | 17 (22%) | 21 (28%) |
| Generalised Bang Blinding Index |  | 0·22 | -0·03 | -0·07 | 0·25 |
|  |  |  |  |  |  |
| **Assumption of the centre regarding the patient's allocation*** | **Overall**  N = 300 | **Prednisolone** N = 74 | **B vitamins** N = 71 | **Prednisolone+ B vitamins** N = 78 | **Placebo**  N = 77 |
| Prednisolone | 38 (13%) | 14 (19%) | 5 (7·0%) | 9 (12%) | 10 (13%) |
| B vitamins | 43 (14%) | 8 (11%) | 13 (18%) | 9 (12%) | 13 (17%) |
| Prednisolone + B vitamins | 41 (14%) | 11 (15%) | 10 (14%) | 16 (21%) | 4 (5·2%) |
| Placebo | 51 (17%) | 10 (14%) | 11 (15%) | 12 (15%) | 18 (23%) |
| Do not know | 127 (42%) | 31 (42%) | 32 (45%) | 32 (41%) | 32 (42%) |
| Generalised Bang Blinding Index |  | 0·06 | 0·06 | 0·08 | 0·12 |

*** Number (%) of treatment allocation guesses by patients and study teams; each patient and each participating centre provided one guess.** The probability for each assumption is 25%.

**eTable 5. Baseline characteristics of first 100 participants (pilot trial cohort) by treatment group**

| **Characteristic** | **Overall**  N = 100 | **Prednisolone**  N = 25 | **B vitamins**  N = 25 | **Prednisolone**  **+ B vitamins**  N = 25 | **Placebo**  N = 25 |
| --- | --- | --- | --- | --- | --- |
| **Age, mean (standard deviation (SD)), years** | 42 (12) | 42 (14) | 39 (11) | 44 (9) | 45 (12) |
| **Sex** |  |  |  |  |  |
| Female | 71 (71%) | 14 (56%) | 20 (80%) | 20 (80%) | 17 (68%) |
| Male | 29 (29%) | 11 (44%) | 5 (20%) | 5 (20%) | 8 (32%) |
| **Living with partner** | 74 (74%) | 19 (76%) | 15 (60%) | 19 (76%) | 21 (84%) |
| **German as native language** | 96 (96%) | 24 (96%) | 25 (100%) | 23 (92%) | 24 (96%) |
| **General higher education*** | 45 (45%) | 10 (40%) | 11 (44%) | 9 (36%) | 15 (60%) |
| **Employed** | 81 (81%) | 18 (72%) | 20 (80%) | 23 (92%) | 20 (80%) |
| Of these: currently on sick leave | 38 (47%) | 10 (56%) | 9 (45%) | 11 (48%) | 8 (40%) |
| Weeks on sick leave due to Post-COVID, median (interquartile range (IQR)) | 26 (10, 46) | 34 (8, 47) | 24 (16, 37) | 30 (14, 52) | 22 (9, 40) |
| **Duration of Post-COVID symptoms, median (IQR), weeks** | 53 (34, 79) | 55 (32, 96) | 52 (34, 68) | 55 (36, 71) | 53 (32, 93) |
| **No. of applied treatments for post-COVID, mean (SD)** | 2·3 (2·0) | 2·5 (2·1) | 2·3 (1·8) | 2·2 (1·9) | 2·2 (2·1) |
| **Received at least 1 dose of COVID-19 vaccine** | 97 (97%) | 24 (96%) | 25 (100%) | 23 (92%) | 25 (100%) |
| **Smoking** | 12 (12%) | 4 (16%) | 2 (8%) | 5 (20%) | 1 (4%) |
| **No. of comorbidities** |  |  |  |  |  |
| None | 20 (20%) | 5 (20%) | 2 (8%) | 9 (36%) | 4 (16%) |
| One | 24 (24%) | 6 (24%) | 5 (20%) | 6 (24%) | 7 (28%) |
| Two or more | 56 (56%) | 14 (56%) | 18 (72%) | 10 (40%) | 14 (56%) |
| **Comorbidities** |  |  |  |  |  |
| Body mass index ≥ 30 | 21 (21%) | 6 (24%) | 5 (20%) | 4 (16%) | 6 (24%) |
| Chronic respiratory diseases | 31 (31%) | 9 (36%) | 9 (36%) | 5 (20%) | 8 (32%) |
| Cardiovascular diseases | 16 (16%) | 4 (16%) | 1 (4%) | 4 (16%) | 7 (28%) |
| Psychiatric disorder | 17 (17%) | 5 (20%) | 5 (20%) | 2 (8%) | 5 (20%) |
| *General or participant-specific higher education entrance qualification, equivalent to A-levels | | | | | |

**eTable 6. Symptoms and symptom severity at baseline**

|  | Prednisolone  N = 78 | B vitamins  N = 76 | Prednisolone  + B vitamins  N = 81 | Placebo  N = 80 |
| --- | --- | --- | --- | --- |
| **PROMIS total score, mean (standard deviation (SD))** | 36·3 (5·6) | 37·6 (5·7) | 36·9 (5·7) | 36·5 (4·9) |
| **PROMIS Fatigue, mean (SD)** | 64 (7) | 63 (8) | 64 (8) | 64 (6) |
| **PROMIS Anxiety, mean (SD)** | 55 (8) | 55 (8) | 56 (9) | 53 (7) |
| **PROMIS Depression, mean (SD)** | 54 (8) | 55 (8) | 57 (9) | 55 (8) |
| **PROMIS Dyspnoea, mean (SD)** | 45 (9) | 48 (8) | 44 (9) | 46 (8) |
| **PROMIS Cognition Score, mean (SD)** | 37 (8) | 33 (7) | 35 (8) | 35 (8) |
| **MYMOP symptoms, n (%):** |  |  |  |  |
| Fatigue | 49 (62·8%) | 43 (58·1%) | 37 (47·4%) | 50 (63·3%) |
| Concentration difficulties | 25 (32·1%) | 19 (25·7%) | 20 (25·6%) | 20 (25·3%) |
| Pain | 15 (19·2%) | 18 (24·3%) | 15 (19·2%) | 18 (22·8%) |
| Cognitive impairment | 17 (21·8%) | 16 (21·6%) | 17 (21·8%) | 11 (13·9%) |
| Physical exhaustion | 11 (14·1%) | 8 (10·8%) | 13 (16·7%) | 15 (19%) |
| Breathing difficulties | 7 (9%) | 14 (18·9%) | 10 (12·8%) | 13 (16·5%) |
| Brain fog | 4 (5·1%) | 4 (5·4%) | 5 (6·4%) | 10 (12·7%) |
| Post-exertional malaise (PEM) | 2 (2·6%) | 4 (5·4%) | 5 (6·4%) | 4 (5·1%) |
| Sleep problems | 3 (3·8%) | 2 (2·7%) | 5 (6·4%) | 1 (1·3%) |
| Depressive symptoms | 4 (5·1%) | 2 (2·7%) | 2 (2·6%) | 1 (1·3%) |
| Dizziness | 2 (2·6%) | 3 (4·1%) | 4 (5·1%) | 0 |
| Heart problems | 2 (2·6%) | 0 | 3 (3·8%) | 1 (1·3%) |
| Tinnitus | 2 (2·6%) | 1 (1·4%) | 1 (1·3%) | 1 (1·3%) |
| Anxiety | 0 | 2 (2·7%) | 1 (1·3%) | 1 (1·3%) |
| Other | 7 (9%) | 9 (12·2%) | 9 (11·5%) | 3 (3·8%) |
| Chalder Fatigue Scale, mean (SD) | 24·4 (5·0) | 25·8 (5·8) | 25·6 (5·3) | 24·8 (4·6) |
| NRS Pain Scale, mean (SD) | 3·0 (2·4) | 2·8 (2·3) | 2·9 (2·4) | 2·8 (2·2) |
| PHQ-8, mean (SD) | 10·7 (4·1) | 11·4 (4·5) | 11·5 (4·4) | 10·7 (4·2) |

PROMIS: Patient-Reported Outcomes Measurement Information System; Measure Yourself Medical Outcome Profile (MYMOP®): burden of the two subjectively most important symptoms, the figure given represents the number of patients with a corresponding mention; NRS Pain Scale: 10-point numeric rating scale of pain; Patient Health Questionnaire-8 (PHQ-8): sum score of 8-item depression questionnaire

**eTable 7. Summary of all protocol versions and amendments (v1.0–v5.0)**

| **Version** | **Version date** | **Regulatory status** | **Approving bodies and dates of approval** | **Principal changes from the previous version** |
| --- | --- | --- | --- | --- |
| **1.0** | Not dated | Internal pre-submission draft (not submitted) | Not applicable (not submitted). | Working draft. This version was not submitted to the ethics committee or the competent authority; the first submitted version was Version 2.0. |
| **2.0** | 28 Jun 2022 | First submitted version. Application for a favourable opinion to the lead ethics committee (Medical Faculty, University of Würzburg) under §42 AMG; application to the BfArM in parallel. | None. The lead ethics committee (4 Aug 2022) and the BfArM (26 Aug 2022) requested revisions, which led to Version 3.0. | First version submitted to the authorities (registered 30 Jun 2022). Copies were sent to the Kiel and Tübingen ethics committees on 30 Jun 2022. |
| **3.0** | 30 Sep 2022 | First approved version. | Lead ethics committee (Ethics Committee of the Medical Faculty, University of Würzburg, ref. 110/22_ff-am): favourable opinion on 6 Oct 2022, issued in consultation with the participating ethics committees of Kiel (ref. B 265/22/RE) and Tübingen (ref. 450/2022AMG2). BfArM (ref. 61-3910-4045523): authorisation on 14 Oct 2022. | Revision in response to the supplementary requirements of the lead ethics committee (request of 4 Aug 2022), the Tübingen ethics committee, and the competent authority (BfArM).  Revised the biometric/statistical section and updated the patient information, informed-consent and general-practitioner documents.  First participant enrolled on 13 Jan 2023. |
| **4.0** | 20 Mar 2023 | Amendment I. Applications dated 24 Mar 2023 (ethics committees) and 19 May 2023 (BfArM). | Lead ethics committee: favourable opinion on 2 May 2023, issued in consultation with the participating ethics committees of Kiel and Tübingen. Implementation of the committee’s remarks confirmed on 10 Aug 2023. BfArM: approval on 5 Jun 2023. | Revised the accepted proof of SARS-CoV-2 infection: in addition to PCR and antibody tests, confirmation of the infection by the patient’s general practitioner on a study-specific form was introduced.  Refined the prior vitamin-B exclusion: B1, B6 or B12 at a dose corresponding to the study medication for at least 7 days, or any parenteral use since the end of the acute phase of COVID-19, remained exclusionary; lower-dose supplements had to be stopped at least 4 weeks before inclusion.  Removed arterial hypertension as an exclusion criterion and introduced a one-week home blood-pressure assessment at the start of participation.  Added measurement of the baseline serum cortisol concentration.  Added information for participants on the possibility of impaired fitness to drive.  Administrative change: transfer of the study pharmacy from Charité Berlin to the University Medical Center Hamburg-Eppendorf (new labels and IMPDs). |
| **5.0** | 28 Aug 2023 | Amendment II. Applications dated 30 Aug 2023. Final version. | Lead ethics committee: favourable opinion on 5 Oct 2023, issued in consultation with the participating ethics committees of Kiel and Tübingen. BfArM: approval on 4 Oct 2023. | Relaxed the concomitant-medication exclusions to allow participants taking oral contraceptives or ACE inhibitors, given the short treatment duration and the low likelihood of clinically relevant interactions.  Revised the study-specific adverse-event definition so that the expected natural fluctuation of post-COVID symptoms was no longer automatically recorded as an adverse event; the original definition had counted any worsening of post-COVID-19 symptoms as an adverse event. |

*ACE, angiotensin-converting enzyme; AMG, Arzneimittelgesetz (German Medicinal Products Act); BfArM, Federal Institute for Drugs and Medical Devices; IMPD, investigational medicinal product dossier; PCR, polymerase chain reaction.*

**eTable 8. Changes between versions 1.0 and 1.1 of the statistical analysis plan**

| **Section** | **Change from version 1.0 to version 1.1** | **Type of change** |
| --- | --- | --- |
| **Title page and section 1 (administrative information)** | Version number and date updated from 1.0 (11 Oct 2023) to 1.1 (18 Jan 2024). Date of the BfArM authorisation of protocol version 3.0 corrected from 14 Oct 2023 to 14 Oct 2022. | Update, correction |
| **Section 2.2 (patient population)** | Eligibility criteria aligned with the final protocol version, with a note referring to the last protocol version. Acetylsalicylic acid and indometacin are listed together with the non-steroidal anti-inflammatory drugs (version 1.0: acetylsalicylic acid listed with the anticoagulants). The exclusion of other drugs with possible interactions was restricted to clinically relevant interactions, as judged by the clinical investigator. | Alignment with protocol version 5.0 |
| **Section 2.3.2 (secondary endpoints)** | The secondary endpoints of the pilot phase apply only if the trial had not proceeded to the confirmatory phase. Physical examination (auscultation of chest and heart, orientating neurological examination, lymph node status) was removed from the secondary endpoints of both phases. | Clarification, removal |
| **Section 3.1 (general principles)** | If the criteria for continuation after the pilot phase are met, analyses at that stage beyond the primary endpoint of the pilot phase are restricted to combined descriptions of baseline characteristics across all four groups (version 1.0: no analysis of secondary endpoints at that stage). The statement on plausibility and completeness checks was moved to the beginning of the section, and a reference to the endpoints of the confirmatory phase was added. | Clarification |
| **Section 3.4 (data sets to be analysed)** | Statement added that the primary analysis follows the intention-to-treat principle within the full analysis set, with participants analysed according to their randomised treatment. The definitions of the full analysis set and the per-protocol analysis set are unchanged. | Clarification |
| **Section 3.6 (patient characteristics and baseline comparisons)** | Physical fitness specified as the 1-minute sit-to-stand test, and physical examination specified as auscultation of chest and heart, orientating neurological examination and lymph node status. | Clarification |
| **Section 3.9.2 (adjusted analyses)** | Gender added as a categorical predictor in the adjusted general linear models for the primary endpoint. | Addition |
| **Section 3.9.4 (treatment of missing data)** | Provision added that, in the case of unexpected missing-data patterns or an unforeseen extent of missing values (eg, determined by a blind review of the data at the end of the study), the strategy for handling missing values may be updated and post-hoc sensitivity analyses added (eg, complete-case analysis, worst-case and best-case scenarios), in line with the EMA guideline on missing data in confirmatory clinical trials. | Addition |
| **Section 3.11 (analysis of secondary endpoints)** | Former section 3.11.3 on the analysis of physical examination findings deleted and subsequent sections renumbered. For on-demand medication (section 3.11.4), frequency tables by type of medication and treatment group were added, and the count analyses are done if the frequency of use permits. | Removal, addition |
| **Section 3.12 (analysis of safety endpoints)** | Criteria for worsening of post-COVID-19 symptoms are to be defined together with the coordinating investigator and the principal investigators before database closure and the start of the final analysis. Reporting of adverse events by MedDRA system organ class added, with a corresponding example table in section 5. Presentation of laboratory data and vital signs changed from a fixed list to a selection made in consultation with the investigators. | Addition, clarification |
| **Section 4 (references)** | EMA guideline on missing data in confirmatory clinical trials added. | Addition |
| **Throughout** | Editorial changes (British spelling, tense, typographical corrections). | Editorial |

*Version 1.0 is dated 11 Oct 2023 and version 1.1 is dated 18 Jan 2024. Both versions were finalised while recruitment was ongoing (January 2023 to July 2024), before database lock and unblinding. BfArM, Federal Institute for Drugs and Medical Devices; EMA, European Medicines Agency; MedDRA, Medical Dictionary for Regulatory Activities.*

**eFigure 1. Changes from baseline in the Patient-Reported Outcomes Measurement Information System (PROMIS) total score (primary endpoint on day 28) and its five domains in patients who did and who did not receive prednisolone (regardless of vitamin B administration), day 0 to day180**

| ○ Patients who did not receive prednisolone ● Patients who received prednisolone | |
| --- | --- |
|  |  |
|  |  |
|  |  |

Data are groupwise mean changes from day 0 in the respective T-scores (calibrations of raw values based on population reference data) and their 95% confidence intervals. P-values refer to the null hypotheses that mean changes, adjusted for the baseline values, are the same in patients who did and who did not receive prednisolone (not adjusted for multiple tests).

**eFigure 2. Predominant Severe Acute Respiratory Syndrome (SARS)-CoV-2 variants in Germany from 2020 to July 2024 - and the timing of SARS-CoV-2 infection, which the patients perceived as the onset of post-COVID symptoms**

Participants were recruited between January 2023 and July 2024, but the infection considered to have caused their post-COVID condition had occurred at least three months before enrolment. The figure therefore covers the ancestral SARS-CoV-2 lineages and the subsequent Alpha-, Delta-, and Omicron-dominant periods. Source: Robert Koch Institute. PCR: Polymerase Chain Reaction.
